# Compositional Tuning of Magnetic Properties in a Series of Transition Metal Site-Deficient UCo_*x*_Bi_2_ and UNi_*x*_Bi_2_ Phases

**DOI:** 10.1021/acs.inorgchem.5c05232

**Published:** 2026-01-03

**Authors:** Hope A. Long, Hope E. Smith, Gregory Morrison, Vladislav V. Klepov

**Affiliations:** † Department of Chemistry, 1355 University of Georgia, Athens, Georgia 30602, United States; ‡ Department of Chemistry and Biochemistry, 2629 University of South Carolina, Columbia, South Carolina 29208, United States; § Center for Hierarchical Waste Form Materials, and Department of Chemistry and Biochemistry, University of South Carolina, Columbia, South Carolina 29208, United States

## Abstract

Varying the electronic structure of topological materials through aliovalent substitution is a primary approach to tuning their physical properties. Unlike substitution, metal site deficiency intrinsic to some structure types, including HfCuSi_2_-type, has rarely been employed for controlling the properties of topological phases. In this report, we describe the synthesis and characterization of two new series of compounds, UCo_*x*_Bi_2_ and UNi_*x*_Bi_2_, which demonstrate the variation of transition metal content through synthetic conditions. Magnetic measurements reveal the dependence between the extent of transition metal incorporation and the magnetism of the resulting phase. DFT calculations demonstrated the ability to model their formation and predict the stability ranges of transition metal-site deficient compounds.

## Graphical abstract

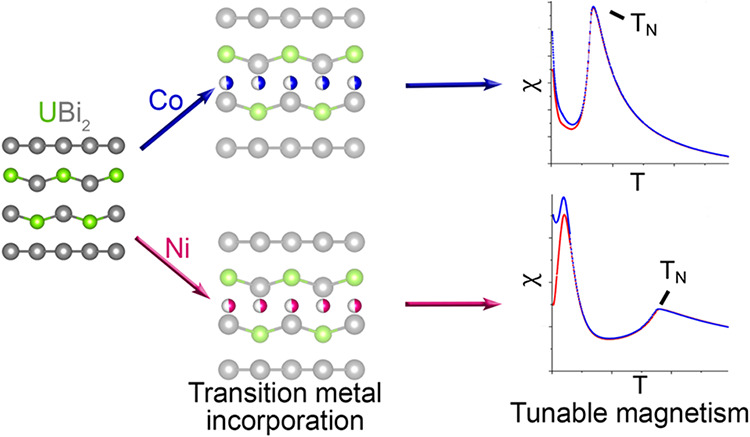

## Introduction

The exploration of quantum topological materials has opened new avenues in the realms of solid-state chemistry and condensed matter physics.^1–3^ Topological materials are a class of quantum materials that exhibit unique electronic properties governed by the topology of their electronic band structure. Unlike conventional materials, their behavior is not solely determined by symmetry or composition but by how electronic states are connected through space, meaning the inert pair effect and spin–orbit coupling are also key components of a material’s topological nature.^4^ This gives rise to robust surface or edge states that are protected against scattering by defects or disorder.^5^ These materials include topological insulators, whose surfaces are conducting but the interior, or bulk, is insulating, as well as topological semimetals and topological superconductors.^4,6^ These materials have unveiled remarkable, novel phenomena that hold great potential for advancing various technological applications such as energy conversion and storage.^7,8^ Research in this field continues to grow rapidly due to both fundamental interest and technological potential.

One group of topological materials is that with a crystal structure containing a planar square net sublayer. Overlap between the p_*x*_ and p_*y*_ orbitals of these structural units can give rise to Dirac crossings with linear dispersions in their band structures along the Γ-M and Γ-X symmetry lines,^9–12^ leading to a variety of novel physical and electronic properties.^13,14^ These band crossings are undisturbed by the other electronic states provided that the square net atoms are sufficiently isolated from the surrounding atoms in the crystal structure. To quantify this degree of isolation, Schoop et al. proposed a tolerance factor, *d*_sq_/*d*, which shows the ratio between the distances within the square nets, *d*_sq_, and between the square net atoms and neighboring atoms in the structure, *d*.^13^ The square nets are considered sufficiently isolated from other atoms in phases with low tolerance factors, *d*_sq_/*d* < 0.95, which give rise to rather “clean” Dirac points.^15^ The HfCuSi_2_ structure type is known to contain such structural units.^16,17^ It also has the enticing ability to accommodate variable amounts of a transition metal (*M*) in *AM*_*x*_ *Pn*_2_ compositions (*A* = lanthanide or uranium, *M* = transition metals, *Pn* = pnictides)^18,19^ which, as substantiated by our previous work,^20^ affects various properties, including structure and magnetism. Pnictogen compounds of this structure type serve as potential topological materials that merge topological properties stemming from the *Pn* square nets with modifiable orbital overlap and magnetism arising from the rare earth metal (*A*) and *M* sites.^19,21,22^

Both the identity and extent of transition metal incorporation in the Cu-site of these HfCuSi_2_-type compounds significantly influence their physical properties, including crystallographic and magnetic properties. In one case, that of LaMn_*x*_Sb_2_, a slight differentiation in transition metal content was observed to alter the crystal structure, wherein the symmetry was found to change from the *P*4/*nmm* when *x* ≥ 0.79 to an *I*4̅2*m* space group when *x* < 0.79.^23^ This change in symmetry is a result of the difference in vacancy ordering on the *M* site. The *P*4*/nmm* phase has a single *M* site with a set occupancy whereas the lower symmetry phase has three independent *M* sites, each with a different occupancy.

In these HfCuSi_2_-type materials, site deficiencies play a crucial role in controlling the electron count and tuning the Fermi level.^12,13^ These deficiencies are also known to affect the magnetic properties of the compound in a variety of ways, such as promoting metamagnetic transitions, altering magnetic ordering, and shifting transition temperatures.^20,23,24^ Although a few such cases have been reported, they remain scarce, leaving many fundamental questions unresolved. The limited number of studies has hindered a comprehensive understanding of how Cu-site vacancies influence the overall physical properties and has consequently impeded progress in the overall development and advancement of these materials.

Herein, we report on two new series of HfCuSi_2_-type compounds with site deficiency on the Cu site. We performed flux crystal growth of the new phases using Bi as a flux. The resulting single crystalline phases show a dependence of the product on the initial transition metal concentration. In the UCo_*x*_Bi_2_ series, compositions with *x* < 0.5 can be obtained, whereas the presence of the competing U_3_Ni_3_Bi_4_ phase limits the stability range of the Ni phases, UNi_*x*_Bi_2_, to *x* ≤ 0.3. We found that the stability ranges of these phases can be satisfactorily predicted by DFT calculations. Magnetic susceptibility measurements showed that the Néel temperatures vary as a function of transition metal content, in which they decrease as the occupancy increases, consistent with weakened exchange interactions along the *c*-axis. Overall, this work demonstrates how structural and magnetic properties can be systematically tuned through controlled transition metal incorporation and, thus, provide a foundation for the targeted design of correlated and potentially topological materials.

## Results and Discussion

### Synthesis

To synthesize large single crystals of a sufficient size for property measurements, we have performed a series of reactions to optimize the synthetic conditions. The target phases were expected to crystallize in the HfCuSi_2_ structure type, which is known to exhibit a deficiency on the *M* site.^24–26^ In an effort to achieve maximum *M* loading, we employed an excess of Co or Ni, respectively, in the starting reaction mixture. The Co-containing reaction yielded very small plate crystals, which were found to be UCo_0.4_Bi_2_ by single crystal X-ray diffraction (SCXRD). After a series of optimization steps, we found that reactions with a 1:3:13 U:Co:Bi ratio resulted in good-quality single crystals (Figure 1), up to 3 × 1 × 0.3 mm^3^, that were suitable for property measurements. This optimized crystal growth profile was used as a basis for the Ni system, in which the temperature profile was kept constant. Unlike its Co counterpart, the U–Ni–Bi system contains a thermodynamically stable U_3_Ni_3_Bi_4_ competing phase,^27^ which prevents the formation of UNi_*x*_Bi_2_ compounds with a high Ni load. Several reactions with reduced Ni content showed that only a reaction with a 1:0.5:13 U:Ni:Bi molar ratio resulted in a HfCuSi_2_-type phase UNi_0.13_Bi_2_.

**Figure 1:**
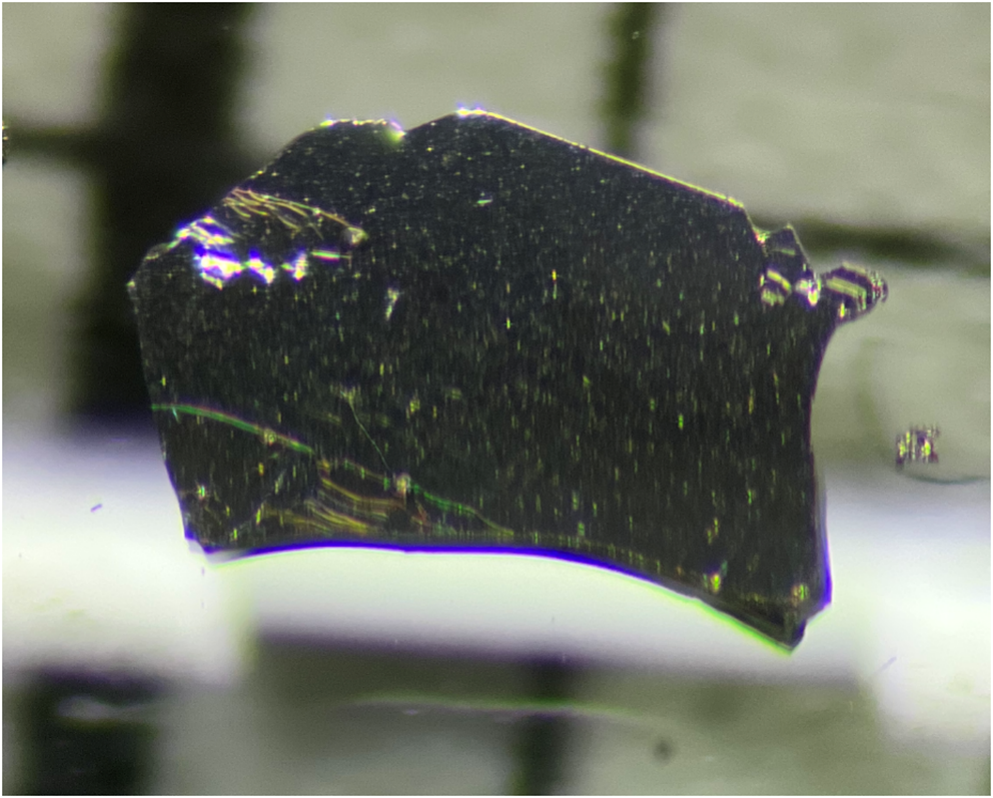
Image of a UCo_0.47_Bi_2_ single crystal on a millimeter paper. The crystal was obtained from a 1:3:13 U:Co:Bi flux reaction.

In an attempt to determine the boundary limits of the transition metal site occupancy, we investigated the effects of altering the U:Co:Bi ratios. By changing the initial Co content in flux reactions (Table S1), we obtained UCo_*x*_Bi_2_ crystals with a composition varying within a stability range of 0.15 ≤ *x* ≤ 0.47. Using arc melting as a synthetic method proved ineffective for samples containing Co, and it mainly yielded the binary UBi_2_ phase as the main product. However, we successfully synthesized arc-melted UNi_*x*_Bi_2_ samples with Ni content *x* varying between 0.1 and 0.3 (Figures S1–S3). A slightly narrower stability range for the UCo_*x*_Bi_2_ system vs UCu_*x*_Bi_2_ can be attributed to the transition metal formal oxidation state (+2 and +1 for Co and Cu, respectively) and size variations, which affect the electron filling of the system.^20^

### Structure Description

We determined the structures of UCo_*x*_Bi_2_ and UNi_*x*_Bi_2_ using single crystal X-ray diffraction.^28,29^ All samples crystallize in the HfCuSi_2_ structure type and *P*4/*nmm* space group (Tables S2–S21). Free refinement of the *M* site occupancies resulted in a range of 0.15–0.47 for the UCo_*x*_Bi_2_ system (Figures S4–S6). In the U–Ni–Bi system, only UNi_0.13_Bi_2_ single crystals were identified (Figure S7), as a further increase of the Ni fraction in the starting reaction led to the formation of a competing phase U_3_Ni_3_Bi_4_. SEM EDS was utilized as a method for the verification of compositions determined via the refinement of SCXRD data.^30^ The compositional determinations based on the data obtained from SEM EDS (Tables S22–S28 and Figures S8–S60) and SCXRD (Table S2) are in good agreement, from which the Co content was determined to vary in a 0.15–0.47 range. SEM EDS data were not obtained for the Ni analog due to rapid sample deterioration.

Despite significantly differing compositions, varying transition metal site deficiency in all studied UCo_*x*_Bi_2_ crystals does not change their structure, all of which crystallize in the HfCuSi_2_ structure type (Figure 2). As a general description, each unit cell contains a single square net sublayer comprised of Bi atoms. Between these two sublayers lie two additional corrugated square net sublayers, which are comprised of U and Bi. Between these two nonplanar sublayers lies another sublayer consisting of partially occupied transition metal sites. We found that the subtle structural differences between U-containing series are correlated with the identity of the incorporated transition metal. For example, this correlation is readily observed upon a comparison of bond lengths (Tables S5–S17 and S21) and unit cell parameters in U*M*_*x*_Bi_2_ (M = Co, Ni, Cu) phases. A direct comparison is sometimes challenging due to the deficient nature of the transition metal site. However, one can still compare phases with similar transition metal contents. This correlation was observed upon a comparison of the Bi2–Bi2 bond lengths that make up the square net sublayer, 3.1507(8) Å (*x* = 0.145) for Co, 3.1555(7) Å (*x* = 0.13) for Ni, and 3.1688(4) Å (*x* = 0.20) for Cu, and the unit cell volumes 178.18(13) Å^3^ (*x* = 0.145) for Co, 179.13(9) Å^3^ (*x* = 0.13) for Ni, and 182.17(6) Å^3^ (*x* = 0.20) for Cu. These values are in good correlation with those observed in the U*M*_*x*_Sb_2_ analogs, with square net Sb–Sb bond lengths of 3.0406 Å (*x* = 0.46) for Co, 3.0540(7) Å (*x* = 0.5) for Ni, and 3.0610(6) Å (*x* = 0.44) for Cu, and unit cell volumes of 165.63 Å^3^ (*x* = 0.46) for Co, 168.16(7) Å^3^ (*x* = 0.5) for Ni, and 171.95(7) Å^3^ (*x* = 0.44) for Cu.^31–33^ The same correlation observed across analogs is indicative of a trend, in which the bond lengths that make up the square net sublayer and overall unit cell volumes increase from *R*Co_*x*_ *Pn*_2_ to *R*Ni_*x*_ *Pn*_2_ to *R*Cu_*x*_ *Pn*_2_ when the transition metal content, *x*, is comparable. Furthermore, the increase in unit cell volume from Co to Cu is also observed in other structure types, such as Y_3_Au_3_Sb_4_, CaBe_2_Ge_2_, CeNiSb_3_, and Y_2_HfS_5_, regardless of the presence or absence of both square net subunits and site deficiency.^34–38^

**Figure 2:**
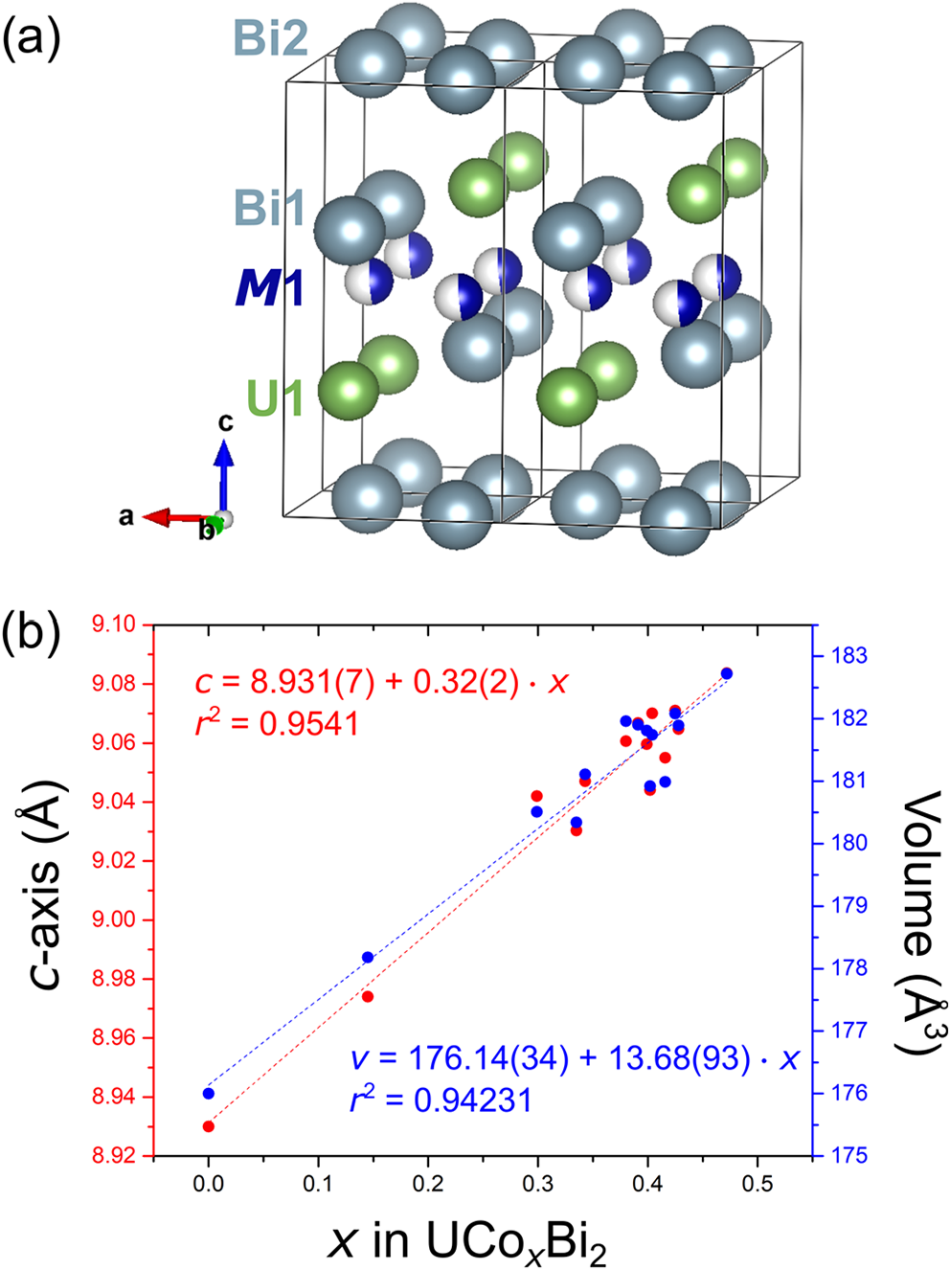
(a) View of the structure of U*M*_*x*_Bi_2_ structure where M = Co or Ni. (b) Unit cell parameters, length of the *c*-axis and volume, as a function of Co content obtained from single crystal XRD data.

As it has been previously observed in the analogous UCu_*x*_Bi_2_ series,^20^ the crystallographic parameters of the obtained UCo_*x*_Bi_2_ phases are in direct correlation with the transition metal content. The effect the Co content has on the corresponding unit cell parameters is demonstrated in Figure 2. As *x* increases from 0.15 to 0.47 in the Co system, the *c*-axis was observed to increase from 8.974(4) Å to 9.0836(9) Å and the unit cell volume from 178.18(13) Å^3^ to 182.72(3) Å^3^.

Like that of the Cu series,^20^ the Co and Ni analogs were found to contain shorter intralayer, square net (Bi2–Bi2) bonds than interlayer (Bi2–Bi1 and Bi2–U1) bonds. The Bi2–Bi2, Bi2–Bi1, and Bi2–U1 bonds determined from the SCXRD data were found to range from 3.1507(8) Å to 3.1714(2) Å, 3.9114(14) Å to 3.9220(13) Å, and 3.3547(11) Å to 3.3784(13) Å, respectively, for the Co series, and to be 3.1555(7) Å, 3.9156(13) Å, and 3.3572(10) Å, respectively, for the Ni compound. As such, the new UCo_*x*_Bi_2_ and UNi_*x*_Bi_2_ analogs exhibit a tolerance factor *t* < 0.95 (*t* = *d*_sq_/*d*_nn_, where *d*_sq_ is the distance between the Bi atoms in the square nets, and *d*_nn_ is the distance between a Bi atom in the square net and the closest atom from outside the net), thus satisfying the criterion proposed by Schoop et al. for quasi-isolated square nets with Dirac crossings.^13,15,39^

### DFT Calculations of the Structure Stability

In our previous work,^20^ we developed a computational approach^40–45^ for modeling and predicting the stability ranges of transition metal deficient compounds that showed successful results for the UCu_*x*_Bi_2_ and UCu_*x*_Sb_2_ series. To further probe the applicability of this approach to the stability in M-site deficient HfCuSi_2_ type compounds, we employed this approach for the Co and Ni systems (Tables S29–S52 and Figure 3).

**Figure 3:**
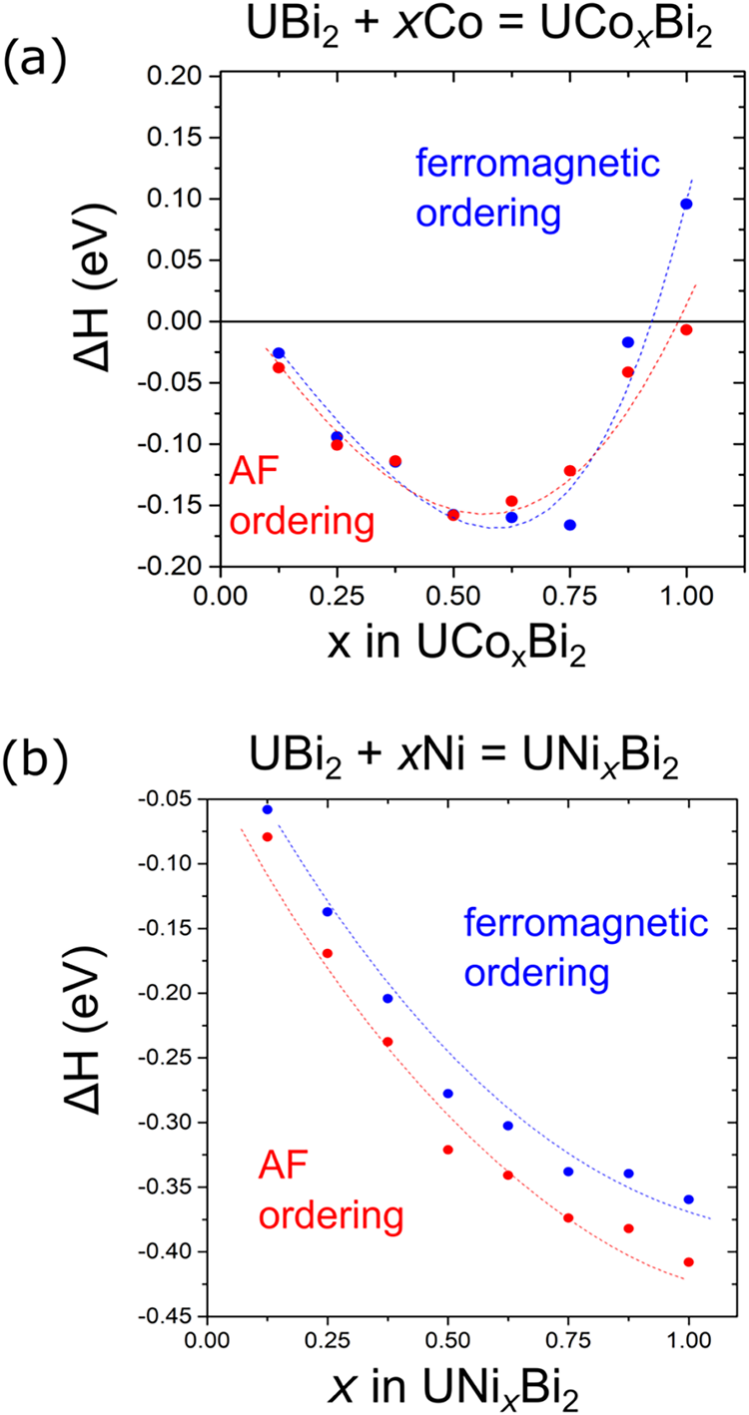
DFT optimization of UCo_*x*_Bi_2_ and UNi_*x*_Bi_2_ phases. Calculated enthalpies of reaction corresponding to the formation of (a) UCo_*x*_Bi_2_ and (b) UNi_*x*_Bi_2_ from UBi_2_ and the corresponding transition metal for *x* = 0.125 to 1. Blue and red circles correspond to the energies of optimized structures with ferro- and antiferromagnetic ordering. The dashed lines serve as guides for the eye, following the overall trend in the reaction enthalpy change.

The computationally predicted stability range for the Co series (Figure 3a) is in a satisfactory agreement with that which was observed experimentally. DFT calculations predicted the most stable composition to be that in which the Co content is ≈ 0.25 to 0.75, in which the higher end of the range is favored when the system is ferromagnetically ordered and the lower when it is ordered antiferromagnetically. The DFT stability range is shifted toward a higher Co content compared to the experimentally observed maximum value of *x* ≈ 0.5, even in the presence of an extreme excess of Co. The calculations also indicate the ability to obtain lower Co contents that are less stable, which was experimentally supported by the increased rate at which they were observed to oxidize in the presence of air. The calculations show slightly more stable antiferromagnetic ordering in the systems with lower Co contents, which agree well with the experimental observations (vide infra).

Unraveling thermodynamic stabilities in the Ni series proved to be more challenging than that of Co, highlighting the importance of taking into account the competing phases for an accurate stability range prediction. When considering the formation of the target UNi_*x*_Bi_2_ compounds alone, the computational method predicted the Ni series to be increasingly stable up to a stoichiometric UNiBi_2_ composition (Figure 3b). However, experimentally observed phases in this work are only those that contain low Ni contents, with *x* = 0.1–0.3. Higher Ni concentrations in the starting reaction led to the formation of a competing ternary phase U_3_Ni_3_Bi_4_ (Tables S53–S56 and Figure S61). Additional formation energy calculations of U_3_Ni_3_Bi_4_, from the equation U_3_Ni_3_Bi_4_ + 2Bi = 3UNiBi_2_, showed that this phase is 0.337 eV more stable than the stoichiometric UNiBi_2_ phase. Overall, our computational studies show that the experimental data support the model’s ability to predict the stability range of transition metal-site deficient compounds accurately. However, they also highlight the importance of taking competing phases into account by building a full convex hull diagram.^46^

The notable discrepancy between the calculated and experimentally observed stability ranges is most likely a result of thermodynamic and kinetic limitations experienced as a result of the synthetic methods implemented.^3,47^ For example, though the stability range of the UNi_*x*_Bi_2_ system experimentally observed in this work is limited to 0 ≤ *x* ≤ 0.3, different synthetic techniques, such as those employed by Kaczorowski,^48^ have seemingly broadened this range. Many earlier experiments with these HfCuSi_2_-type phases were performed before the realization that they have the ability to accommodate varying amounts of transition metal. As a result, these earlier studies often report these phases as being stoichiometric, 1:1:2, when they could in fact be site-deficient. The stoichiometric UNiBi_2_ phase reported in Kaczorowski’s study on several ternary HfCuSi_2_-type uranium pnictides^48^ highlights the effect of the synthetic technique on the obtainable transition metal range. Based on a comparison of the previously reported unit cell, *a* = *b* = 4.470 Å *c* = 9.073 Å,^48^ to those calculated in this work from the DFT optimized UNi_*x*_Bi_2_ phases (Tables S37–S52), the previously reported phase most likely contains a ∼0.375 Ni content, whose calculated unit cell is *a* = *b* = 4.514 Å *c* = 9.073 Å. The higher Ni content, *x* > 0.3, is further supported by a comparison of the reported and experimental Néel temperatures, which are discussed in detail in the Magnetic properties section. This specific discrepancy in obtainable Ni-content is attributed to the synthetic differences which involve the prereacting of the U*Pn*_2_ binary, which is used as reagent along with the transition metal, and a temperature profile which occurs at notably lower temperatures, ≤ 700 °C.^48^

### Magnetic Properties

To reveal the effect of transition metal site deficiency on the resulting material’s magnetic properties, magnetic susceptibility data were collected for a single crystal and powder sample of the UCo_*x*_Bi_2_ phases, in which *x* = 0.3 and 0.4, respectively. For the single crystal UCo_0.3_Bi_2_ sample, measurements were collected with the external magnetic field (*H*) applied parallel to the easy axis (*c*), indicated as (*H*∥*c*), as well as perpendicular to the easy axis, indicated as (*H*⊥*c*). Magnetic susceptibility data revealed an antiferromagnetic ordering at the Néel temperature of 83 K. Inverse susceptibility plots for the *x* = 0.3 samples with *H*∥*c* and *H*⊥*c* are linear in the regions >180 and >160 K, respectively, which served as a basis for Curie–Weiss law fittings.^49–51^ The fittings show magnetic moments of 3.18 and 3.55 μ_B_ per formula unit and the Weiss constants of 54.3 and −155.9 K, respectively. The average effective magnetic moment calculated as μ_av_ = (μ_||_+2 μ_⊥_)/3 is 3.43 μ_B_, which is below the calculated value of 3.62 μ_B_/U^3+^. The discrepancies between experimental and calculated effective magnetic moments of these HfCuSi_2_-type phases are likely due to the partial itinerant nature of the uranium electrons. Although the nonzero Weiss constants can be indicative of competing ferro-and antiferromagnetic interactions between the U atoms, they are also likely due to crystal electric field (CEF) effects,^49,50^ as was observed in the previously reported related systems, e.g., UCu_*x*_Bi_2_ and UAu_0.8_Bi_2_,^20,48,52^ which can also contribute to effective magnetic moment deviation from the calculated value. Magnetization vs applied magnetic field, *M*v*H*, data (Figure 4c) revealed a typical linear behavior characteristic of antiferromagnetic ordering for UCo_0.3_Bi_2_.^50,51^

**Figure 4:**
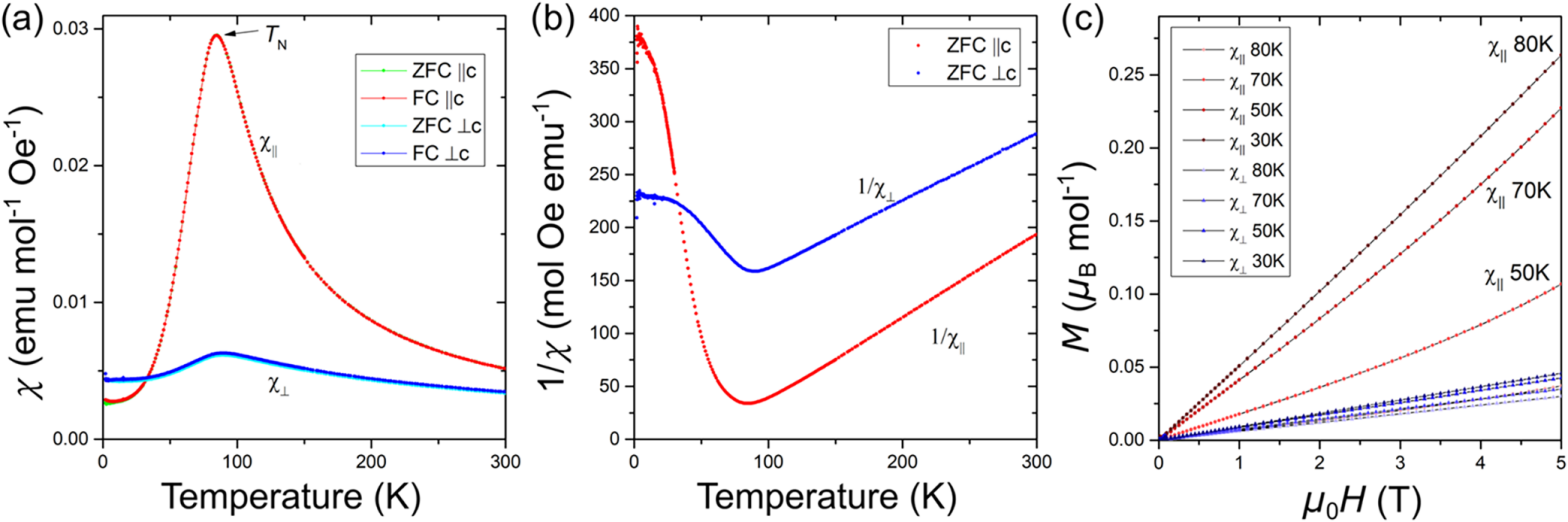
Magnetism of UCo_0.3_Bi_2_ single crystals. (a) DC molar magnetic susceptibility (χ_mol_) vs temperature. (b) Inverse magnetic susceptibility (χ_mol_^–1^) vs temperature. (c) Magnetization as a function of applied magnetic field at 30, 50, 70, and 80 K.

For the UCo_0.4_Bi_2_ phase, a powder sample obtained by grinding single crystals was used to collect magnetic susceptibility data because none of the crystals were large enough in mass to be measured individually. The magnetic susceptibility plot of the UCo_0.4_Bi_2_ phase (Figure 5) shows an antiferromagnetic transition at *T*_N_ = 69 K, indicating a variation of the ordering temperature due to the difference in the composition. Unlike the UCo_0.3_Bi_2_ single crystal data, the powder UCo_0.4_Bi_2_ sample features a second transition with a ferromagnetic component at a temperature slightly below 50 K. The presence of a ferromagnetic component at low temperatures was additionally confirmed by the presence of a small hysteresis loop on the magnetization vs magnetic field plot (Figure S62). Although secondary magnetic ordering transitions sometimes occur in similar compositions, and even small changes in the transition metal content can lead to switching the magnetic ordering in the system,^23,52^ we could not completely exclude the potential presence of a ferromagnetic impurity in the sample. Since magnetic susceptibility is very sensitive to such impurities, they can remain undetected in PXRD patterns and neutron diffraction experiments are necessary for an unambiguous confirmation of the second transition. The inverse susceptibility plot for the powder UCo_0.4_Bi_2_ sample is linear in the region >100 K, which served as a basis for Curie–Weiss law fittings. The fitting showed a magnetic moment of 2.74 μ_B_ per formula unit and a Weiss constant of 26.9 K. Although the derived effective magnetic moment is lower than the calculated one, 3.62 μ_B_/U^3+^, this is common for other HfCuSi_2_-type U-containing compounds and, again, are likely due to crystal electric field (CEF) effects.^25,31,32,52,53^

**Figure 5:**
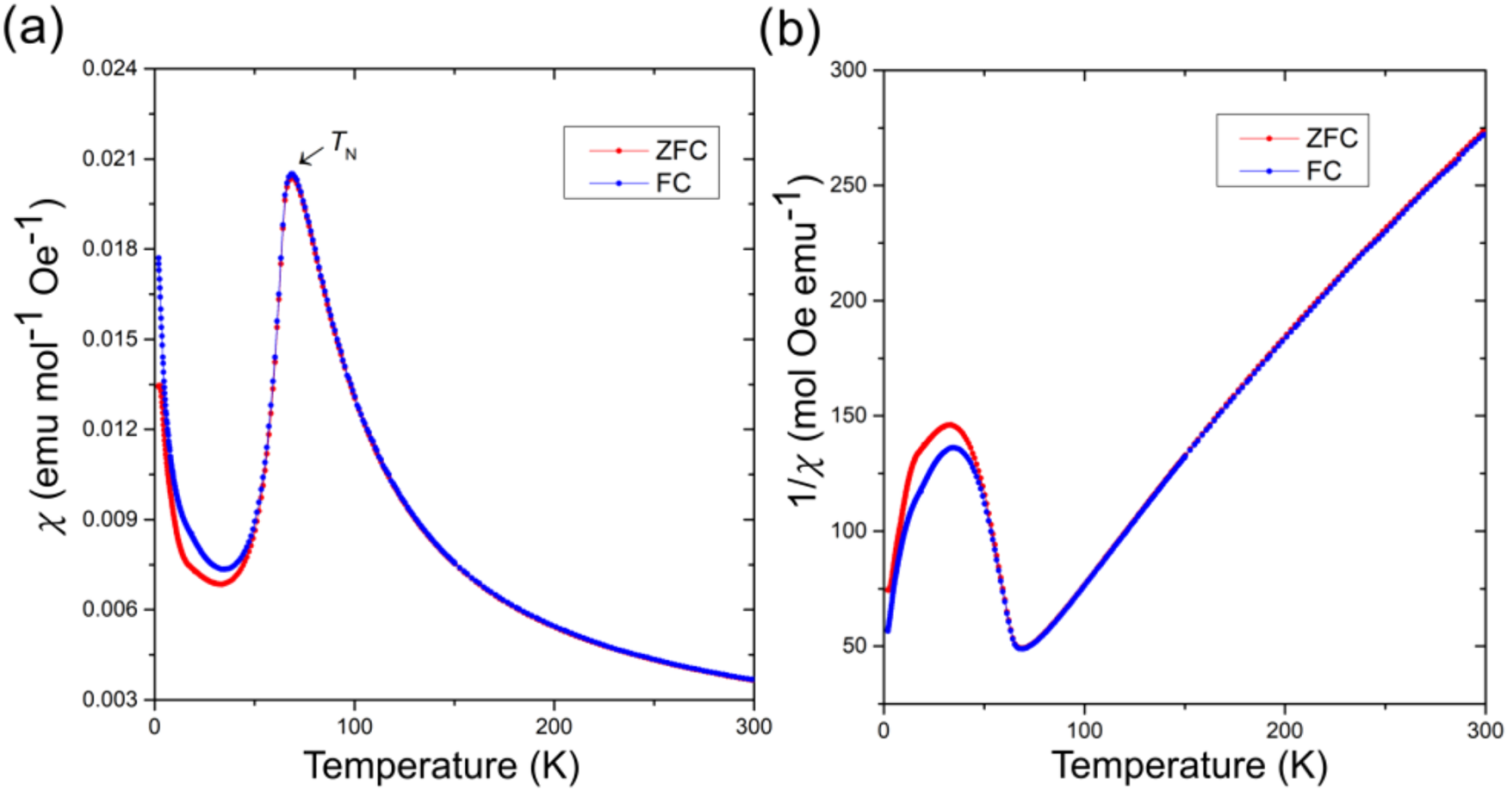
Magnetism of powder UCo_0.4_Bi_2_. (a) DC molar magnetic susceptibility (χ_mol_) vs temperature (b) inverse magnetic susceptibility (χ_mol_^–1^) vs temperature.

We collected magnetic susceptibility data for powder samples of the UNi_*x*_Bi_2_ phases with *x* = 0.1 and 0.2. A temperature-independent correction, χ_0_, was applied to the data and plotted as a function of temperature according to the equation: $\chi -\chi_{0}=\frac{C}{T}$ where *C* is the Curie constant and *T* is temperature. The corrected susceptibility and inverse susceptibility plots for both samples (Figure 6) are similar to those observed for the Co analog, in which an antiferromagnetic transition is observed at high temperature followed by a weak ferromagnetic transition at low temperatures. The Néel temperatures were determined to be 181.1 and 176.5 K for the UNi_*x*_Bi_2_ phases in which *x* = 0.1 and 0.2, respectively. Inverse susceptibility plots for the *x* = 0.1 and 0.2 samples are linear in the regions >200 K, which served as a basis for Curie–Weiss law fittings. The fittings show magnetic moments of 3.84 and 3.35 μ_B_ per formula unit with Weiss constants of −75.6 K and −44.6 K, respectively.

**Figure 6:**
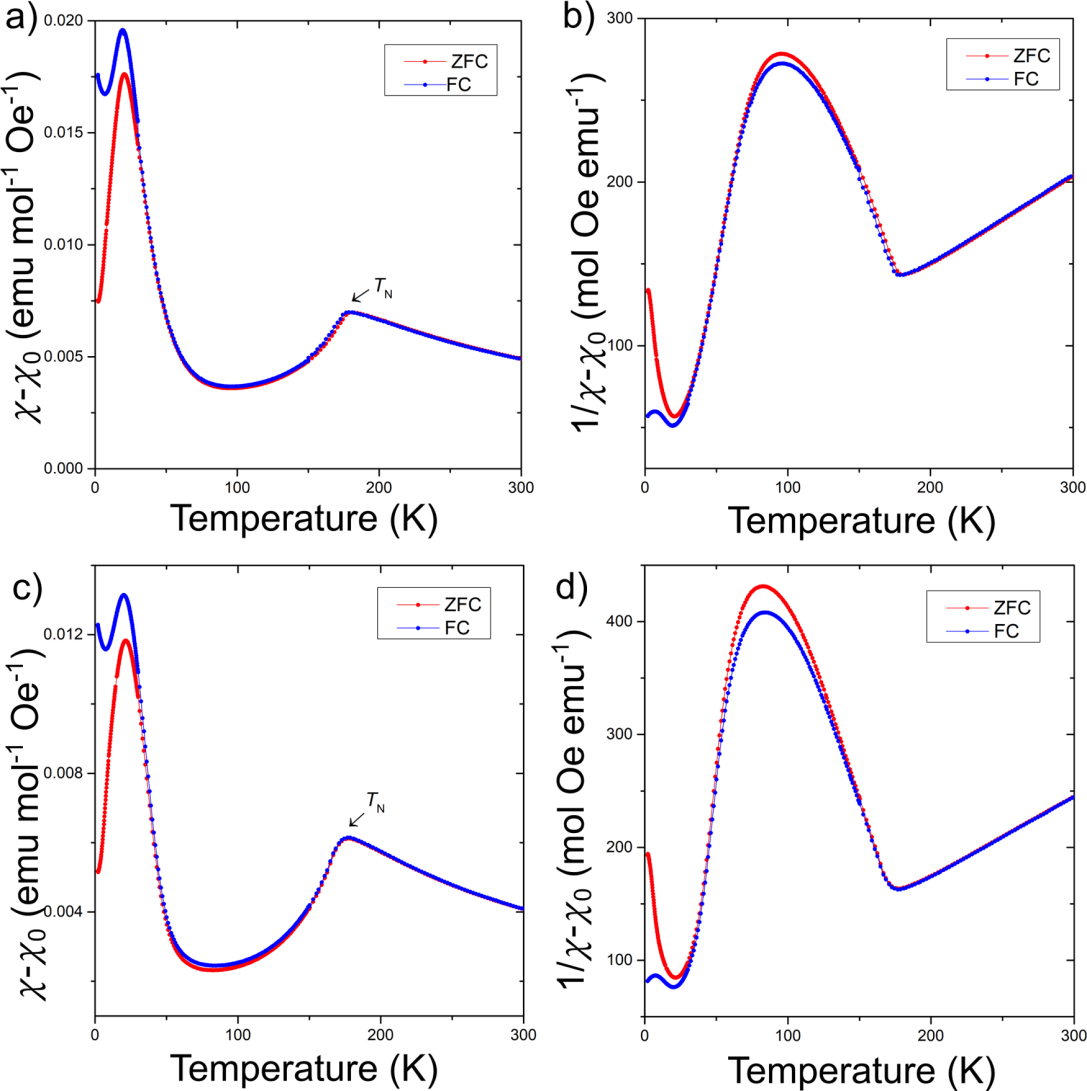
Magnetism of powder UNi_0.1_Bi_2_ (a and b) and UNi_0.2_Bi_2_ (c and d) samples with an applied temperature-independent correction, χ_0_. (a and c) DC molar magnetic susceptibility (χ_mol_) vs temperature (b and d) inverse magnetic susceptibility (χ_mol_^–1^) vs temperature.

Like that of the UCo_0.4_Bi_2_ phase, the MvH plots of both Ni phases, UNi_0.1_Bi_2_ and UNi_0.2_Bi_2_, (Figures S63 and S64a–d) display small hystereses indicative of weak ferromagnetic contributions, which are most likely a result of the same ferromagnetic impurity encountered with the Cu analog.^20^ The presence of a potential common impurity between these two compounds suggests it does not include a transition metal. However, to the best of our knowledge, there has been no report of a uranium bismuthide or its oxide with a ferromagnetic transition that occurs at a temperature around 20–25 K. Therefore, the attribution of the low-temperature ferromagnetic transition to an impurity remains ambiguous until its nature is identified.

It was experimentally observed that both the extent of incorporation and identity of the transition metal affect magnetic properties.^20,23,54^ Since, in most cases of site-deficient HfCuSi_2_-type compounds, the magnetic moments on the uranium atoms within the same *ab* plane order ferromagnetically, and the ordering along the *c*-axis between the layers is antiferromagnetic, the Néel temperature (*T*_N_) can be employed as a measure of antiferromagnetic interactions between the U layers.^54,55^ As anticipated, the subsequent decrease in Néel temperatures upon increase in transition metal content is indicative of weakening antiferromagnetic interactions as the *M* content is increased and the *c*-axis is elongated. This trend is likely a result of an increased separation of the magnetic atoms, uranium in this case, from one another as more transition metal atoms are incorporated into the layer between. The experimentally determined Néel temperatures for both systems differ from that of UBi_2_, reported to be 183 K,^56,57^ which supports the successful incorporation of transition metals. Interestingly, the incorporation of the magnetic Co and Ni does not alter the overall magnetic ordering. However, the role of the transition metals cannot be reduced to that of just magnetic spacers, as the Néel temperatures depend on their nature. For example, previously reported UCu_0.4_Bi_2_ and UCu_0.3_Bi_2_ order at different *T*_N_ (92 and 118 K, respectively)^20^ compared to their UCo_0.4_Bi_2_ and UCo_0.3_Bi_2_ counterparts (69 and 83 K). This change cannot just be attributed to a larger unit cell, even though the *c*-axis lengths differ significantly between the two systems, 9.22 and 9.15 Å for UCu_0.4_Bi_2_ and UCu_0.3_Bi_2_ vs 9.06 and 9.03 Å for UCo_0.4_Bi_2_ and UCo_0.3_Bi_2_. This indicates that the nature of the transition metal plays a significant role in defining the magnetic properties of the resulting phase, enabling transition metal site mixing as an additional lever to tune magnetic properties in these phases.

## Conclusion

We have synthesized two new uranium-based HfCuSi_2_-type series, UCo_*x*_Bi_2_ and UNi_*x*_Bi_2_ and characterized their structural and magnetic properties to investigate the effects of transition metal site occupancy. Single-crystal X-ray diffraction confirmed that all compositions crystallize in the tetragonal *P*4*/nmm* space group and HfCuSi_2_ structure type. The comparison of bond lengths and unit cell parameters across U*M*_*x*_Bi_2_ (M = Co, Ni, Cu) and their corresponding antimonide analogs revealed a consistent increase in both unit cell volume and Bi–Bi bond lengths from Co to Cu, a trend corroborated by DFT-optimized structural models. Computational investigations adequately confirm the experimentally observed compositional ranges and ground state ordering, reaffirming the model’s capability to guide the synthesis of transition-metal-deficient systems. In the Ni system, the calculations further emphasized the necessity of accounting for competing phases, such as U_3_Ni_3_Bi_4_, to fully describe phase stability. Magnetization measurements revealed that both systems exhibit antiferromagnetic ordering across all compositions, with the Néel temperature varying as a function of transition metal nature and content and demonstrating a direct relationship between transition metal occupancy and the weakening of exchange interactions along the *c*-axis. These findings highlight the potential for controlled tuning of the structural and magnetic properties in square-net-based topological materials through deliberate manipulation of transition metal site occupancy and mixing, paving the way for further exploration of correlated electron behavior in actinide-containing systems.

## Experimental Methods

**Caution.** Although the uranium precursor used in this synthesis contains depleted uranium, it is required that proper procedures for handling radioactive materials are observed. All handling of radioactive materials was performed in laboratories specially designated for the study of radioactive actinide materials.

**Caution.** Uranium metal, some target phases and side products, such as UBi_2_, are highly pyrophoric and prone to spontaneous ignition in air. Small quantities of the samples should be handled at one time in inert atmosphere.

### Reagents

Bi (Unique Metals, 99.99%), Co (Bean Town Chemical, 99.5%), Ni (STREM Chemicals Inc., 99.99%), HNO_3_ (VWR Chemicals, 68–70%), and acetone (Fisher Chemical, 99.5%) were used as received. U sheet (Manufacturing Sciences Corporation, >99%) was cleaned of the oxide layer using concentrated nitric acid (HNO_3_) followed by an acetone rinse and cut into smaller pieces prior to use in a reaction.

### Synthesis

Single crystals of UCo_*x*_Bi_2_ and UNi_*x*_Bi_2_ were grown using the self-flux method with excess bismuth serving as the flux. Uranium, cobalt or nickel, and bismuth were combined in a 1:2:13 or 1:0.5:13 molar ratio, respectively, loaded into an alumina crucible (9 and 6 mm outer and inner diameters, respectively). The crucible was loaded into a quartz tube (12 and 10.5 mm outer and inner diameter, 20 cm long) and covered by a piece of silica wool for product filtration. The silica tube was vacuum sealed and placed into a programmable box furnace. The sealed reaction was heated to 950 °C over a period of 2 h, where it dwelled for 2 h before being cooled to 480 °C over a period of 3 h. Upon cooling to 480 °C, the reaction was immediately centrifuged. The product was manually recovered upon opening the reaction vessels in an argon-filled glovebox.

Powder samples were synthesized via arc melting and annealing. The reagents were ground into a powdered form in an argon-filled glovebox where they were mixed in exact stoichiometric ratios for the desired product, with a 2% excess of Bi to account for its volatility under high temperatures. Each mixture was then placed into a die and pressed into a pellet using a hand press inside the glovebox. These pellets were then individually removed and immediately placed into the chamber of the arc melter, where they were vacuumed down and placed under an argon atmosphere. Each pellet was arc melted two times, once on each side, to ensure thorough mixing with the sample being flipped and placed in a new, clean well between each melt. Each resulting sample was immediately transferred to the glovebox upon its removal from the arc melter, where it was encased in tantalum foil and placed in a silica tube. The silica tube was vacuum sealed and placed into a programmable box furnace where it was annealed at 800 °C for a period of 12 h. The annealed samples were transferred back to the glovebox where they were opened and the products were manually retrieved from the tantalum foil.

### Magnetism

Magnetic property measurements were performed using a Quantum Design MPMS 3 SQUID magnetometer. Zero-field-cooled (ZFC) magnetic susceptibility measurements from powder samples were performed from 2 to 300 K in an applied field of 0.1 T. The raw powder data was corrected for radial offset and sample shape effects according to the method described by Morrison and zur Loye.^58^ Single crystal data was collected in the same temperature range from a single crystal that was oriented and glued to a quartz paddle using GE-7031 varnish. Crystal orientation was establish using a PXRD scan of a plate-like single crystal on a zero-background Si slide.

### Calculations

First-principles calculations were performed using density functional theory (DFT) with the Vienna Ab-initio Package (VASP) planewave code,^40,41^ generalized gradient approximation of Perdew, Burke and Ernzerhof (PBE),^42^ and projector augmented wave (PAW) method.^43,44^ For all Ni-containing phases, the ground state geometries at 0 K were optimized by relaxing the cell volume, atomic positions, and cell symmetry until the maximum force on each atom is less than 0.001 eV/Å. For the ferromagnetically ordered Co-containing phases, the ground state geometries at 0 K were optimized by relaxing the cell volume, atomic positions, and cell symmetry until the change in total energy between two ionic steps is less than 0.001 eV/Å. For the antiferromagnetically ordered Co-containing phases, the ground state geometries at 0 K were set to match those of their ferromagnetic counterparts with corresponding compositions. Spin-polarized calculations were performed, with 520 eV cutoff energy for the plane wave basis set, 10^–6^ eV energy convergence criteria, and 6 × 6 × 5 *k*-point meshes for the quadrupled unit cells generated by VASPKIT package.

### Powder X-ray Diffraction

Powder X-ray diffraction (PXRD) data for phase identification and phase purity confirmation were collected on polycrystalline samples. Data were collected on a Bruker D2 PHASER diffractometer utilizing Cu Kα radiation. The data were collected over the range from 10° to 65° 2θ with a step size of 0.02°. The PXRD patterns are shown in Figures S1–S3.

### Crystal Structure

Single-crystal X-ray diffraction data was collected at 300(2) K on a Bruker D8 QUEST diffractometer equipped with an Incoatec IμS 3.0 microfocus radiation source (Mo Kα, λ = 0.71073 Å) and a PHOTON II area detector. The crystals were mounted on a microloop using immersion oil. The raw data reduction and absorption corrections were performed using APEX3 v2019–1.0 and SADABS programs.^59,60^ Initial structure solutions were obtained with SHELXS-2017 using direct methods and Olex2 GUI.^61^ Full matrix least-squares refinements against F^2^ were performed with SHELXL software.^62^ The crystallographic data and results of the diffraction experiments are summarized in Tables S2–S21 and S53–S56.

### Scanning Electron Microscopy (SEM)

SEM images were acquired using a Thermo Fisher Teneo FE-SEM operated at 20 kV with a CBS detector.

## Supplementary Material



## Notes

Additional experimental details, materials, and methods, crystallographic parameters, SEM EDS data including spectra and compositional mapping, PXRD patterns, DFT optimized structures and parameters, and additional magnetic property measurements (DOCX)
