# Supplementary material for: Compositional Tuning of Magnetic Properties in a Series of Transition Metal Site-Deficient UCo x Bi2 and UNi x Bi2 Phases
Source: Inorg Chem. 2026 Jan 3;65(2):1611–20. doi: 10.1021/acs.inorgchem.5c05232 (PMC12820931; doi:10.1021/acs.inorgchem.5c05232)
Supplement: Supplementary file 1 [file ic5c05232_si_001.docx]

**Supporting Information for**

**Compositional tuning of magnetic properties in a series of transition metal site-deficient UCo*_x_*Bi_2_ and UNi*_x_*Bi_2_ phases**

*Hope A. Long,^a^ Hope E. Smith,^b^ Gregory Morrison,^c^ Vladislav V. Klepov^a^**

^a^ Department of Chemistry, University of Georgia, Athens, Georgia, 30602, United States

^b^ Department of Chemistry and Biochemistry, University of South Carolina, Columbia, South Carolina 29208, United States

^c^ Center for Hierarchical Waste Form Materials, and Department of Chemistry and Biochemistry, University of South Carolina, Columbia, South Carolina 29208, United States

**Table of contents**

| **Figures S1-S3.** PXRD patterns of UNi*_x_*Bi_2_ phases | S3 |
| --- | --- |
| **Table S1.** Flux reaction summary table | S6 |
| **Tables S2-S21.** Crystallographic parameters of UCo*_x_*Bi_2_ and UNi_0.13_Bi_2_ phases | S6 |
| **Figures S4-S6.** Thermal ellipsoids of UCo*_x_*Bi_2_ phases | S20 |
| **Figure S7.** Thermal ellipsoids of UNi_0.13_Bi_2_ | S22 |
| **Tables S22-S28 and Figures S8-S59.** EDS data | S23 |
| **Figure S60.** EDS compositional mapping of a UCo*_x_*Bi_2_ single crystal | S53 |
| **Tables S29-S36.** DFT optimized structure parameters of UCo*_x_*Bi_2_ phases | S54 |
| **Tables S37-S52.** DFT optimized structure parameters of UNi*_x_*Bi_2_ phases | S62 |
| **Tables S53-S56.** Crystallographic parameters of U_3_Ni_3_Bi_4_ | S78 |
| **Figure S61.** Crystal structure of U_3_Ni_3_Bi_4_ | S80 |
| **Figure S62.** MvH plot of a UCo_0.4_Bi_2_ powder sample | S81 |
| **Figures S63 and S64**. Magnetization of powder samples of UNi*_x_*Bi_2_ (*x* = 0.1 and 0.2) | S81 |
| **References** | S83 |





Figure S1. PXRD pattern of UNi_0.1_Bi_2_ powder obtained from arc melting and annealing at 800°C. The broad reflection in the ~13-23° region is due to Kapton tape covering the sample.





Figure S2. PXRD pattern of UNi_0.2_Bi_2_ powder obtained from arc melting and annealing at 800°C. The broad reflection in the ~13-23° region is due to Kapton tape covering the sample.





Figure S3. PXRD pattern of UNi_0.3_Bi_2_ powder obtained from arc melting and annealing at 800°C. The broad reflection in the ~13-23° region is due to Kapton tape covering the sample.

Table S1. Results of flux reactions, in terms of Co content in resulting single crystals as determined by SC XRD, by reaction ratio of Co in initial reaction

| Co molar ratio in reaction (*X* in 1:*X*:13 U:Co:Bi) | Co content in crystal product (*x* in UCo*x*Bi2) |
| --- | --- |
|  |  |
| 0.5 | 0.145 |
| 1 | 0.299-0.343 |
| 1.5 | 0.380- 0.428 |
| 2 | 0.399-0.416 |
| 3 | 0.472 |

Table S2. Crystallographic data for UCo*_x_*Bi_2_ single crystals (*x* = 0.145 – 0.472)

| Empirical formula | 0.145 | 0.299 | 0.335 | 0.343 | 0.380 | 0.391 | 0.399 | 0.402 | 0.404 | 0.416 | 0.425 | 0.428 | 0.472 |
| --- | --- | --- | --- | --- | --- | --- | --- | --- | --- | --- | --- | --- | --- |
| Formula weight | 664.53 | 673.67 | 676.32 | 676.32 | 678.38 | 678.97 | 679.56 | 679.56 | 679.86 | 680.45 | 681.04 | 681.33 | 683.69 |
| Temperature/K | 297 | 299 | 299 | 299 | 299 | 299 | 300 | 300 | 300 | 300 | 299 | 299 | 300 |
| Crystal system | tetragonal | | | | | | | | | | | | |
| Space group | P4/nmm | | | | | | | | | | | | |
| a/Å | 4.4558(12) | 4.4681(9) | 4.4689(6) | 4.4742(10) | 4.4813(4) | 4.4791(3) | 4.4797(4) | 4.4727(8) | 4.4764(11) | 4.4708(11) | 4.4801(13) | 4.4795(4) | 4.4850(3) |
| b/Å | 4.4558(12) | 4.4681(9) | 4.4689(6) | 4.4742(10) | 4.4813(4) | 4.4791(3) | 4.4797(4) | 4.4727(8) | 4.4764(11) | 4.4708(11) | 4.4801(13) | 4.4795(4) | 4.4850(3) |
| c/Å | 8.974(4) | 9.042(2) | 9.0303(18) | 9.047(3) | 9.0606(14) | 9.0668(8) | 9.0596(13) | 9.044(3) | 9.070(3) | 9.055(3) | 9.071(4) | 9.0647(8) | 9.0836(9) |
| α/° | 90 | | | | | | | | | | | | |
| β/° | 90 | | | | | | | | | | | | |
| γ/° | 90 | | | | | | | | | | | | |
| Volume/Å^3^ | 178.18(13) | 180.51(8) | 180.34(6) | 181.11(10) | 181.96(4) | 181.90(3) | 181.81(4) | 180.92(8) | 181.74(11) | 180.99(11) | 182.08(13) | 181.89(3) | 182.72(3) |
| Z | 2 | | | | | | | | | | | | |
| ρcalcg/cm^3^ | 12.387 | 12.394 | 12.455 | 12.402 | 12.382 | 12.396 | 12.414 | 12.474 | 12.423 | 12.486 | 12.422 | 12.44 | 12.427 |
| μ/mm^‑1^ | 144.219 | 143.04 | 143.374 | 142.769 | 142.259 | 142.345 | 142.464 | 143.162 | 142.536 | 143.171 | 142.362 | 142.529 | 142.060 |
| F(000) | 524 | 532 | 535 | 535 | 537 | 537 | 538 | 538 | 538 | 538 | 539 | 539 | 541 |
| Crystal size/mm^3^ | 0.05 × 0.03 × 0.03 | 0.04 × 0.04 × 0.02 | 0.04 × 0.04 × 0.02 | 0.04 × 0.04 × 0.02 | 0.04 × 0.04 × 0.02 | 0.06 × 0.06 × 0.04 | 0.04 × 0.04 × 0.01 | 0.06 × 0.05 × 0.04 | 0.04 × 0.04 × 0.01 | 0.04 × 0.04 × 0.01 | 0.04 × 0.04 × 0.02 | 0.04 × 0.04 × 0.02 | 0.04 × 0.04 × 0.01 |
| Radiation | MoKα (λ = 0.71073) | | | | | | | | | | | | |
| 2Θ range for data collection/° | 9.084 to 54.522 | 4.504 to 54.93 | 9.028 to 66.232 | 4.502 to 54.872 | 4.496 to 54.912 | 4.492 to 54.924 | 4.496 to 66.05 | 4.504 to 54.892 | 4.49 to 66.098 | 4.498 to 49.928 | 4.49 to 54.906 | 4.494 to 54.924 | 4.484 to 49.934 |
| Index ranges | -4 ≤ h ≤ 5 | -5 ≤ h ≤ 5 | -6 ≤ h ≤ 4 | -5 ≤ h ≤ 5 | -5 ≤ h ≤ 5 | -5 ≤ h ≤ 5 | -6 ≤ h ≤ 6 | -5 ≤ h ≤ 5 | -4 ≤ h ≤ 6 | -5 ≤ h ≤ 5 | -5 ≤ h ≤ 5 | -5 ≤ h ≤ 5 | -5 ≤ h ≤ 5 |
|  | -5 ≤ k ≤ 4 | -5 ≤ k ≤ 5 | -6 ≤ k ≤ 5 | -5 ≤ k ≤ 5 | -5 ≤ k ≤ 5 | -5 ≤ k ≤ 5 | -6 ≤ k ≤ 6 | -5 ≤ k ≤ 5 | -6 ≤ k ≤ 5 | -5 ≤ k ≤ 4 | -5 ≤ k ≤ 5 | -5 ≤ k ≤ 5 | -4 ≤ k ≤ 5 |
|  | -11 ≤ l ≤ 9 | -11 ≤ l ≤ 11 | -13 ≤ l ≤ 11 | -11 ≤ l ≤ 11 | -9 ≤ l ≤ 11 | -11 ≤ l ≤ 11 | -12 ≤ l ≤ 13 | -11 ≤ l ≤ 11 | -13 ≤ l ≤ 13 | -10 ≤ l ≤ 10 | -10 ≤ l ≤ 11 | -9 ≤ l ≤ 11 | -10 ≤ l ≤ 10 |
| Reflections collected | 811 | 1680 | 2036 | 2401 | 1691 | 3159 | 2223 | 3368 | 1935 | 1306 | 1580 | 1607 | 1887 |
| Independent reflections | 149 | 153 | 238 | 153 | 155 | 155 | 240 | 153 | 239 | 121 | 152 | 155 | 122 |
|  | R_int_ = 0.0479 | R_int_ = 0.0526 | R_int_ = 0.0700 | R_int_ = 0.0594 | R_int_ = 0.0587 | R_int_ = 0.0696 | R_int_ = 0.0723 | R_int_ = 0.0631 | R_int_ = 0.0611 | R_int_ = 0.0606 | R_int_ = 0.0589 | R_int_ = 0.0651 | R_int_ = 0.0575 |
|  | R_sigma_ = 0.0375 | R_sigma_ = 0.0250 | R_sigma_ = 0.0392 | R_sigma_ = 0.0236 | R_sigma_ = 0.0271 | R_sigma_ = 0.0249 | R_sigma_ = 0.0374 | R_sigma_ = 0.0209 | R_sigma_ = 0.0345 | R_sigma_ = 0.0311 | R_sigma_ = 0.0301 | R_sigma_ = 0.0340 | R_sigma_ = 0.0237 |
| Data/restraints/ parameters | 149/0/13 | 153/0/13 | 238/0/13 | 153/0/13 | 155/0/13 | 155/0/13 | 240/0/13 | 153/0/13 | 239/0/13 | 121/0/13 | 152/0/13 | 155/0/13 | 122/0/13 |
| Goodness-of-fit on F2 | 1.095 | 1.142 | 1.082 | 1.23 | 1.197 | 1.2 | 1.094 | 1.243 | 1.178 | 1.242 | 1.193 | 1.246 | 1.100 |
| Final R indexes [I>=2σ (I)] | R_1_ = 0.0180 | R_1_ = 0.0201 | R_1_ = 0.0486 | R_1_ = 0.0194 | R_1_ = 0.0208 | R_1_ = 0.0329 | R_1_ = 0.0262 | R_1_ = 0.0197 | R_1_ = 0.0294 | R_1_ = 0.0354 | R_1_ = 0.0267 | R_1_ = 0.0326 | R_1_ = 0.0400 |
|  | wR_2_ = 0.0385 | wR_2_ = 0.0443 | wR_2_ = 0.1160 | wR_2_ = 0.0431 | wR_2_ = 0.0483 | wR_2_ = 0.0886 | wR_2_ = 0.0571 | wR_2_ = 0.0545 | wR_2_ = 0.0811 | wR_2_ = 0.0867 | wR_2_ = 0.0634 | wR_2_ = 0.0669 | wR_2_ = 0.1098 |
| Final R indexes [all data] | R_1_ = 0.0213 | R_1_ = 0.0210 | R_1_ = 0.0531 | R_1_ = 0.0202 | R_1_ = 0.0221 | R_1_ = 0.0332 | R_1_ = 0.0278 | R_1_ = 0.0200 | R_1_ = 0.0324 | R_1_ = 0.0355 | R_1_ = 0.0279 | R_1_ = 0.0331 | R_1_ = 0.0400 |
|  | wR_2_ = 0.0394 | wR_2_ = 0.0446 | wR_2_ = 0.1186 | wR_2_ = 0.0434 | wR_2_ = 0.0488 | wR_2_ = 0.0892 | wR_2_ = 0.0579 | wR_2_ = 0.0547 | wR_2_ = 0.0824 | wR_2_ = 0.0871 | wR_2_ = 0.0652 | wR_2_ = 0.0672 | wR_2_ = 0.1098 |
| Largest diff. peak/hole / e Å-3 | 1.92/-2.07 | 2.27/-2.35 | 4.53/-6.53 | 2.65/-2.51 | 3.30/-2.03 | 4.54/-3.13 | 3.15/-1.81 | 3.06/-1.94 | 5.32/-4.13 | 4.04/-1.95 | 4.39/-1.68 | 3.54/-3.28 | 4.11/-3.71 |
| CCDC | 2500824 | 2500825 | 2500826 | 2500827 | 2500829 | 2500822 | 2500832 | 2500823 | 2500831 | 2500833 | 2500828 | 2500830 | 2500821 |

Table S3. Fractional Atomic Coordinates (×10^4^) and Equivalent Isotropic Displacement Parameters (Å^2^×10^3^) for UCo*_x_*Bi_2_ (*x* = 0.145 – 0.472). U_eq_ is defined as1/3 of the trace of the orthogonalized U_IJ_ tensor.

| Atom | Empirical Formula | 0.145 | 0.299 | 0.335 | 0.343 | 0.380 | 0.391 | 0.399 | 0.402 | 0.404 | 0.416 | 0.425 | 0.428 | 0.472 |
| --- | --- | --- | --- | --- | --- | --- | --- | --- | --- | --- | --- | --- | --- | --- |
| U1 | x | 7500 | 7500 | 7500 | 7500 | 7500 | 7500 | 7500 | 7500 | 7500 | 7500 | 7500 | 7500 | 7500 |
|  | y | 7500 | 7500 | 7500 | 7500 | 7500 | 7500 | 7500 | 7500 | 7500 | 7500 | 7500 | 7500 | 7500 |
|  | z | 2794.7(10) | 2785.3(9) | 2783.7(13) | 2784.6(9) | 2784.6(9) | 2784.0(14) | 2784.3(6) | 2783.9(9) | 2785.6(9) | 2784.0(14) | 2784.8(10) | 2785.4(10) | 2781.7(19) |
|  | U(eq) | 15.6(3) | 14.6(3) | 17.4(4) | 14.7(3) | 12.0(3) | 11.1(5) | 14.2(2) | 12.6(3) | 10.3(3) | 11.9(7) | 12.3(4) | 13.6(4) | 9.2(10) |
| Bi1 | x | 2500 | 2500 | 2500 | 2500 | 2500 | 2500 | 2500 | 2500 | 2500 | 2500 | 2500 | 2500 | 2500 |
|  | y | 2500 | 2500 | 2500 | 2500 | 2500 | 2500 | 2500 | 2500 | 2500 | 2500 | 2500 | 2500 | 2500 |
|  | z | 3584.8(10) | 3561.5(9) | 3557.1(12) | 3552.4(9) | 3543.4(9) | 3547.1(13) | 3541.5(6) | 3550.6(8) | 3540.9(9) | 3544.7(13) | 3544.4(10) | 3543.0(9) | 3542.3(16) |
|  | U(eq) | 17.8(3) | 18.0(3) | 21.1(4) | 18.2(3) | 15.9(3) | 15.0(6) | 18.3(2) | 16.2(3) | 14.3(3) | 16.0(7) | 16.0(4) | 17.4(4) | 13.2(10) |
| Bi2 | x | 7500 | 7500 | 7500 | 7500 | 7500 | 7500 | 7500 | 7500 | 7500 | 7500 | 7500 | 7500 | 7500 |
|  | y | 2500 | 2500 | 2500 | 2500 | 2500 | 2500 | 2500 | 2500 | 2500 | 2500 | 2500 | 2500 | 2500 |
|  | z | 0 | 0 | 0 | 0 | 0 | 0 | 0 | 0 | 0 | 0 | 0 | 0 | 0 |
|  | U(eq) | 15.7(3) | 14.0(3) | 16.9(4) | 14.1(3) | 11.4(3) | 10.6(5) | 13.8(2) | 12.0(3) | 9.8(3) | 11.8(7) | 12.1(4) | 13.1(4) | 9.4(10) |
| Co1 | x | 2500 | 2500 | 2500 | 2500 | 2500 | 2500 | 2500 | 2500 | 2500 | 2500 | 2500 | 2500 | 2500 |
|  | y | 7500 | 7500 | 7500 | 7500 | 7500 | 7500 | 7500 | 7500 | 7500 | 7500 | 7500 | 7500 | 7500 |
|  | z | 5000 | 5000 | 5000 | 5000 | 5000 | 5000 | 5000 | 5000 | 5000 | 5000 | 5000 | 5000 | 5000 |
|  | U(eq) | 15(8) | 15(3) | 22(4) | 17(3) | 15(3) | 18(4) | 16.7(16) | 25(3) | 13(2) | 26(4) | 21(3) | 22(3) | 27(4) |

Table S4. Anisotropic Displacement Parameters (Å^2^×10^3^) for UCo*_x_*Bi_2_ (*x* = 0.145-0.472). The Anisotropic displacement factor exponent takes the form: -2π^2^[h^2^a*^2^U_11_+2hka*b*U_12_+…].

| Atom | Empirical Formula | 0.145 | 0.299 | 0.335 | 0.343 | 0.380 | 0.391 | 0.399 | 0.402 | 0.404 | 0.416 | 0.425 | 0.428 | 0.472 |
| --- | --- | --- | --- | --- | --- | --- | --- | --- | --- | --- | --- | --- | --- | --- |
| U1 | U_11_ | 15.0(4) | 14.9(4) | 18.0(5) | 15.7(4) | 13.6(4) | 13.6(6) | 15.3(3) | 14.3(4) | 9.7(3) | 12.9(8) | 14.4(5) | 17.0(5) | 10.8(10) |
|  | U_22_ | 15.0(4) | 14.9(4) | 18.0(5) | 15.7(4) | 13.6(4) | 13.6(6) | 15.3(3) | 14.3(4) | 9.7(3) | 12.9(8) | 14.4(5) | 17.0(5) | 10.8(10) |
|  | U_33_ | 16.9(5) | 13.9(5) | 16.1(6) | 12.5(5) | 8.8(4) | 6.2(7) | 12.0(4) | 9.2(5) | 11.3(5) | 10.0(11) | 7.9(6) | 7.0(5) | 6.1(14) |
| Bi1 | U_11_ | 18.0(4) | 18.7(4) | 22.2(5) | 19.8(4) | 18.0(4) | 17.8(6) | 19.5(3) | 18.3(4) | 14.2(3) | 17.2(8) | 18.7(5) | 21.4(5) | 14.7(10) |
|  | U_22_ | 18.0(4) | 18.7(4) | 22.2(5) | 19.8(4) | 18.0(4) | 17.8(6) | 19.5(3) | 18.3(4) | 14.2(3) | 17.2(8) | 18.7(5) | 21.4(5) | 14.7(10) |
|  | U_33_ | 17.4(5) | 16.6(5) | 19.0(7) | 15.2(5) | 11.8(5) | 9.3(8) | 15.9(4) | 11.8(5) | 14.5(5) | 13.5(12) | 10.7(6) | 9.4(6) | 10.2(15) |
| Bi2 | U_11_ | 15.3(3) | 13.8(3) | 17.1(5) | 14.5(3) | 12.8(4) | 12.9(6) | 14.3(2) | 13.4(4) | 9.0(3) | 13.0(8) | 13.8(5) | 16.2(5) | 11.1(10) |
|  | U_22_ | 15.3(3) | 13.8(3) | 17.1(5) | 14.5(3) | 12.8(4) | 12.9(6) | 14.3(2) | 13.4(4) | 9.0(3) | 13.0(8) | 13.8(5) | 16.2(5) | 11.1(10) |
|  | U_33_ | 16.6(5) | 14.4(5) | 16.4(7) | 13.2(5) | 8.8(5) | 6.0(8) | 12.9(4) | 9.3(5) | 11.3(5) | 9.5(10) | 8.6(6) | 6.9(6) | 5.9(13) |
| Co1 | U_11_ | 16(9) | 20(4) | 25(5) | 21(4) | 20(4) | 26(6) | 20(2) | 31(4) | 20(3) | 33(5) | 25(4) | 29(4) | 37(6) |
|  | U_22_ | 16(9) | 20(4) | 25(5) | 21(4) | 20(4) | 26(6) | 20(2) | 31(4) | 20(3) | 33(5) | 25(4) | 29(4) | 37(6) |
|  | U_33_ | 14(14) | 6(5) | 16(6) | 9(5) | 6(4) | 3(6) | 11(3) | 11(4) | 1(3) | 12(7) | 13(5) | 8(4) | 5(7) |

Table S5. Bond lengths for HL25E_fin. (UCo_0.145_Bi_2_)

| **Atom** | **Atom** | **Length/Å** |  | **Atom** | **Atom** | **Length/Å** |
| --- | --- | --- | --- | --- | --- | --- |
| U1 | Bi1 | 3.2295(9) |  | U1 | Co1^2^ | 2.9800(9) |
| U1 | Bi1^1^ | 3.249(2) |  | U1 | Co1 | 2.9800(9) |
| U1 | Bi1^2^ | 3.2295(9) |  | Bi1 | Co1^8^ | 2.5645(8) |
| U1 | Bi1^3^ | 3.2295(9) |  | Bi1 | Co1^1^ | 2.5645(8) |
| U1 | Bi1^4^ | 3.2295(9) |  | Bi1 | Co1^9^ | 2.5645(8) |
| U1 | Bi2^5^ | 3.3547(12) |  | Bi1 | Co1 | 2.5645(8) |
| U1 | Bi2^6^ | 3.3547(11) |  | Bi2 | Bi2^5^ | 3.1507(8) |
| U1 | Bi2 | 3.3547(11) |  | Bi2 | Bi2^10^ | 3.1507(8) |
| U1 | Co1^7^ | 2.9800(9) |  | Bi2 | Bi2^11^ | 3.1507(8) |
| U1 | Co1^1^ | 2.9800(9) |  | Bi2 | Bi2^6^ | 3.1507(8) |
|  |  |  |  |  |  |  |
| ^1^1-X,1-Y,1-Z; ^2^1+X,+Y,+Z; ^3^+X,1+Y,+Z; ^4^1+X,1+Y,+Z; ^5^2-X,1-Y,-Z; ^6^1-X,1-Y,-Z; ^7^1-X,2-Y,1-Z; ^8^-X,1-Y,1-Z; ^9^+X,-1+Y,+Z; ^10^2-X,-Y,-Z; ^11^1-X,-Y,2-Z | | | | | | |

Table S6. Bond Lengths for HL32F_1_fin. (UCo_0.299_Bi_2_)

| **Atom** | **Atom** | **Length/Å** |  | **Atom** | **Atom** | **Length/Å** |
| --- | --- | --- | --- | --- | --- | --- |
| U1 | Bi1^1^ | 3.2364(6) |  | U1 | Co1^4^ | 3.0002(7) |
| U1 | Bi1^2^ | 3.3033(14) |  | U1 | Co1 | 3.0002(7) |
| U1 | Bi1 | 3.2364(6) |  | Bi1 | Co1^7^ | 2.5851(6) |
| U1 | Bi1^3^ | 3.2364(6) |  | Bi1 | Co1^2^ | 2.5851(6) |
| U1 | Bi1^4^ | 3.2364(6) |  | Bi1 | Co1^8^ | 2.5851(6) |
| U1 | Bi2^3^ | 3.3665(8) |  | Bi1 | Co1 | 2.5851(6) |
| U1 | Bi2^5^ | 3.3665(8) |  | Bi2 | Bi2^9^ | 3.1594(6) |
| U1 | Bi2 | 3.3665(8) |  | Bi2 | Bi2^10^ | 3.1594(6) |
| U1 | Co1^6^ | 3.0002(7) |  | Bi2 | Bi2^11^ | 3.1594(6) |
| U1 | Co1^2^ | 3.0002(7) |  | Bi2 | Bi2^5^ | 3.1594(6) |
|  |  |  |  |  |  |  |
| ^1^1-X,1-Y,1-Z; ^2^1+X,+Y,+Z; ^3^+X,1+Y,+Z; ^4^1+X,1+Y,+Z; ^5^2-X,1-Y,-Z; ^6^1-X,1-Y,-Z; ^7^1-X,2-Y,1-Z; ^8^-X,1-Y,1-Z; ^9^+X,-1+Y,+Z; ^10^2-X,-Y,-Z; ^11^1-X,-Y,2-Z | | | | | | |

Table S7. Bond Lengths for HL32F_2_fin. (UCo_0.335_Bi_2_)

| **Atom** | **Atom** | **Length/Å** |  | **Atom** | **Atom** | **Length/Å** |
| --- | --- | --- | --- | --- | --- | --- |
| U1 | Bi1^1^ | 3.2362(6) |  | U1 | Co1^4^ | 2.9997(8) |
| U1 | Bi1^2^ | 3.3045(16) |  | U1 | Co1 | 2.9997(8) |
| U1 | Bi1 | 3.2362(6) |  | Bi1 | Co1^7^ | 2.5866(6) |
| U1 | Bi1^3^ | 3.2362(6) |  | Bi1 | Co1^2^ | 2.5866(6) |
| U1 | Bi1^4^ | 3.2362(6) |  | Bi1 | Co1^8^ | 2.5866(6) |
| U1 | Bi2^3^ | 3.3633(10) |  | Bi1 | Co1 | 2.5866(6) |
| U1 | Bi2^5^ | 3.3633(9) |  | Bi2 | Bi2^9^ | 3.1600(4) |
| U1 | Bi2 | 3.3633(9) |  | Bi2 | Bi2^10^ | 3.1600(4) |
| U1 | Co1^6^ | 2.9997(8) |  | Bi2 | Bi2^11^ | 3.1600(4) |
| U1 | Co1^2^ | 2.9997(8) |  | Bi2 | Bi2^5^ | 3.1600(4) |
|  |  |  |  |  |  |  |
| ^1^1-X,1-Y,1-Z; ^2^1+X,+Y,+Z; ^3^+X,1+Y,+Z; ^4^1+X,1+Y,+Z; ^5^2-X,1-Y,-Z; ^6^1-X,1-Y,-Z; ^7^1-X,2-Y,1-Z; ^8^-X,1-Y,1-Z; ^9^+X,-1+Y,+Z; ^10^2-X,-Y,-Z; ^11^1-X,-Y,2-Z | | | | | | |

Table S8. Bond Lengths for HL32F_3_fin. (UCo_0.343_Bi_2_)

| **Atom** | **Atom** | **Length/Å** |  | **Atom** | **Atom** | **Length/Å** |
| --- | --- | --- | --- | --- | --- | --- |
| U1 | Bi1^1^ | 3.2391(7) |  | U1 | Co1^3^ | 3.0036(8) |
| U1 | Bi1^2^ | 3.3140(16) |  | U1 | Co1 | 3.0036(8) |
| U1 | Bi1^3^ | 3.2391(7) |  | Bi1 | Co1^8^ | 2.5923(6) |
| U1 | Bi1^4^ | 3.2391(7) |  | Bi1 | Co1^2^ | 2.5923(6) |
| U1 | Bi1 | 3.2391(7) |  | Bi1 | Co1^9^ | 2.5923(6) |
| U1 | Bi2^5^ | 3.3691(9) |  | Bi1 | Co1 | 2.5923(6) |
| U1 | Bi2^6^ | 3.3692(9) |  | Bi2 | Bi2^5^ | 3.1637(7) |
| U1 | Bi2 | 3.3692(9) |  | Bi2 | Bi2^10^ | 3.1637(7) |
| U1 | Co1^7^ | 3.0036(8) |  | Bi2 | Bi2^11^ | 3.1637(7) |
| U1 | Co1^2^ | 3.0036(8) |  | Bi2 | Bi2^6^ | 3.1637(7) |
|  |  |  |  |  |  |  |
| ^1^1-X,1-Y,1-Z; ^2^1+X,+Y,+Z; ^3^+X,1+Y,+Z; ^4^1+X,1+Y,+Z; ^5^2-X,1-Y,-Z; ^6^1-X,1-Y,-Z; ^7^1-X,2-Y,1-Z; ^8^-X,1-Y,1-Z; ^9^+X,-1+Y,+Z; ^10^2-X,-Y,-Z; ^11^1-X,-Y,2-Z | | | | | | |

Table S9. Bond Lengths for HL32G_2_fin. (UCo_0.380_Bi_2_)

| **Atom** | **Atom** | **Length/Å** |  | **Atom** | **Atom** | **Length/Å** |
| --- | --- | --- | --- | --- | --- | --- |
| U1 | Bi1^1^ | 3.2425(4) |  | U1 | Co1^3^ | 3.0083(6) |
| U1 | Bi1^2^ | 3.3271(12) |  | U1 | Co1 | 3.0083(6) |
| U1 | Bi1^3^ | 3.2425(4) |  | Bi1 | Co1^8^ | 2.6005(5) |
| U1 | Bi1^4^ | 3.2425(4) |  | Bi1 | Co1^2^ | 2.6005(5) |
| U1 | Bi1 | 3.2425(4) |  | Bi1 | Co1^9^ | 2.6005(5) |
| U1 | Bi2^5^ | 3.3743(7) |  | Bi1 | Co1 | 2.6005(5) |
| U1 | Bi2^6^ | 3.3743(7) |  | Bi2 | Bi2^5^ | 3.1688(3) |
| U1 | Bi2 | 3.3743(7) |  | Bi2 | Bi2^10^ | 3.1688(3) |
| U1 | Co1^7^ | 3.0083(6) |  | Bi2 | Bi2^11^ | 3.1688(3) |
| U1 | Co1^2^ | 3.0083(6) |  | Bi2 | Bi2^6^ | 3.1688(3) |
|  |  |  |  |  |  |  |
| ^1^1-X,1-Y,1-Z; ^2^1+X,+Y,+Z; ^3^+X,1+Y,+Z; ^4^1+X,1+Y,+Z; ^5^2-X,1-Y,-Z; ^6^1-X,1-Y,-Z; ^7^1-X,2-Y,1-Z; ^8^-X,1-Y,1-Z; ^9^+X,-1+Y,+Z; ^10^2-X,-Y,-Z; ^11^1-X,-Y,2-Z | | | | | | |

Table S10. Bond Lengths for HL25B_1. (UCo_0.391_Bi_2_)

| **Atom** | **Atom** | **Length/Å** |  | **Atom** | **Atom** | **Length/Å** |
| --- | --- | --- | --- | --- | --- | --- |
| U1 | Bi1^1^ | 3.2378(6) |  | U1 | Co1^2^ | 3.0030(7) |
| U1 | Bi1 | 3.2378(6) |  | U1 | Co1^3^ | 3.0030(7) |
| U1 | Bi1^2^ | 3.2378(6) |  | Bi1 | Co1^7^ | 2.5922(5) |
| U1 | Bi1^3^ | 3.3150(14) |  | Bi1 | Co1^8^ | 2.5922(5) |
| U1 | Bi1^4^ | 3.2378(6) |  | Bi1 | Co1^3^ | 2.5922(5) |
| U1 | Bi2^5^ | 3.3675(8) |  | Bi1 | Co1 | 2.5922(5) |
| U1 | Bi2 | 3.3675(8) |  | Bi2 | Bi2^9^ | 3.1627(6) |
| U1 | Bi2^4^ | 3.3675(8) |  | Bi2 | Bi2^10^ | 3.1627(6) |
| U1 | Co1^6^ | 3.0030(7) |  | Bi2 | Bi2^5^ | 3.1627(6) |
| U1 | Co1 | 3.0030(7) |  | Bi2 | Bi2^11^ | 3.1627(6) |
|  |  |  |  |  |  |  |
| ^1^1-X,1-Y,1-Z; ^2^1+X,+Y,+Z; ^3^+X,1+Y,+Z; ^4^1+X,1+Y,+Z; ^5^2-X,1-Y,-Z; ^6^1-X,1-Y,-Z; ^7^1-X,2-Y,1-Z; ^8^-X,1-Y,1-Z; ^9^+X,-1+Y,+Z; ^10^2-X,-Y,-Z; ^11^1-X,-Y,2-Z | | | | | | |

Table S11. Bond Lengths for HL32I_2_fin. (UCo_0.399_Bi_2_)

| **Atom** | **Atom** | **Length/Å** |  | **Atom** | **Atom** | **Length/Å** |
| --- | --- | --- | --- | --- | --- | --- |
| U1 | Bi1^1^ | 3.2411(3) |  | U1 | Co1^2^ | 3.0077(5) |
| U1 | Bi1 | 3.2411(3) |  | U1 | Co1^3^ | 3.0077(5) |
| U1 | Bi1^2^ | 3.2411(3) |  | Bi1 | Co1^7^ | 2.6006(4) |
| U1 | Bi1^3^ | 3.3287(9) |  | Bi1 | Co1^8^ | 2.6006(4) |
| U1 | Bi1^4^ | 3.2411(3) |  | Bi1 | Co1^3^ | 2.6006(4) |
| U1 | Bi2^5^ | 3.3734(5) |  | Bi1 | Co1 | 2.6006(4) |
| U1 | Bi2 | 3.3734(5) |  | Bi2 | Bi2^9^ | 3.1676(3) |
| U1 | Bi2^4^ | 3.3734(5) |  | Bi2 | Bi2^10^ | 3.1676(3) |
| U1 | Co1^6^ | 3.0077(5) |  | Bi2 | Bi2^5^ | 3.1676(3) |
| U1 | Co1 | 3.0077(5) |  | Bi2 | Bi2^11^ | 3.1676(3) |
|  |  |  |  |  |  |  |
| ^1^1-X,1-Y,1-Z; ^2^1+X,+Y,+Z; ^3^+X,1+Y,+Z; ^4^1+X,1+Y,+Z; ^5^2-X,1-Y,-Z; ^6^1-X,1-Y,-Z; ^7^1-X,2-Y,1-Z; ^8^-X,1-Y,1-Z; ^9^+X,-1+Y,+Z; ^10^2-X,-Y,-Z; ^11^1-X,-Y,2-Z | | | | | | |

Table S12. Bond Lengths for HL25B_3_fin. (UCo_0.402_Bi_2_)

| **Atom** | **Atom** | **Length/Å** |  | **Atom** | **Atom** | **Length/Å** |
| --- | --- | --- | --- | --- | --- | --- |
| U1 | Bi1^1^ | 3.2378(6) |  | U1 | Co1^2^ | 3.0030(7) |
| U1 | Bi1 | 3.2378(6) |  | U1 | Co1^3^ | 3.0030(7) |
| U1 | Bi1^2^ | 3.2378(6) |  | Bi1 | Co1^7^ | 2.5922(5) |
| U1 | Bi1^3^ | 3.3150(14) |  | Bi1 | Co1^8^ | 2.5922(5) |
| U1 | Bi1^4^ | 3.2378(6) |  | Bi1 | Co1^3^ | 2.5922(5) |
| U1 | Bi2^5^ | 3.3675(8) |  | Bi1 | Co1 | 2.5922(5) |
| U1 | Bi2 | 3.3675(8) |  | Bi2 | Bi2^9^ | 3.1627(6) |
| U1 | Bi2^4^ | 3.3675(8) |  | Bi2 | Bi2^10^ | 3.1627(6) |
| U1 | Co1^6^ | 3.0030(7) |  | Bi2 | Bi2^5^ | 3.1627(6) |
| U1 | Co1 | 3.0030(7) |  | Bi2 | Bi2^11^ | 3.1627(6) |
|  |  |  |  |  |  |  |
| ^1^1-X,1-Y,1-Z; ^2^1+X,+Y,+Z; ^3^+X,1+Y,+Z; ^4^1+X,1+Y,+Z; ^5^2-X,1-Y,-Z; ^6^1-X,1-Y,-Z; ^7^1-X,2-Y,1-Z; ^8^-X,1-Y,1-Z; ^9^+X,-1+Y,+Z; ^10^2-X,-Y,-Z; ^11^1-X,-Y,2-Z | | | | | | |

Table S13. Bond Lengths for HL32I_1_fin. (UCo_0.404_Bi_2_)

| **Atom** | **Atom** | **Length/Å** |  | **Atom** | **Atom** | **Length/Å** |
| --- | --- | --- | --- | --- | --- | --- |
| U1 | Bi1^1^ | 3.2386(8) |  | U1 | Co1^4^ | 3.0072(8) |
| U1 | Bi1^2^ | 3.3318(16) |  | U1 | Co1 | 3.0072(8) |
| U1 | Bi1 | 3.2386(8) |  | Bi1 | Co1^7^ | 2.6002(7) |
| U1 | Bi1^3^ | 3.2386(8) |  | Bi1 | Co1^2^ | 2.6002(7) |
| U1 | Bi1^4^ | 3.2386(8) |  | Bi1 | Co1^8^ | 2.6002(7) |
| U1 | Bi2^3^ | 3.3753(10) |  | Bi1 | Co1 | 2.6002(7) |
| U1 | Bi2^5^ | 3.3753(10) |  | Bi2 | Bi2^9^ | 3.1653(8) |
| U1 | Bi2 | 3.3753(10) |  | Bi2 | Bi2^10^ | 3.1653(8) |
| U1 | Co1^6^ | 3.0072(8) |  | Bi2 | Bi2^11^ | 3.1653(8) |
| U1 | Co1^2^ | 3.0072(8) |  | Bi2 | Bi2^5^ | 3.1653(8) |
|  |  |  |  |  |  |  |
| ^1^1-X,1-Y,1-Z; ^2^1+X,+Y,+Z; ^3^+X,1+Y,+Z; ^4^1+X,1+Y,+Z; ^5^2-X,1-Y,-Z; ^6^1-X,1-Y,-Z; ^7^1-X,2-Y,1-Z; ^8^-X,1-Y,1-Z; ^9^+X,-1+Y,+Z; ^10^2-X,-Y,-Z; ^11^1-X,-Y,2-Z | | | | | | |

Table S14. Bond Lengths for HL32I_3_fin. (UCo_0.416_Bi_2_)

| **Atom** | **Atom** | **Length/Å** |  | **Atom** | **Atom** | **Length/Å** |
| --- | --- | --- | --- | --- | --- | --- |
| U1 | Bi1^1^ | 3.2355(8) |  | U1 | Co1^4^ | 3.0039(11) |
| U1 | Bi1^2^ | 3.3244(18) |  | U1 | Co1 | 3.0039(10) |
| U1 | Bi1 | 3.2355(8) |  | Bi1 | Co1^7^ | 2.5949(8) |
| U1 | Bi1^3^ | 3.2355(8) |  | Bi1 | Co1^2^ | 2.5949(8) |
| U1 | Bi1^4^ | 3.2355(8) |  | Bi1 | Co1^8^ | 2.5949(8) |
| U1 | Bi2^3^ | 3.3693(12) |  | Bi1 | Co1 | 2.5949(8) |
| U1 | Bi2^5^ | 3.3693(12) |  | Bi2 | Bi2^9^ | 3.1613(8) |
| U1 | Bi2 | 3.3693(12) |  | Bi2 | Bi2^10^ | 3.1613(8) |
| U1 | Co1^6^ | 3.0039(10) |  | Bi2 | Bi2^11^ | 3.1613(7) |
| U1 | Co1^2^ | 3.0039(10) |  | Bi2 | Bi2^5^ | 3.1613(7) |
|  |  |  |  |  |  |  |
| ^1^1-X,1-Y,1-Z; ^2^1+X,+Y,+Z; ^3^+X,1+Y,+Z; ^4^1+X,1+Y,+Z; ^5^2-X,1-Y,-Z; ^6^1-X,1-Y,-Z; ^7^1-X,2-Y,1-Z; ^8^-X,1-Y,1-Z; ^9^+X,-1+Y,+Z; ^10^2-X,-Y,-Z; ^11^1-X,-Y,2-Z | | | | | | |

Table S15. Bond Lengths for HL32G_1_fin. (UCo_0.425_Bi_2_)

| **Atom** | **Atom** | **Length/Å** |  | **Atom** | **Atom** | **Length/Å** |
| --- | --- | --- | --- | --- | --- | --- |
| U1 | Bi1^1^ | 3.2420(10) |  | U1 | Co1^4^ | 3.0093(10) |
| U1 | Bi1^2^ | 3.3299(18) |  | U1 | Co1 | 3.0093(10) |
| U1 | Bi1 | 3.2420(10) |  | Bi1 | Co1^7^ | 2.6002(8) |
| U1 | Bi1^3^ | 3.2420(10) |  | Bi1 | Co1^2^ | 2.6002(8) |
| U1 | Bi1^4^ | 3.2420(10) |  | Bi1 | Co1^8^ | 2.6002(8) |
| U1 | Bi2^3^ | 3.3763(11) |  | Bi1 | Co1 | 2.6002(8) |
| U1 | Bi2^5^ | 3.3763(11) |  | Bi2 | Bi2^9^ | 3.1679(9) |
| U1 | Bi2 | 3.3763(11) |  | Bi2 | Bi2^10^ | 3.1679(9) |
| U1 | Co1^6^ | 3.0093(10) |  | Bi2 | Bi2^11^ | 3.1679(9) |
| U1 | Co1^2^ | 3.0093(10) |  | Bi2 | Bi2^5^ | 3.1679(9) |
|  |  |  |  |  |  |  |
| ^1^1-X,1-Y,1-Z; ^2^1+X,+Y,+Z; ^3^+X,1+Y,+Z; ^4^1+X,1+Y,+Z; ^5^2-X,1-Y,-Z; ^6^1-X,1-Y,-Z; ^7^1-X,2-Y,1-Z; ^8^-X,1-Y,1-Z; ^9^+X,-1+Y,+Z; ^10^2-X,-Y,-Z; ^11^1-X,-Y,2-Z | | | | | | |

Table S16. Bond Lengths for HL32G_4_fin. (UCo_0.428_Bi_2_)

| **Atom** | **Atom** | **Length/Å** |  | **Atom** | **Atom** | **Length/Å** |
| --- | --- | --- | --- | --- | --- | --- |
| U1 | Bi1 | 3.2411(4) |  | U1 | Co1^2^ | 3.0077(6) |
| U1 | Bi1^1^ | 3.3281(11) |  | U1 | Co1 | 3.0077(6) |
| U1 | Bi1^2^ | 3.2411(4) |  | Bi1 | Co1^8^ | 2.6001(4) |
| U1 | Bi1^3^ | 3.2411(4) |  | Bi1 | Co1^1^ | 2.6001(4) |
| U1 | Bi1^4^ | 3.2411(4) |  | Bi1 | Co1^9^ | 2.6001(4) |
| U1 | Bi2^5^ | 3.3752(7) |  | Bi1 | Co1 | 2.6001(4) |
| U1 | Bi2^6^ | 3.3752(7) |  | Bi2 | Bi2^5^ | 3.1675(2) |
| U1 | Bi2 | 3.3752(7) |  | Bi2 | Bi2^10^ | 3.1675(3) |
| U1 | Co1^7^ | 3.0077(6) |  | Bi2 | Bi2^11^ | 3.1675(2) |
| U1 | Co1^1^ | 3.0077(6) |  | Bi2 | Bi2^6^ | 3.1675(2) |
|  |  |  |  |  |  |  |
| ^1^1-X,1-Y,1-Z; ^2^1+X,+Y,+Z; ^3^+X,1+Y,+Z; ^4^1+X,1+Y,+Z; ^5^2-X,1-Y,-Z; ^6^1-X,1-Y,-Z; ^7^1-X,2-Y,1-Z; ^8^-X,1-Y,1-Z; ^9^+X,-1+Y,+Z; ^10^2-X,-Y,-Z; ^11^1-X,-Y,2-Z | | | | | | |

Table S17. Bond Lengths for HL7C_fin. (UCo_0.472_Bi_2_)

| **Atom** | **Atom** | **Length/Å** |  | **Atom** | **Atom** | **Length/Å** |
| --- | --- | --- | --- | --- | --- | --- |
| U1 | Bi1 | 3.2458(6) |  | U1 | Co1^4^ | 3.0148(11) |
| U1 | Bi1^1^ | 3.2458(6) |  | U1 | Co1 | 3.0148(11) |
| U1 | Bi1^2^ | 3.339(2) |  | Bi1 | Co1^7^ | 2.6043(7) |
| U1 | Bi1^3^ | 3.2458(6) |  | Bi1 | Co1^2^ | 2.6043(7) |
| U1 | Bi1^4^ | 3.2458(6) |  | Bi1 | Co1^8^ | 2.6043(7) |
| U1 | Bi2^3^ | 3.3784(13) |  | Bi1 | Co1 | 2.6043(7) |
| U1 | Bi2^5^ | 3.3784(13) |  | Bi2 | Bi2^9^ | 3.1714(2) |
| U1 | Bi2 | 3.3784(13) |  | Bi2 | Bi2^10^ | 3.1714(2) |
| U1 | Co1^6^ | 3.0148(11) |  | Bi2 | Bi2^11^ | 3.1714(2) |
| U1 | Co1^2^ | 3.0148(11) |  | Bi2 | Bi2^5^ | 3.1714(2) |
|  |  |  |  |  |  |  |
| ^1^1-X,1-Y,1-Z; ^2^1+X,+Y,+Z; ^3^+X,1+Y,+Z; ^4^1+X,1+Y,+Z; ^5^2-X,1-Y,-Z; ^6^1-X,1-Y,-Z; ^7^1-X,2-Y,1-Z; ^8^-X,1-Y,1-Z; ^9^+X,-1+Y,+Z; ^10^2-X,-Y,-Z; ^11^1-X,-Y,2-Z | | | | | | |

Table S18. Crystallographic data for UNi*_x_*Bi_2_ (*x* = 0.13)

| Empirical formula | 0.13 |
| --- | --- |
| Formula weight | 663.62 |
| Temperature/K | 300 |
| Crystal system | tetragonal |
| Space group | P4/nmm |
| a/Å | 4.4626(9) |
| b/Å | 4.4626(9) |
| c/Å | 8.995(3) |
| α/° | 90 |
| β/° | 90 |
| γ/° | 90 |
| Volume/Å^3^ | 179.13(9) |
| Z | 2 |
| ρcalcg/cm^3^ | 12.304 |
| μ/mm^‑1^ | 143.468 |
| F(000) | 523 |
| Crystal size/mm^3^ | 0.04 × 0.04 × 0.01 |
| Radiation | MoKα (λ = 0.71073) |
| 2Θ range for data collection/° | 9.064 to 66.006 |
| Index ranges | -6 ≤ h ≤ 4 |
|  | -4 ≤ k ≤ 6 |
|  | -13 ≤ l ≤ 13 |
| Reflections collected | 2097 |
| Independent reflections | 234 |
|  | [R_int_ = 0.0574, |
|  | R_sigma_ = 0.0324] |
| Data/restraints/ parameters | 234/0/13 |
| Goodness-of-fit on F2 | 1.076 |
| Final R indexes [I>=2σ (I)] | R_1_ = 0.0322, |
|  | wR_2_ = 0.0685 |
| Final R indexes [all data] | R_1_ = 0.0392, |
|  | wR_2_ = 0.0708 |
| Largest diff. peak/hole / e Å-3 | 2.77/-4.11 |
| CCDC | 2500834 |

Table S19. Fractional Atomic Coordinates (×10^4^) and Equivalent Isotropic Displacement Parameters (Å^2^×10^3^) for UNi*_x_*Bi_2_ (*x* = 0.13). U(eq) is defined as1/3 of the trace of the orthogonalized UIJ tensor.

| **Atom** | ***x*** | ***y*** | ***z*** | **U(eq)** |
| --- | --- | --- | --- | --- |
| U1 | 7500 | 7500 | 2788.8(10) | 11.9(3) |
| Bi2 | 2500 | 7500 | 0 | 12.4(3) |
| Bi1 | 2500 | 2500 | 3577.2(11) | 13.8(3) |
| Ni1 | 2500 | 7500 | 5000 | 7(7) |

Table S20. Anisotropic Displacement Parameters (Å2×103) for UNi*_x_*Bi_2_ (*x* = 0.13). The Anisotropic displacement factor exponent takes the form: -2π2[h2a*2U11+2hka*b*U12+…].

| **Atom** | **U_11_** | **U_22_** | **U_33_** |
| --- | --- | --- | --- |
| U1 | 12.8(3) | 12.8(3) | 10.2(5) |
| Bi2 | 13.2(3) | 13.2(3) | 10.9(5) |
| Bi1 | 14.6(3) | 14.6(3) | 12.1(5) |
| Ni1 | 8(8) | 8(8) | 5(12) |

Table S21. Bond Lengths for HL33D_1_fin. (UNi_0.13_Bi_2_)

| **Atom** | **Atom** | **Length/Å** |  | **Atom** | **Atom** | **Length/Å** |
| --- | --- | --- | --- | --- | --- | --- |
| U1 | Bi2 | 3.3572(10) |  | U1 | Ni1^7^ | 2.9890(8) |
| U1 | Bi2^1^ | 3.3572(10) |  | U1 | Ni1^3^ | 2.9890(8) |
| U1 | Bi2^2^ | 3.3572(10) |  | Bi2 | Bi2^1^ | 3.1555(7) |
| U1 | Bi1^3^ | 3.2342(7) |  | Bi2 | Bi2^8^ | 3.1555(7) |
| U1 | Bi1^4^ | 3.2686(18) |  | Bi2 | Bi2^2^ | 3.1555(7) |
| U1 | Bi1^5^ | 3.2342(7) |  | Bi2 | Bi2^9^ | 3.1555(6) |
| U1 | Bi1 | 3.2342(7) |  | Bi1 | Ni1^4^ | 2.5722(7) |
| U1 | Bi1^6^ | 3.2342(7) |  | Bi1 | Ni1^10^ | 2.5722(7) |
| U1 | Ni1 | 2.9890(8) |  | Bi1 | Ni1 | 2.5722(7) |
| U1 | Ni1^4^ | 2.9890(8) |  | Bi1 | Ni1^11^ | 2.5722(7) |
|  |  |  |  |  |  |  |
| ^1^1-X,2-Y,-Z; ^2^1-X,1-Y,-Z; ^3^1+X,+Y,+Z; ^4^1-X,1-Y,1-Z; ^5^1+X,1+Y,+Z; ^6^+X,1+Y,+Z; ^7^1-X,2-Y,1-Z; ^8^-X,2-Y,-Z; ^9^-X,1-Y,-Z; ^10^+X,-1+Y,+Z; ^11^-X,1-Y,1-Z | | | | | | |

| \| 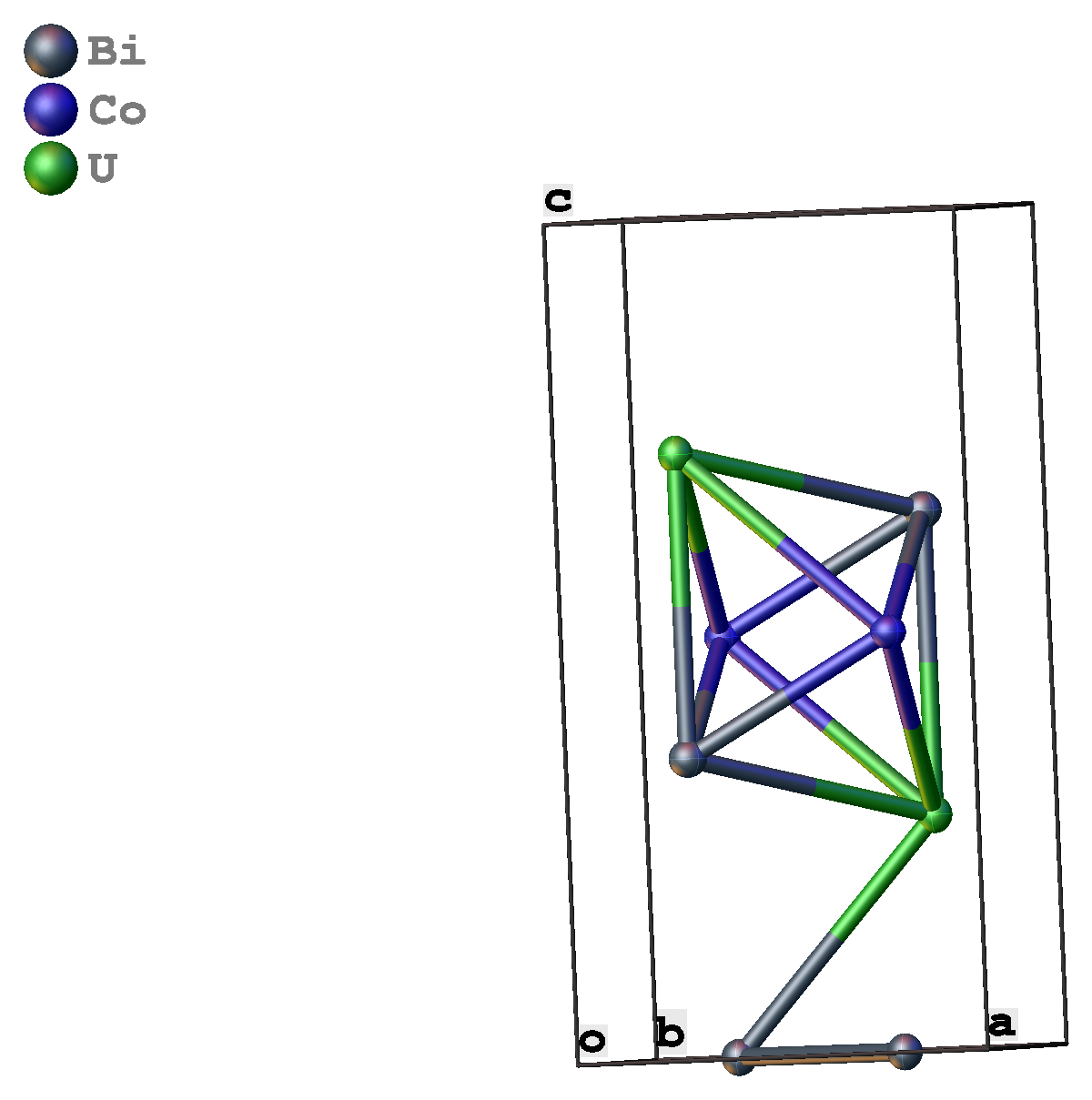 \| 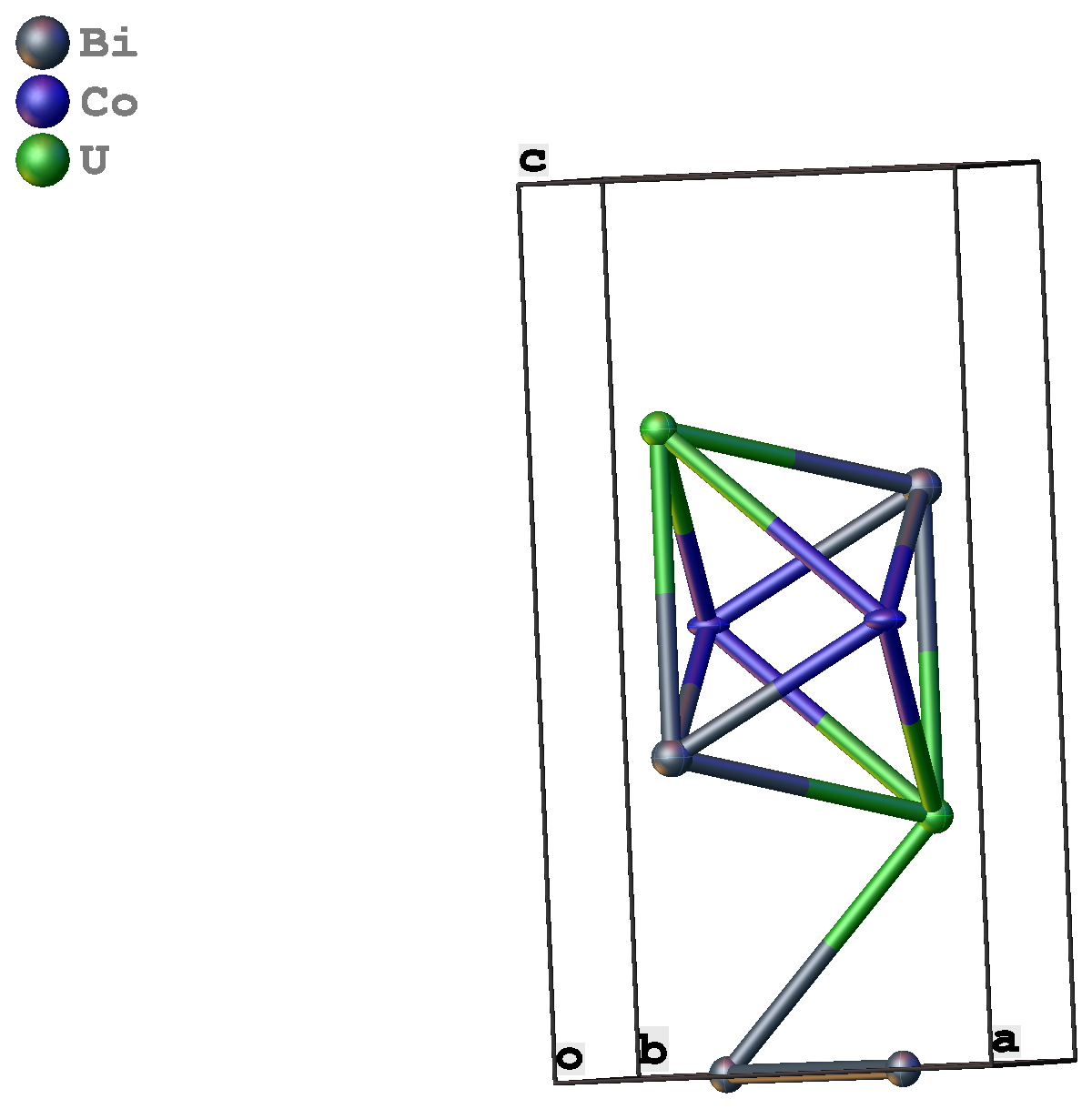 \| 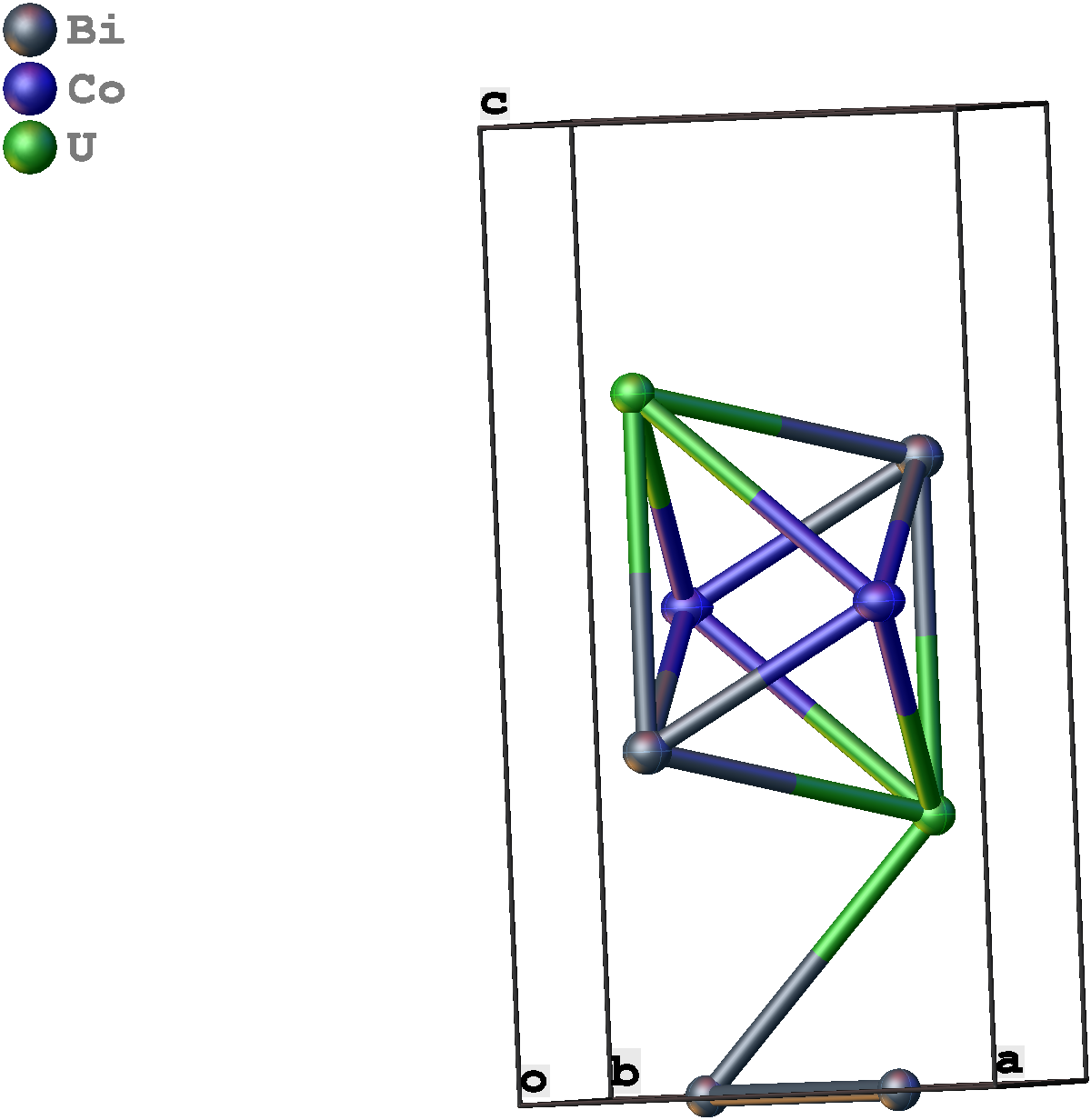 \| \| --- \| --- \| --- \| \| *x* = 0.145 \| *x* = 0.299 \| *x* = 0.335 \| \|  \| \| \| \| 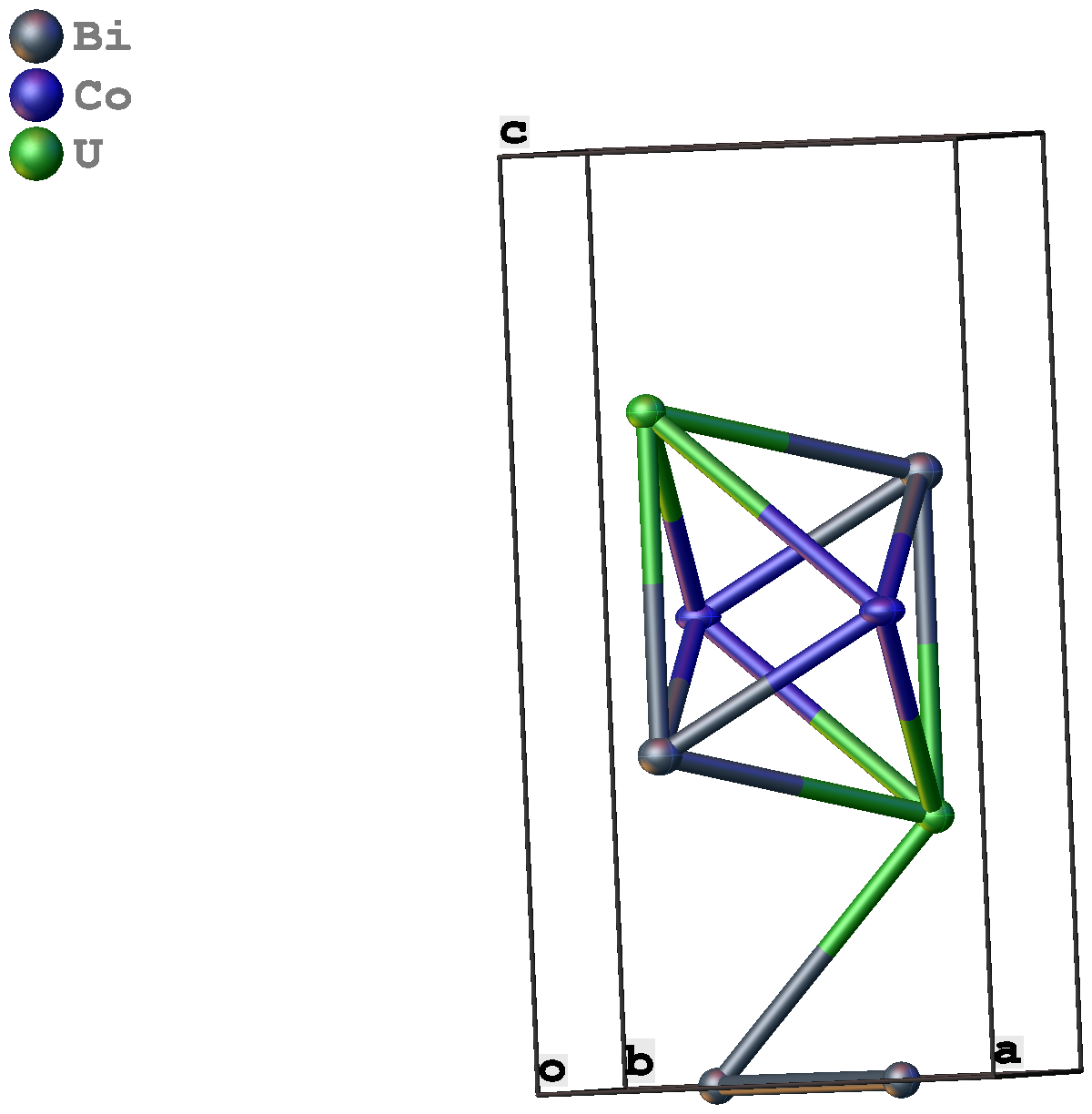 \| 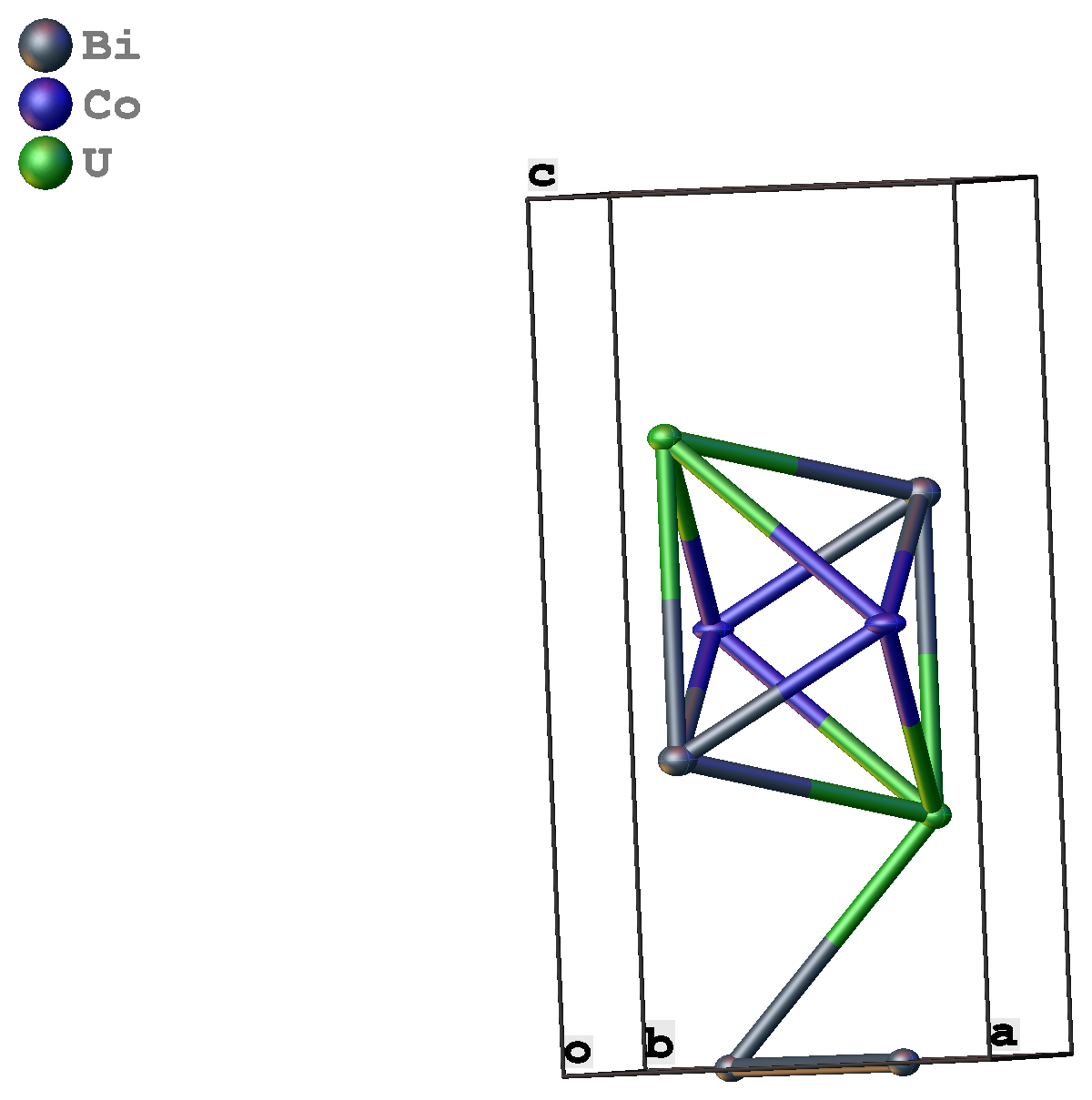 \| 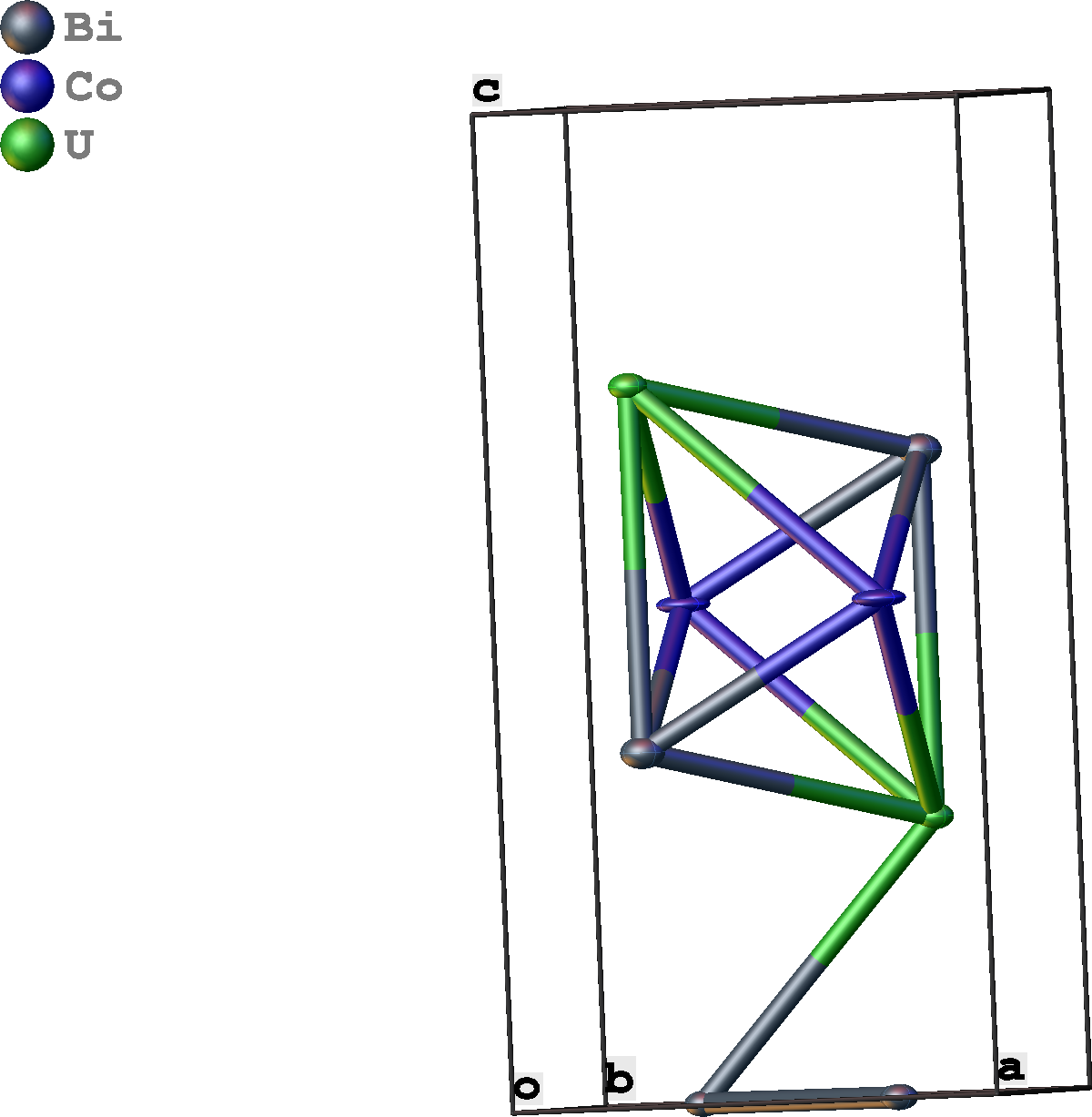 \| \| *x* = 0.343 \| *x* = 0.380 \| *x* = 0.391 \|   Figure S4. A view on the UCo*_x_*Bi_2_ (*x* = 0.145 – 0.391) unit cells. The atoms shown as thermal ellipsoids with 50% probability.   \| 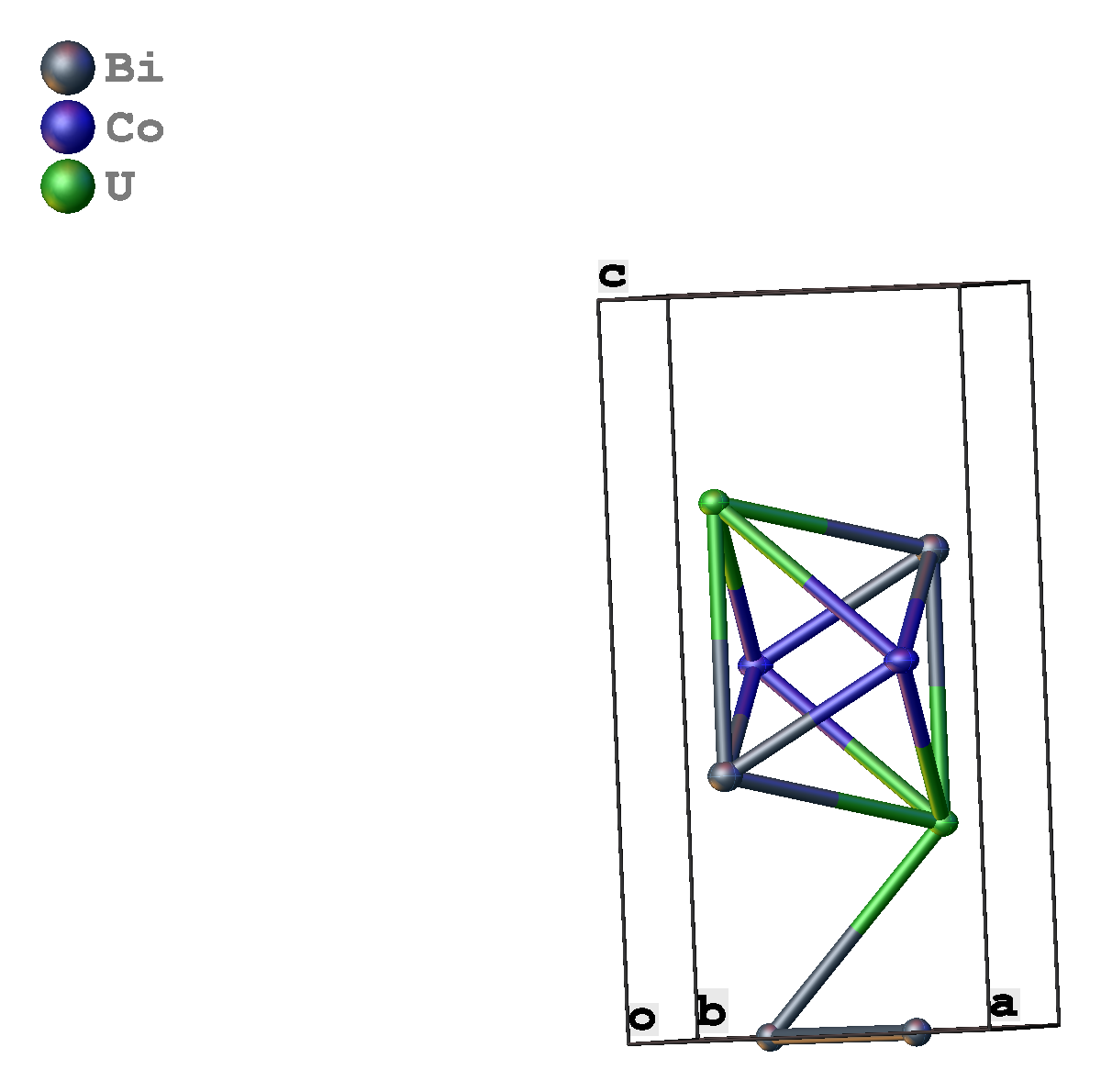 \| 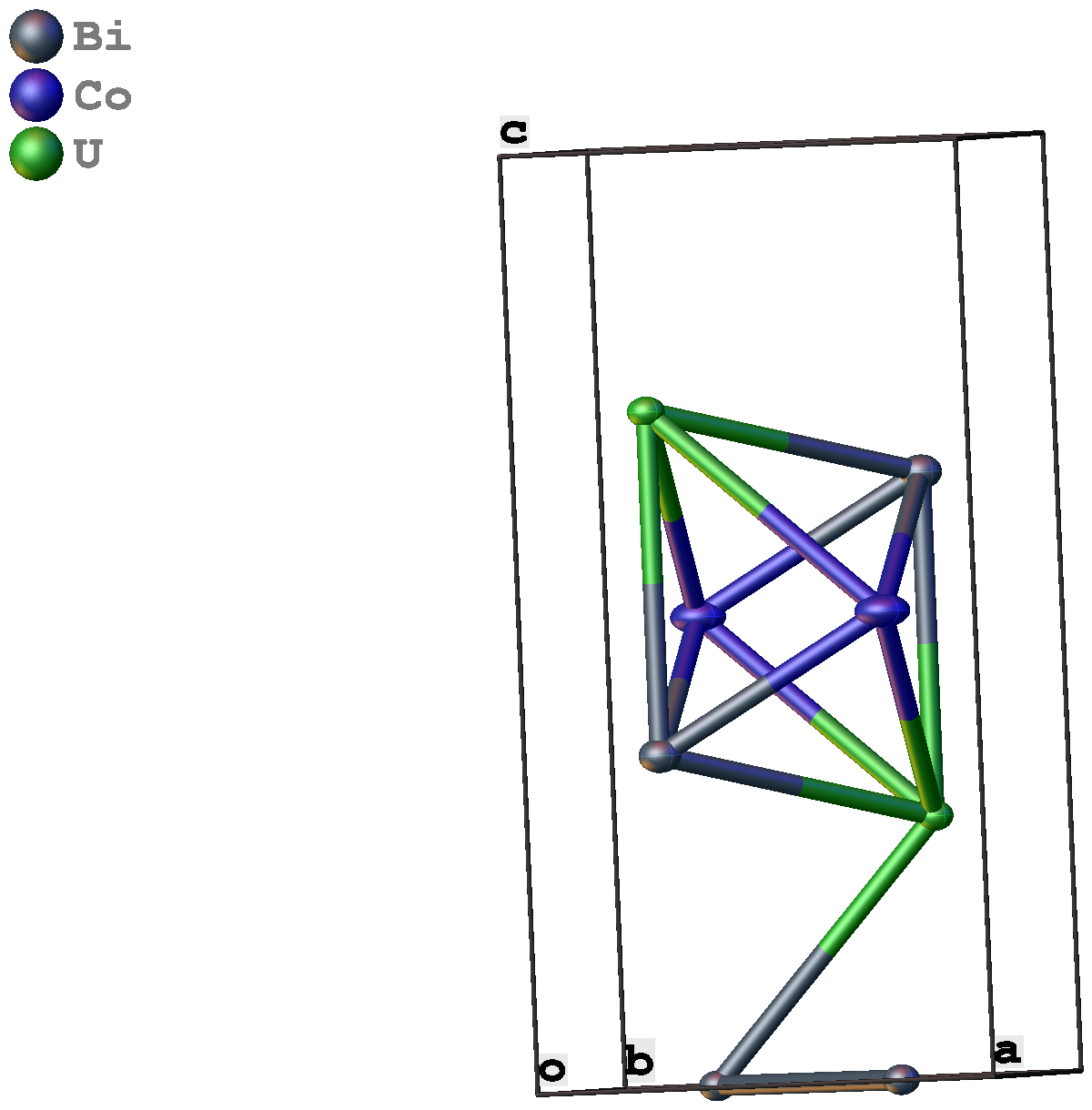 \| 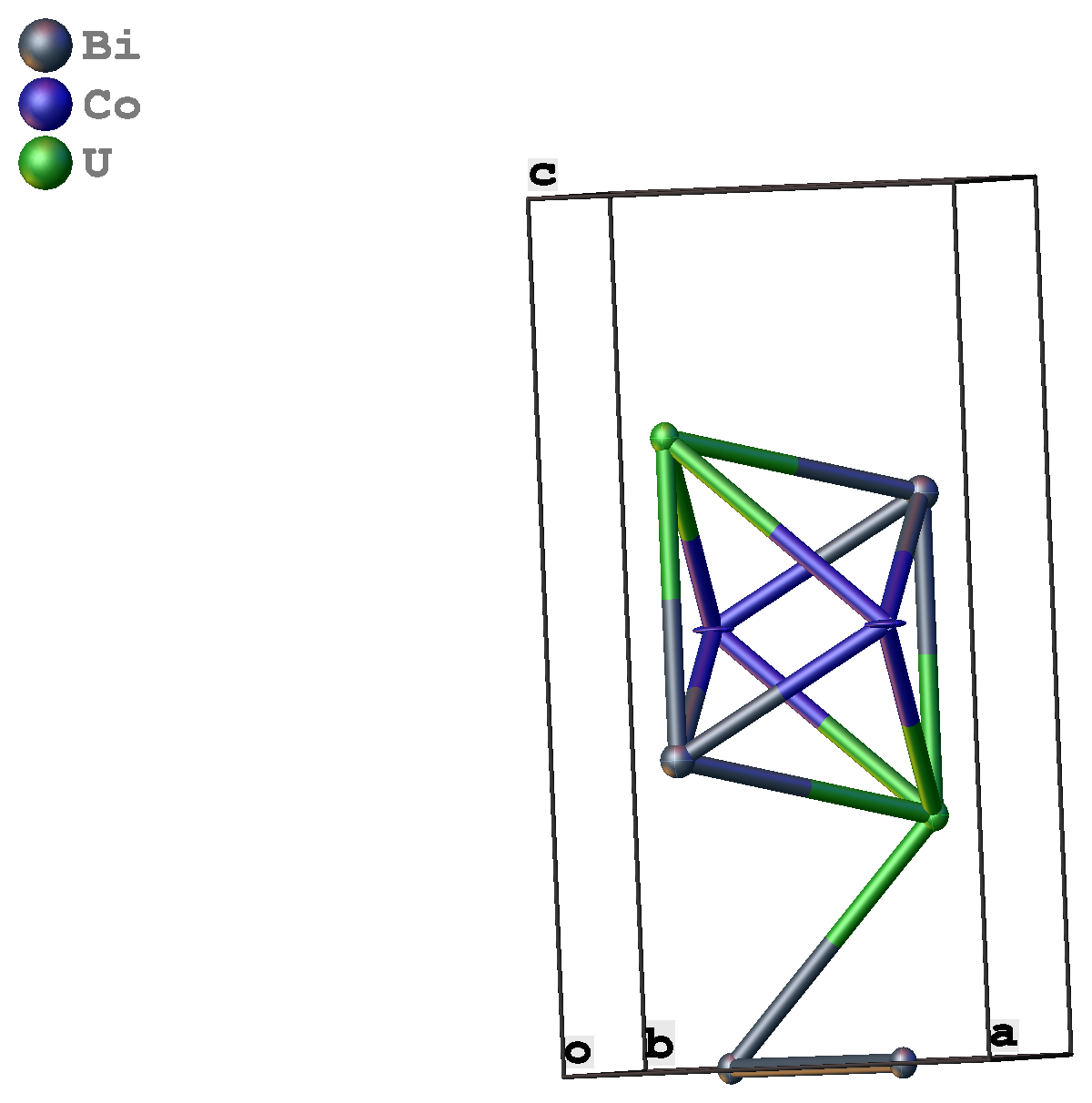 \| \| --- \| --- \| --- \| \| *x* = 0.399 \| *x* = 0.402 \| *x* = 0.404 \|  \| 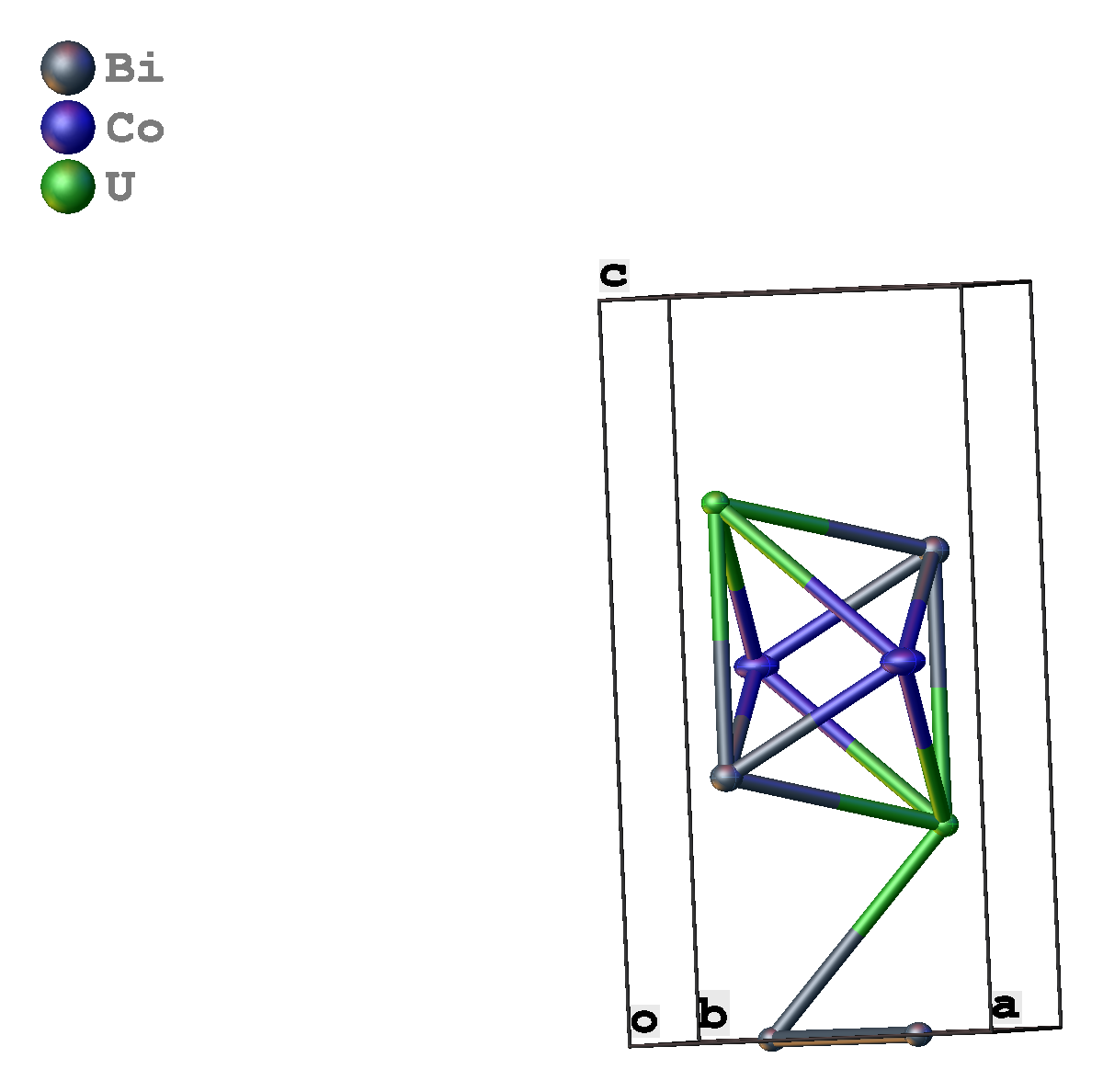 \| 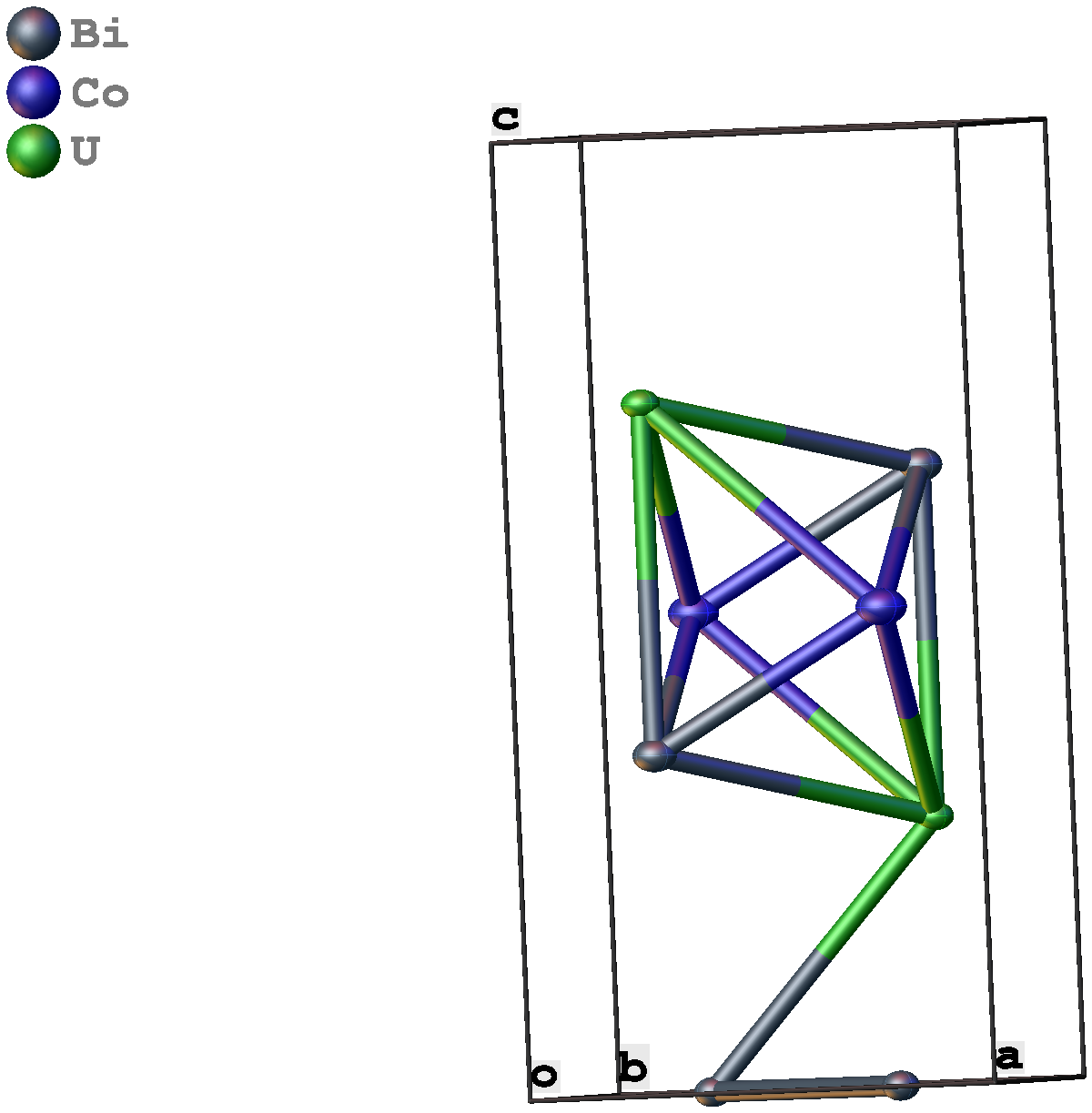 \| 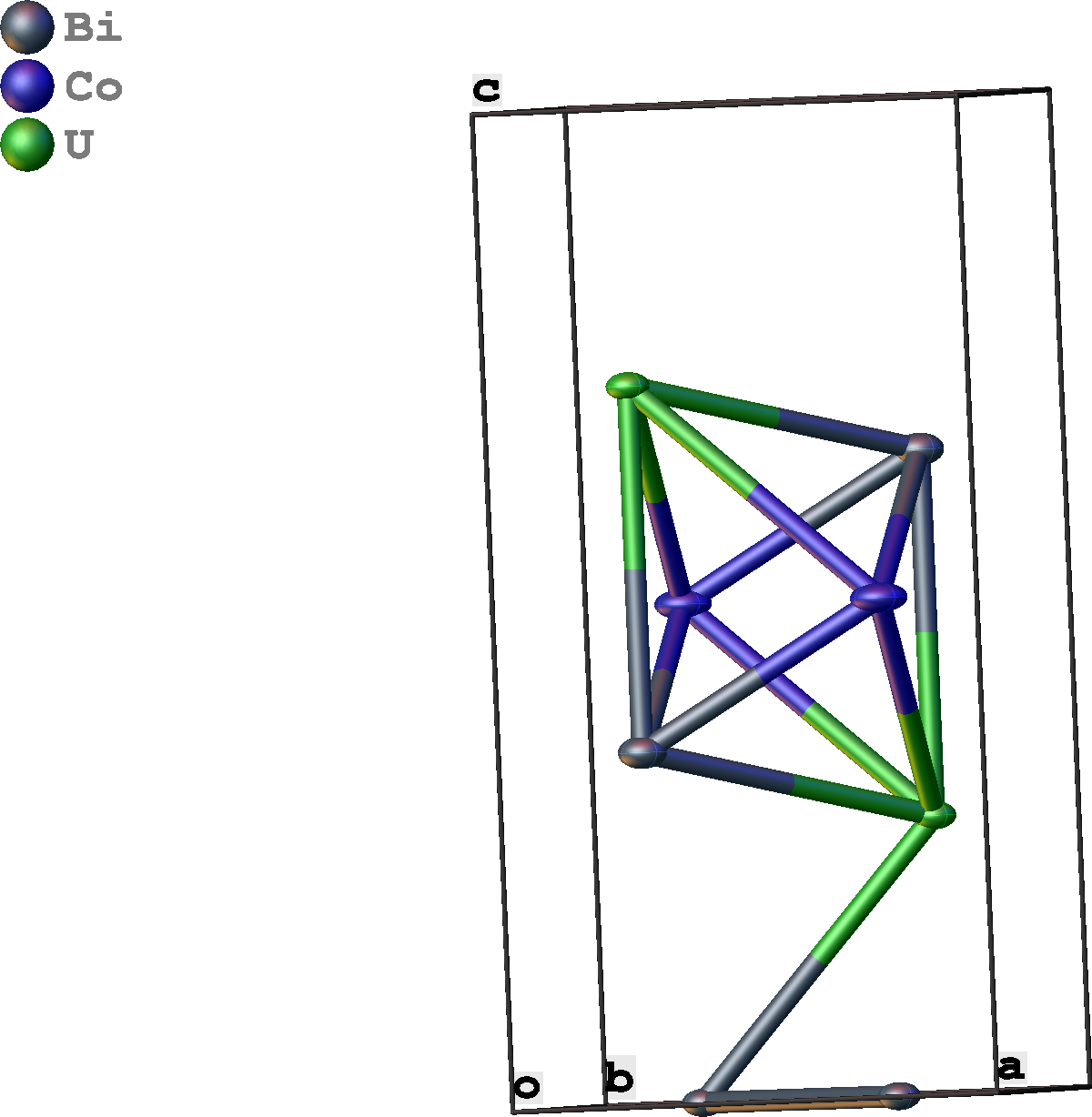 \| \| --- \| --- \| --- \| \| *x* = 0.416 \| *x* = 0.425 \| *x* = 0.428 \|   Figure S5. A view on the UCo*_x_*Bi_2_ (*x* = 0.399 – 0.428) unit cells. The atoms shown as thermal ellipsoids with 50% probability.   \| 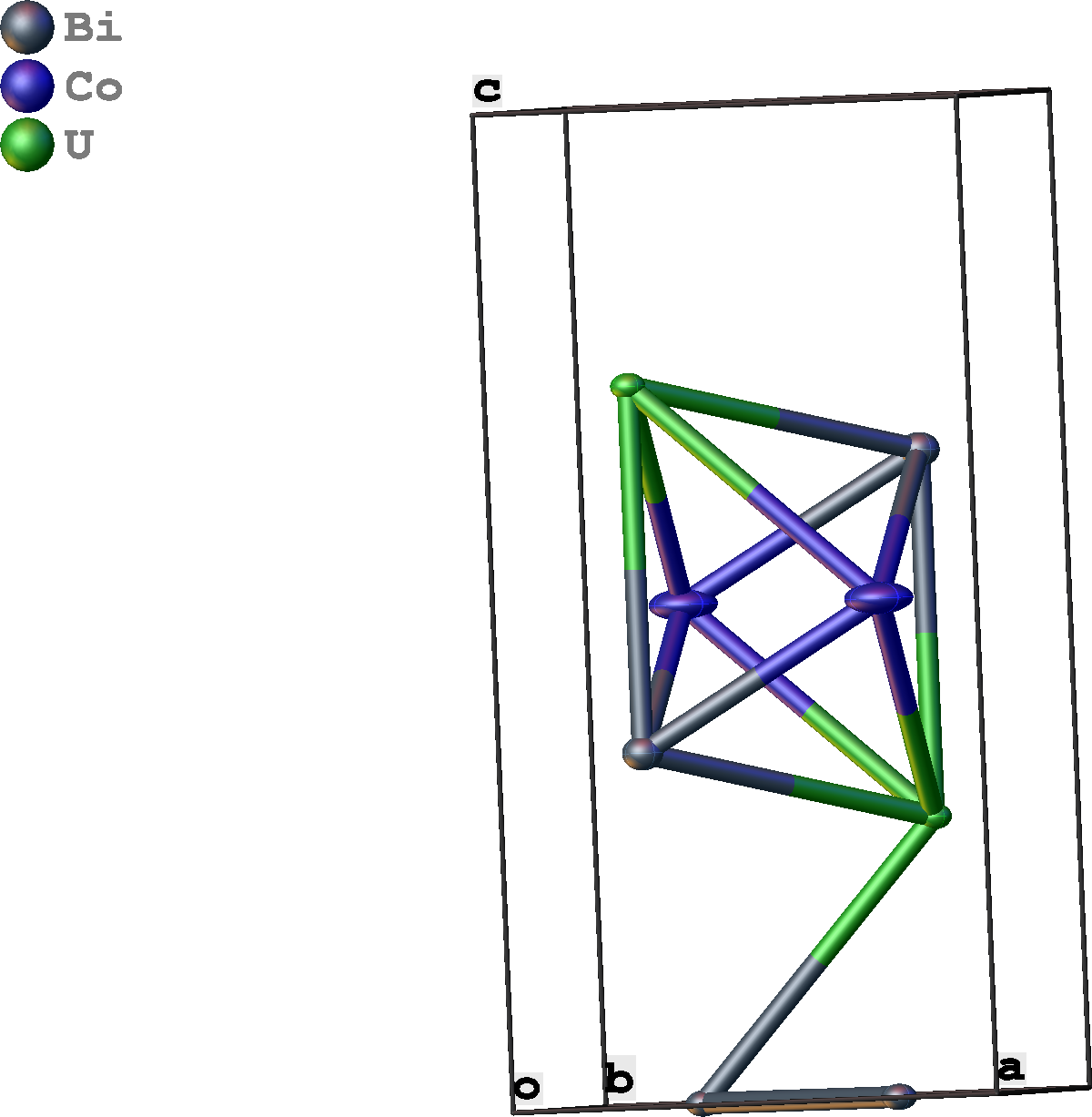 \| \| --- \| \| *x* = 0.472 \|   Figure S6. A view on the UCo*_x_*Bi_2_ (*x* = 0.472) unit cells. The atoms shown as thermal ellipsoids with 50% probability.   \| 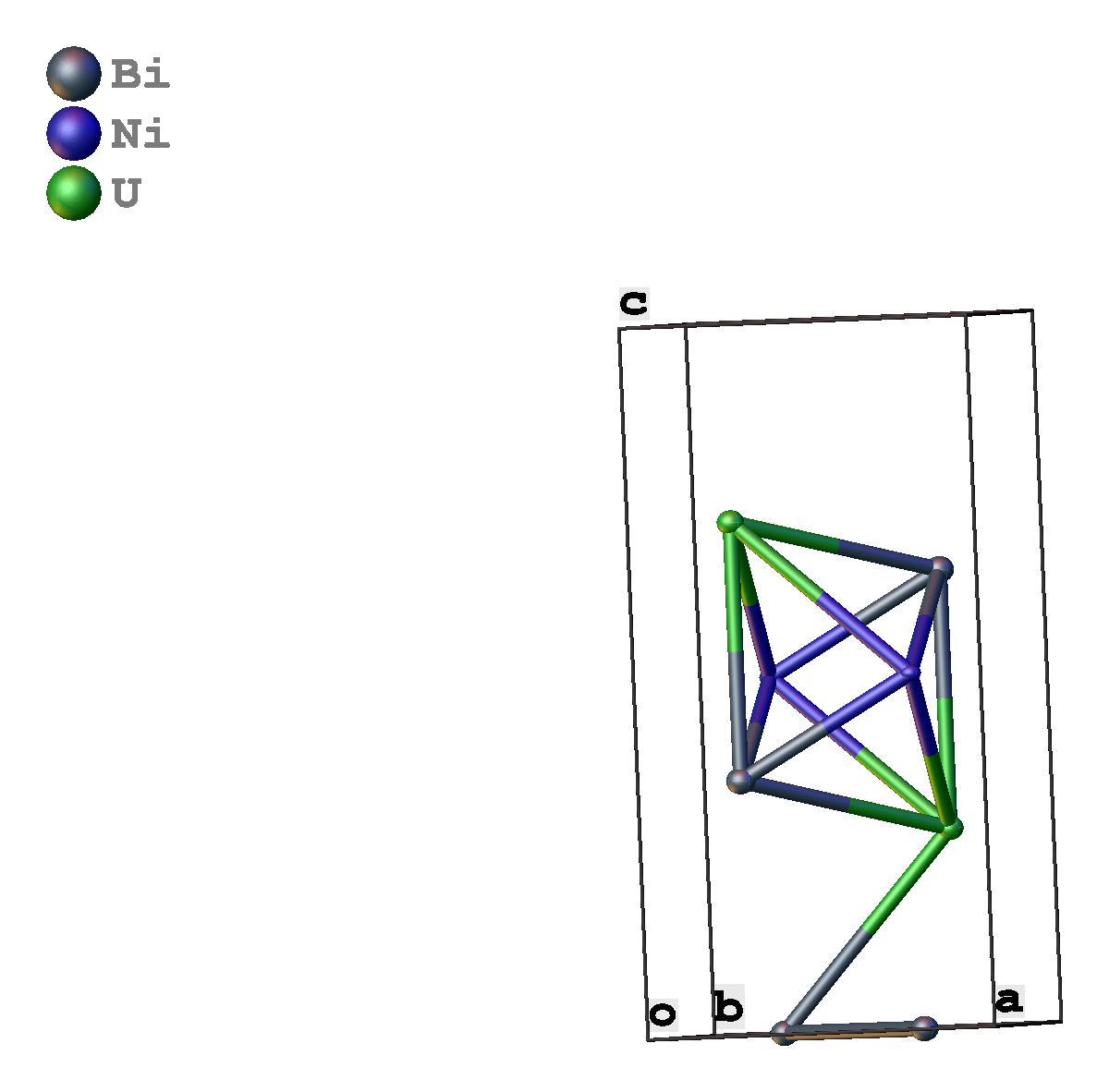 \| \| --- \| \| *x* = 0.13 \|   Figure S7. A view on the UNi*_x_*Bi_2_ (*x* = 0.13) unit cell. The atoms shown as thermal ellipsoids with 50% probability. |
| --- | --- | --- | --- | --- | --- | --- | --- | --- | --- | --- | --- | --- | --- | --- | --- | --- | --- | --- | --- | --- | --- | --- | --- | --- | --- | --- | --- | --- | --- | --- | --- |

Table S22. Summary of SEM EDS data of reaction HL27C. The reaction was a flux reaction with the ratio of 1:3:13 U:Co:Bi.

|  | | | **Atomic %'s:** | | | **Molar Ratios:** | | | **Average:** | | |
| --- | --- | --- | --- | --- | --- | --- | --- | --- | --- | --- | --- |
| **Sample ID:** | **Crystal #:** | **Site #:** | **U** | **Co** | **Bi** | **U** | **Co** | **Bi** | **U** | **Co** | **Bi** |
| HL 27C | 1 | 1 | 29.9 | 10.0 | 60.1 | 1.00 | 0.33 | 2 | 1.0 | 0.34 | 2 |
|  |  | 2 | 29.8 | 10.0 | 60.1 | 0.99 | 0.33 | 2 |  |  |  |
|  |  | 3 | 29.9 | 10.4 | 59.7 | 1.00 | 0.35 | 2 |  |  |  |
|  | 2 | 1 | 29.9 | 11.3 | 58.8 | 1.02 | 0.38 | 2 | 1.0 | 0.39 | 2 |
|  |  | 2 | 29.7 | 11.4 | 58.9 | 1.01 | 0.39 | 2 |  |  |  |
|  |  | 3 | 29.7 | 11.4 | 58.9 | 1.01 | 0.39 | 2 |  |  |  |
|  | 3 | 1 | 30.2 | 10.5 | 59.3 | 1.02 | 0.35 | 2 | 1.0 | 0.36 | 2 |
|  |  | 2 | 29.8 | 10.4 | 59.8 | 1.00 | 0.35 | 2 |  |  |  |
|  |  | 3 | 29.5 | 11.4 | 59.1 | 1.00 | 0.39 | 2 |  |  |  |


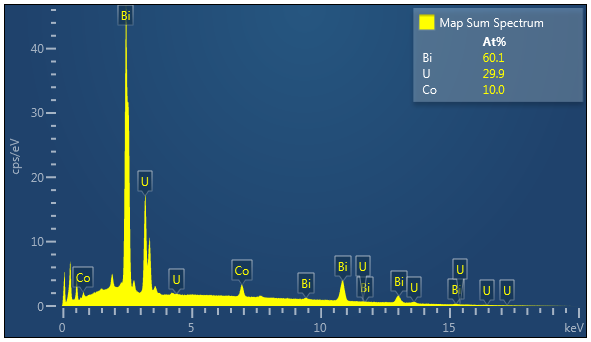


Figure S8. EDS spectrum of sample HL27C crystal 1 site 1


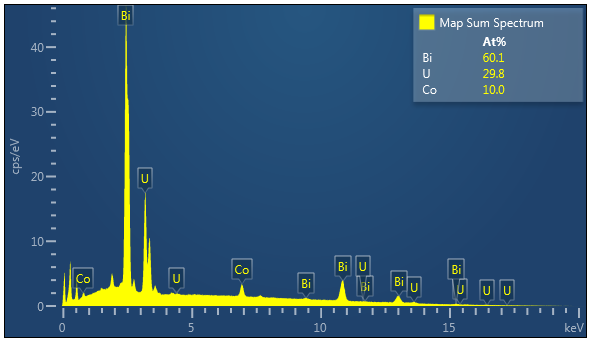


Figure S9. EDS spectrum of sample HL27C crystal 1 site 2


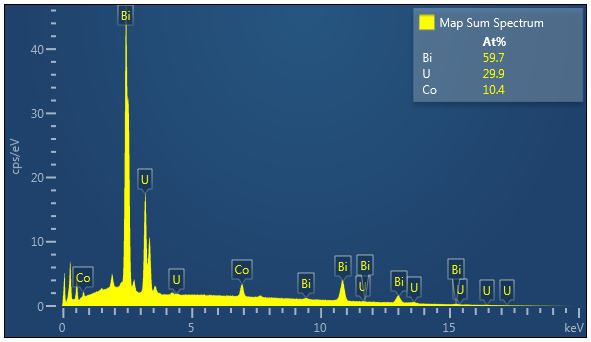


Figure S10. EDS spectrum of sample HL27C crystal 1 site 3


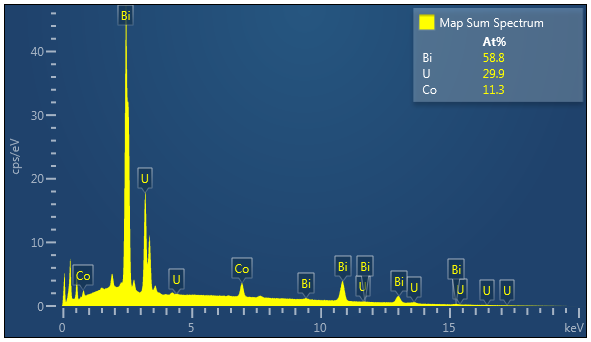


Figure S11. EDS spectrum of sample HL27C crystal 2 site 1


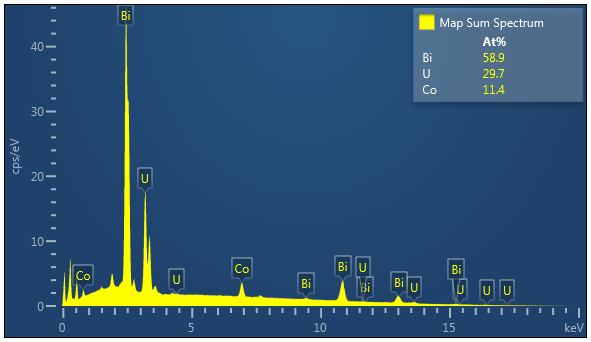


Figure S12. EDS spectrum of sample HL27C crystal 2 site 2


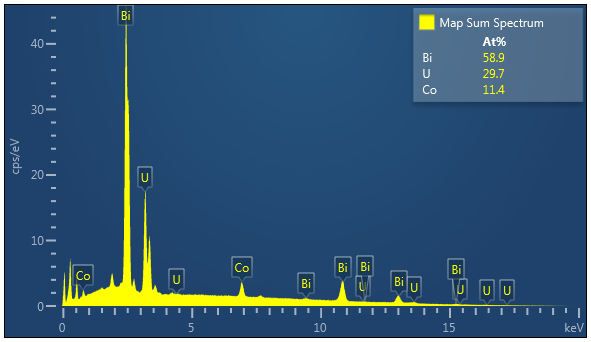


Figure S13. EDS spectrum of sample HL27C crystal 2 site 3


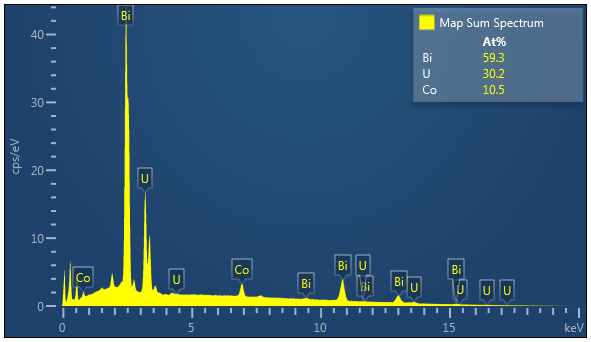


Figure S14. EDS spectrum of sample HL27C crystal3 site 1


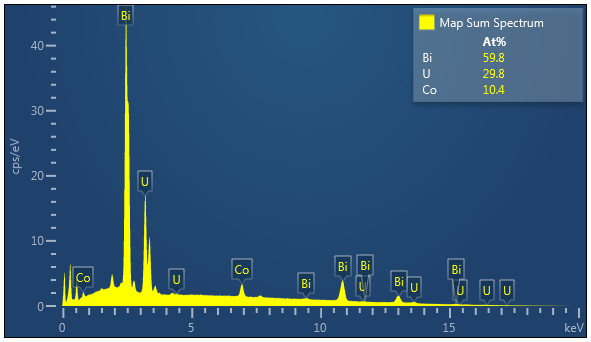


Figure S15. EDS spectrum of sample HL27C crystal3 site 2


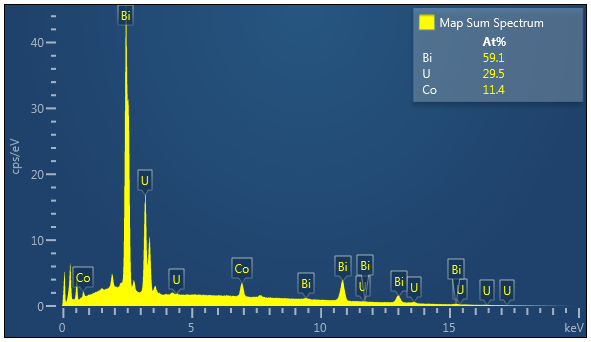


Figure S16. EDS spectrum of sample HL27C crystal3 site 3

Table S23. Summary of SEM EDS data of reaction HL27D. The reaction was a flux reaction with the ratio of 1:1:13 U:Co:Bi.

|  | | | **Atomic %'s:** | | | **Molar Ratios:** | | | **Average:** | | |
| --- | --- | --- | --- | --- | --- | --- | --- | --- | --- | --- | --- |
| **Sample ID:** | **Crystal #:** | **Site #:** | **U** | **Co** | **Bi** | **U** | **Co** | **Bi** | **U** | **Co** | **Bi** |
| HL 27D | 1 | 1 | 30.2 | 9.2 | 60.6 | 1.00 | 0.30 | 2 | 1.0 | 0.30 | 2 |
|  |  | 2 | 30.3 | 9.1 | 60.6 | 1.00 | 0.30 | 2 |  |  |  |
|  |  | 3 | 30.6 | 8.6 | 60.8 | 1.01 | 0.28 | 2 |  |  |  |
|  | 2 | 1 | 31.1 | 7.4 | 61.5 | 1.01 | 0.24 | 2 | 1.0 | 0.23 | 2 |
|  |  | 2 | 31.3 | 7.1 | 61.7 | 1.01 | 0.23 | 2 |  |  |  |
|  |  | 3 | 30.9 | 7.1 | 62.0 | 1.00 | 0.23 | 2 |  |  |  |
|  | 3 | 1 | 30.1 | 9.5 | 60.4 | 1.00 | 0.31 | 2 | 1.0 | 0.34 | 2 |
|  |  | 2 | 30.4 | 10.2 | 59.5 | 1.02 | 0.34 | 2 |  |  |  |
|  |  | 3 | 30.0 | 10.8 | 59.2 | 1.01 | 0.36 | 2 |  |  |  |


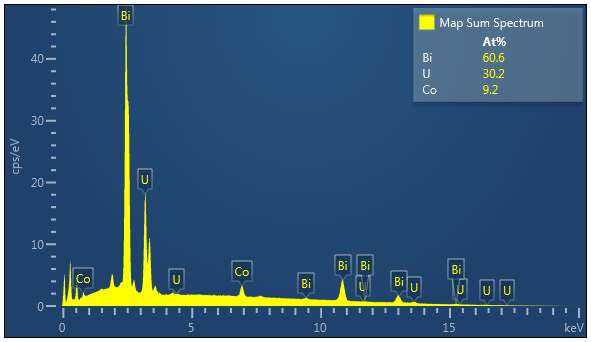


Figure S17. EDS spectrum of sample HL27D crystal 1 site 1


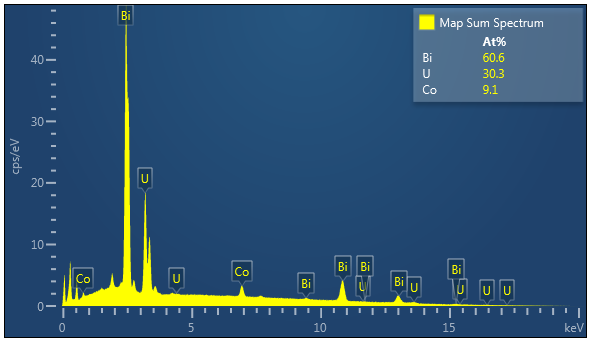


Figure S18. EDS spectrum of sample HL27D crystal 1 site 2


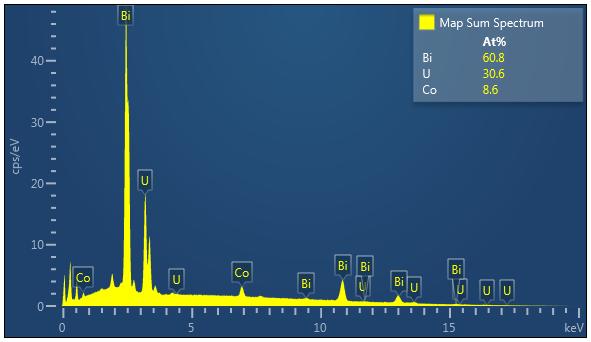


Figure S19. EDS spectrum of sample HL27D crystal 1 site 3


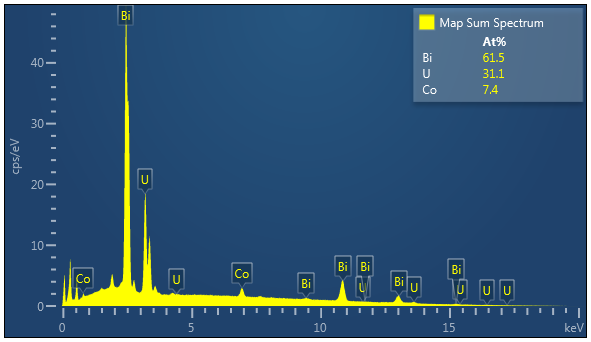


Figure S20. EDS spectrum of sample HL27D crystal 2 site 1


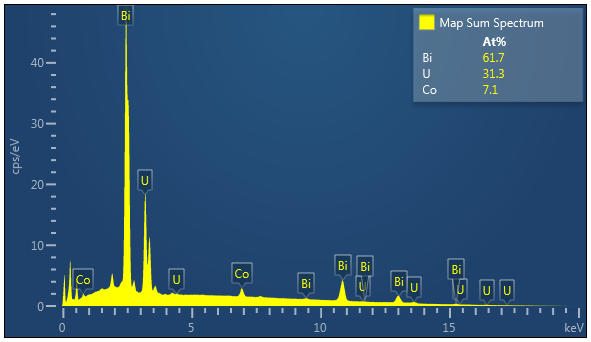


Figure S21. EDS spectrum of sample HL27D crystal 2 site 2


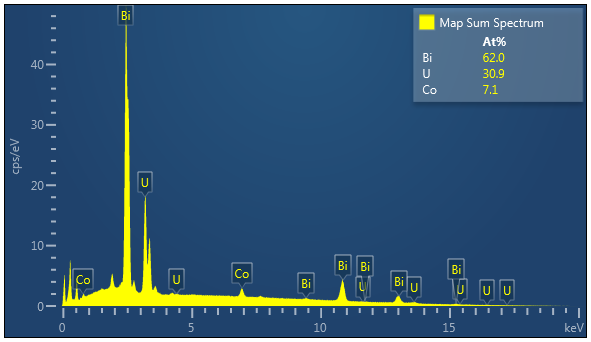


Figure S22. EDS spectrum of sample HL27D crystal 2 site 3


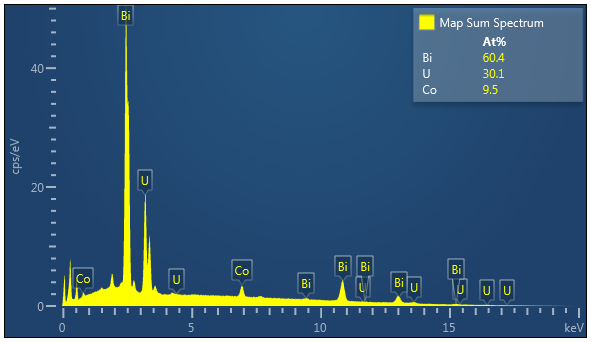


Figure S23. EDS spectrum of sample HL27D crystal 3 site 1


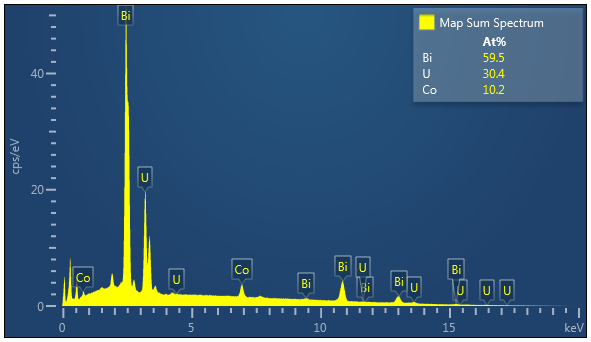


Figure S24. EDS spectrum of sample HL27D crystal 3 site 2


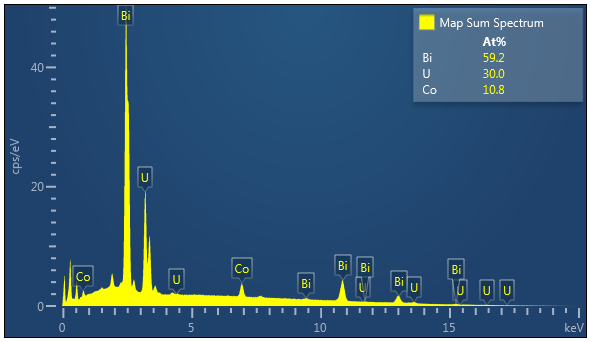


Figure S25. EDS spectrum of sample HL27D crystal 3 site 3

Table S24. Summary of SEM EDS data of reaction HL32F. The reaction was a flux reaction with the ratio of 1:1:13 U:Co:Bi.

|  | | | **Atomic %'s:** | | | **Molar Ratios:** | | | **Average:** | | |
| --- | --- | --- | --- | --- | --- | --- | --- | --- | --- | --- | --- |
| **Sample ID:** | **Crystal #:** | **Site #:** | **U** | **Co** | **Bi** | **U** | **Co** | **Bi** | **U** | **Co** | **Bi** |
| HL 32F | 1 | 1 | 27.3 | 8.9 | 63.8 | 0.86 | 0.28 | 2 | 0.87 | 0.26 | 2 |
|  |  | 2 | 27.4 | 7.9 | 64.7 | 0.85 | 0.24 | 2 |  |  |  |
|  |  | 3 | 28.6 | 8.1 | 63.3 | 0.90 | 0.26 | 2 |  |  |  |
|  | 2 | 1 | No useable data (too decomposed) | | | | | |  |  |  |
|  |  | 2 |  |  |  |  |  |  |  |  |  |
|  |  | 3 |  |  |  |  |  |  |  |  |  |
|  | 3 | 1 | 28.3 | 9.5 | 62.2 | 0.91 | 0.31 | 2 | 0.82 | 0.27 | 2 |
|  |  | 2 | 25.4 | 8.5 | 66 | 0.77 | 0.26 | 2 |  |  |  |
|  |  | 3 | 25.7 | 8.6 | 65.7 | 0.78 | 0.26 | 2 |  |  |  |


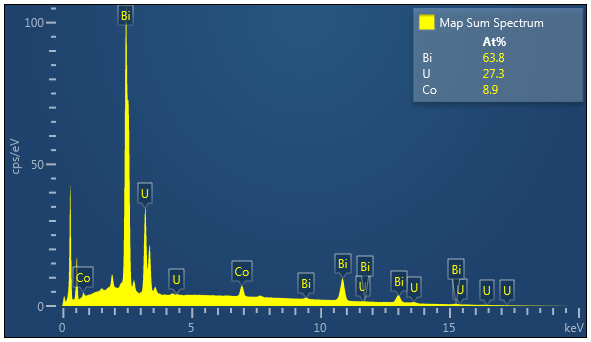


Figure S26. EDS spectrum of sample HL32F crystal 1 site 1


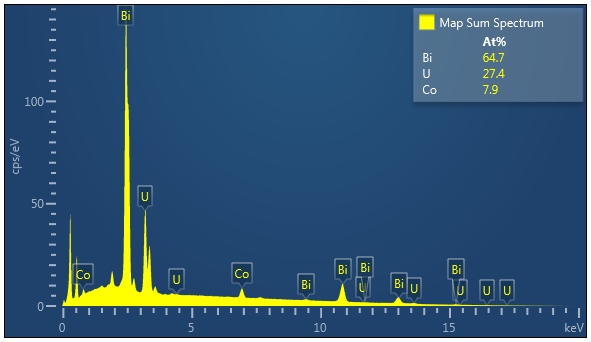


Figure S27. EDS spectrum of sample HL32F crystal 1 site 2


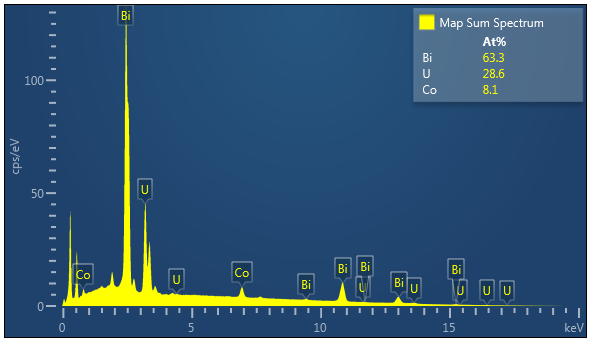


Figure S28. EDS spectrum of sample HL32F crystal 1 site 3


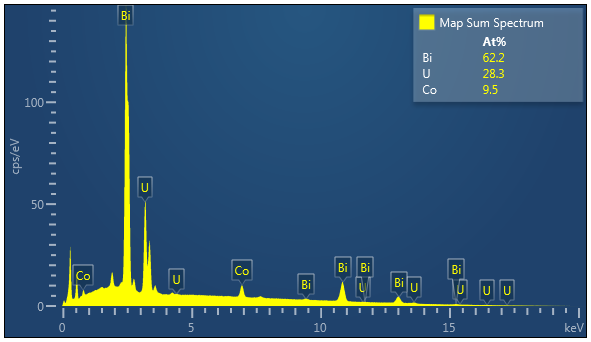


Figure S29. EDS spectrum of sample HL32F crystal 3 site 1


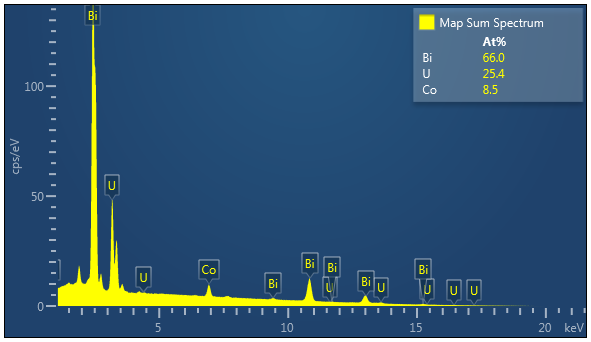


Figure S30. EDS spectrum of sample HL32F crystal 3 site 2


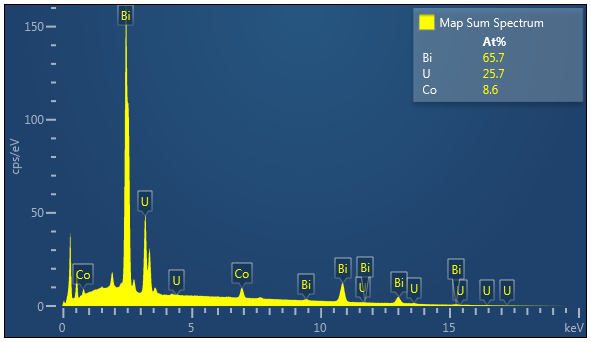


Figure S31. EDS spectrum of sample HL32F crystal 3 site 3

Table S25. Summary of SEM EDS data of reaction HL27F. The reaction was a flux reaction with the ratio of 2:3:27 U:Co:Bi.

|  | | | **Atomic %'s:** | | | **Molar Ratios:** | | | **Average:** | | |
| --- | --- | --- | --- | --- | --- | --- | --- | --- | --- | --- | --- |
| **Sample ID:** | **Crystal #:** | **Site #:** | **U** | **Co** | **Bi** | **U** | **Co** | **Bi** | **U** | **Co** | **Bi** |
| HL 27F | 1 | 1 | 29.1 | 12.9 | 57.9 | 1.01 | 0.45 | 2 | 1.01 | 0.44 | 2 |
|  |  | 2 | 29.2 | 12.9 | 57.9 | 1.01 | 0.45 | 2 |  |  |  |
|  |  | 3 | 29.3 | 12.7 | 58.0 | 1.01 | 0.44 | 2 |  |  |  |
|  | 2 | 1 | 29.0 | 12.5 | 58.5 | 0.99 | 0.43 | 2 | 1.00 | 0.43 | 2 |
|  |  | 2 | 29.5 | 12.7 | 57.8 | 1.02 | 0.44 | 2 |  |  |  |
|  |  | 3 | 29.2 | 12.2 | 58.6 | 1.00 | 0.42 | 2 |  |  |  |
|  | 3 | 1 | 29.2 | 12.2 | 58.6 | 1.00 | 0.42 | 2 | 1.00 | 0.41 | 2 |
|  |  | 2 | 29.4 | 12.1 | 58.5 | 1.01 | 0.41 | 2 |  |  |  |
|  |  | 3 | 29.3 | 12.1 | 58.5 | 1.00 | 0.41 | 2 |  |  |  |


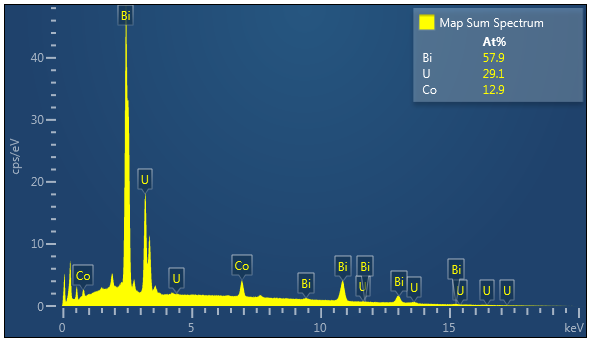


Figure S32. EDS spectrum of sample HL27F crystal 1 site 1


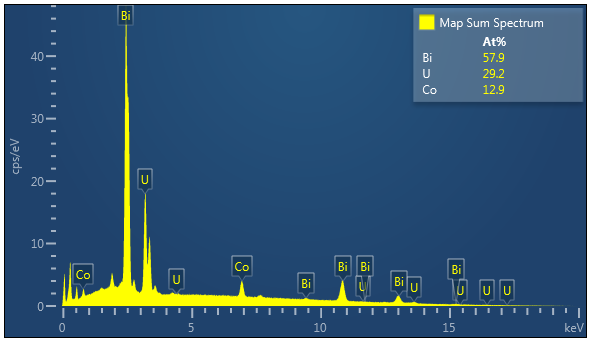


Figure S33. EDS spectrum of sample HL27F crystal 1 site 2


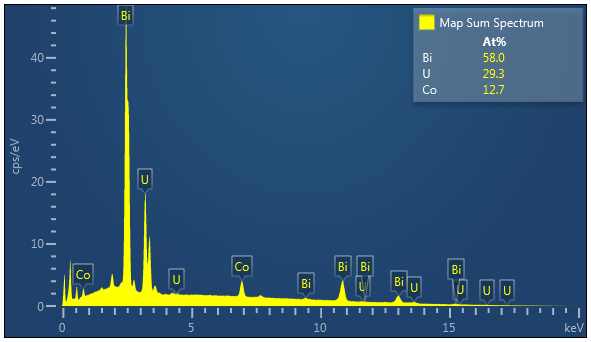


Figure S34. EDS spectrum of sample HL27F crystal 1 site 3


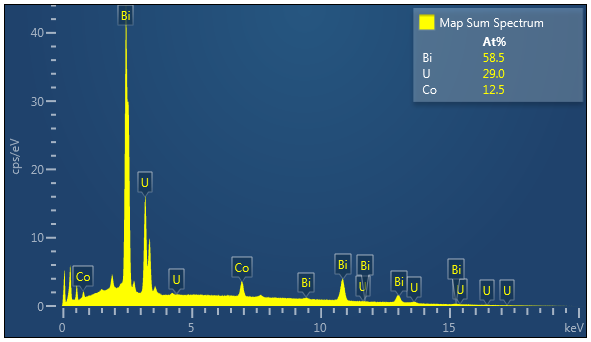


Figure S35. EDS spectrum of sample HL27F crystal 2 site 1


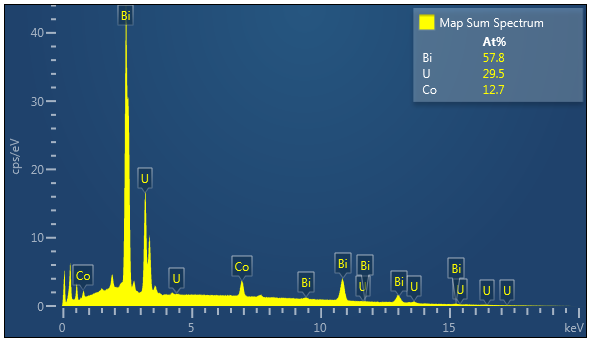


Figure S36. EDS spectrum of sample HL27F crystal 2 site 2


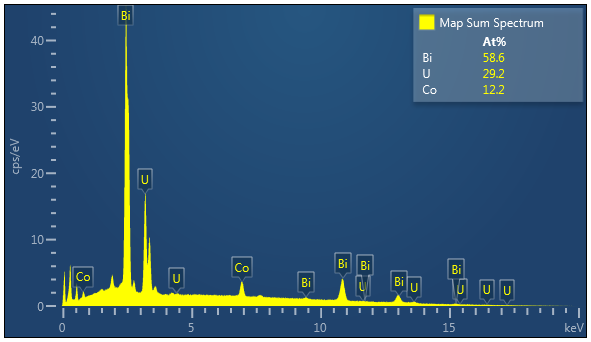


Figure S37. EDS spectrum of sample HL27F crystal 2 site 3


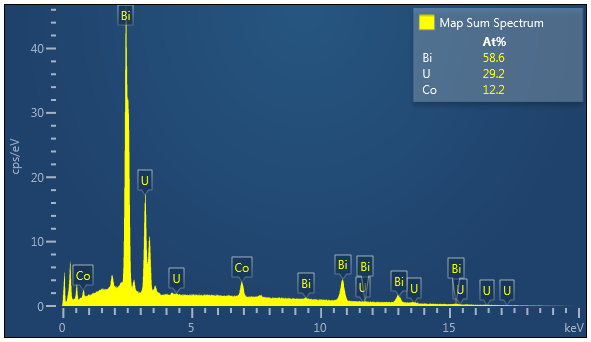


Figure S38. EDS spectrum of sample HL27F crystal 3 site 1


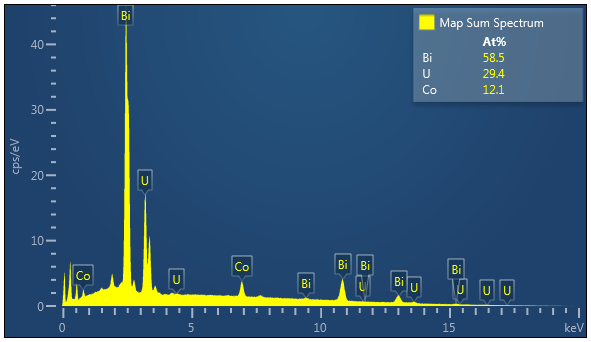


Figure S39. EDS spectrum of sample HL27F crystal 3 site 2


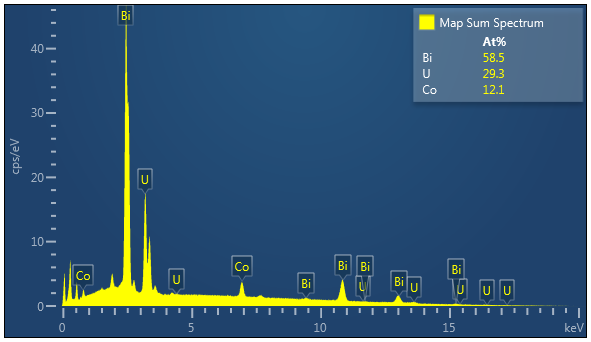


Figure S40. EDS spectrum of sample HL27F crystal 3 site 3

Table S26. Summary of SEM EDS data of reaction HL32G. The reaction was a flux reaction with the ratio of 2:3:27 U:Co:Bi.

|  | | | **Atomic %'s:** | | | **Molar Ratios:** | | | **Average:** | | |
| --- | --- | --- | --- | --- | --- | --- | --- | --- | --- | --- | --- |
| **Sample ID:** | **Crystal #:** | **Site #:** | **U** | **Co** | **Bi** | **U** | **Co** | **Bi** | **U** | **Co** | **Bi** |
| HL 32G | 1 | 1 | 26.7 | 8.5 | 64.8 | 0.82 | 0.26 | 2 | 0.864 | 0.277 | 2 |
|  |  | 2 | 28.3 | 9.1 | 62.6 | 0.90 | 0.29 | 2 |  |  |  |
|  | 2 | 1 | No useable crystal left | | |  |  |  |  |  |  |
|  |  | 2 |  |  |  |  |  |  |  |  |  |
|  | 3 | 1 | 28.9 | 11.7 | 59.4 | 0.97 | 0.39 | 2 | 0.973 | 0.390 | 2 |
|  |  | 2 | 29.0 | 11.5 | 59.6 | 0.97 | 0.39 | 2 |  |  |  |


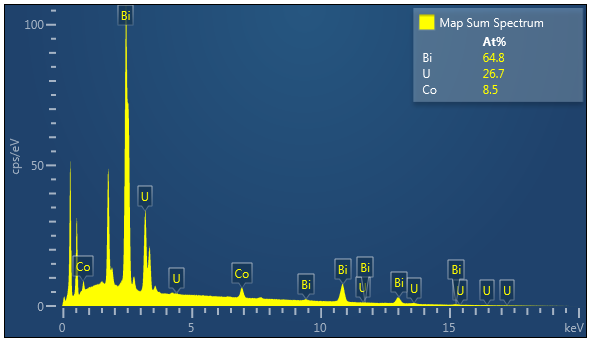


Figure S41. EDS spectrum of sample HL32G crystal 1 site 1


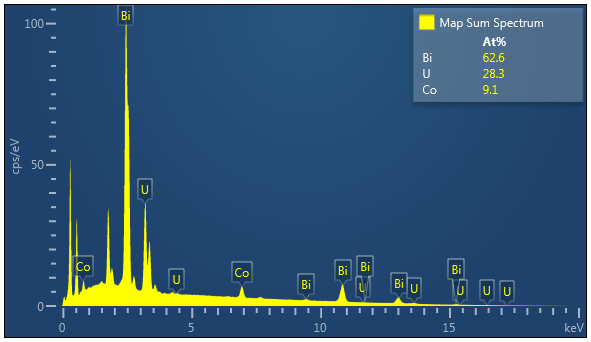


Figure S42. EDS spectrum of sample HL32G crystal 1 site 2


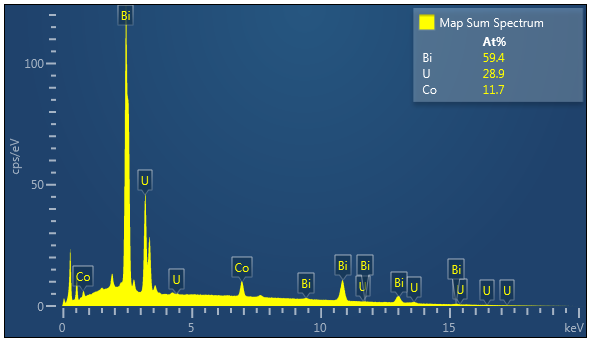


Figure S43. EDS spectrum of sample HL32G crystal 3 site 1


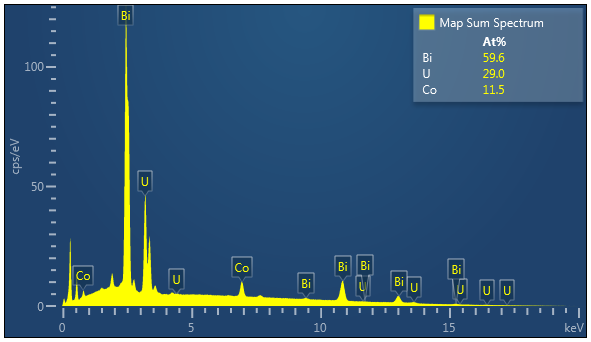


Figure S44. EDS spectrum of sample HL32G crystal 3 site 2

Table S27. Summary of SEM EDS data of reaction HL32I. The reaction was a flux reaction with the ratio of 1:2:13 U:Co:Bi.

|  | | | **Atomic %'s:** | | | **Molar Ratios:** | | | **Average:** | | |
| --- | --- | --- | --- | --- | --- | --- | --- | --- | --- | --- | --- |
| **Sample ID:** | **Crystal #:** | **Site #:** | **U** | **Co** | **Bi** | **U** | **Co** | **Bi** | **U** | **Co** | **Bi** |
| HL 32I | 1 | 1 | 29.6 | 11.0 | 59.5 | 0.99 | 0.37 | 2 | 1.0 | 0.38 | 2 |
|  |  | 2 | 28.9 | 12.0 | 59.2 | 0.98 | 0.41 | 2 |  |  |  |
|  |  | 3 | 29.3 | 11.2 | 59.4 | 0.99 | 0.38 | 2 |  |  |  |
|  | 2 | 1 | 28.7 | 12.0 | 59.3 | 0.97 | 0.40 | 2 | 1.0 | 0.41 | 2 |
|  |  | 2 | 28.6 | 11.8 | 59.6 | 0.96 | 0.40 | 2 |  |  |  |
|  |  | 3 | 28.8 | 12.5 | 58.7 | 0.98 | 0.43 | 2 |  |  |  |
|  | 3 | 1 | 28.5 | 12.0 | 59.5 | 0.96 | 0.40 | 2 | 1.0 | 0.41 | 2 |
|  |  | 2 | 28.6 | 12.0 | 59.3 | 0.96 | 0.40 | 2 |  |  |  |
|  |  | 3 | 28.6 | 12.1 | 59.3 | 0.96 | 0.41 | 2 |  |  |  |


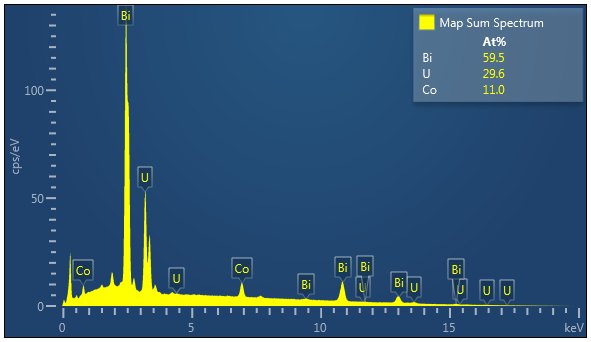


Figure S45. EDS spectrum of sample HL32I crystal 1 site 1


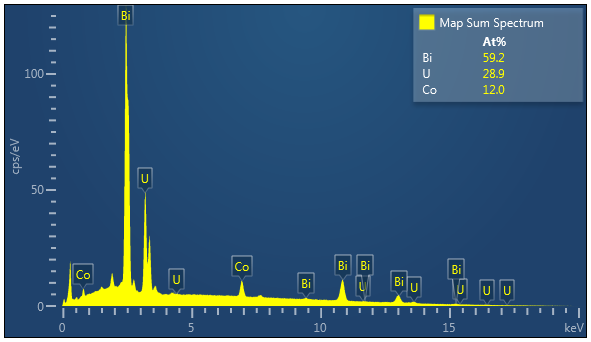


Figure S46. EDS spectrum of sample HL32I crystal 1 site 2


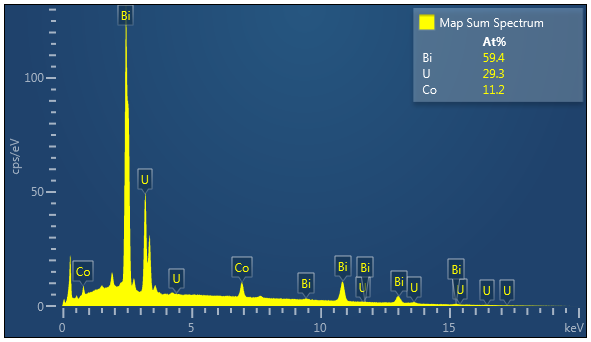


Figure S47. EDS spectrum of sample HL32I crystal 1 site 3


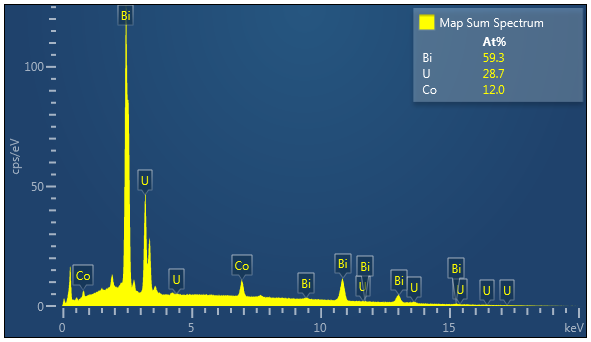


Figure S48. EDS spectrum of sample HL32I crystal 2 site 1


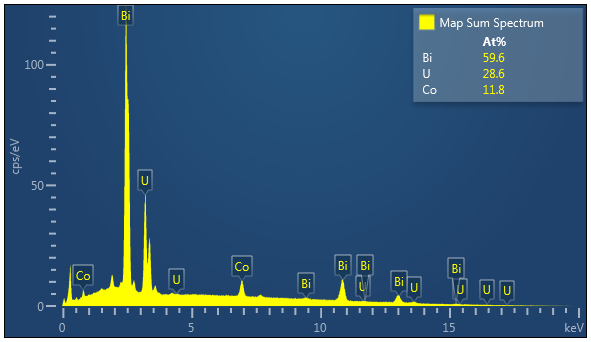


Figure S49. EDS spectrum of sample HL32I crystal 2 site 2


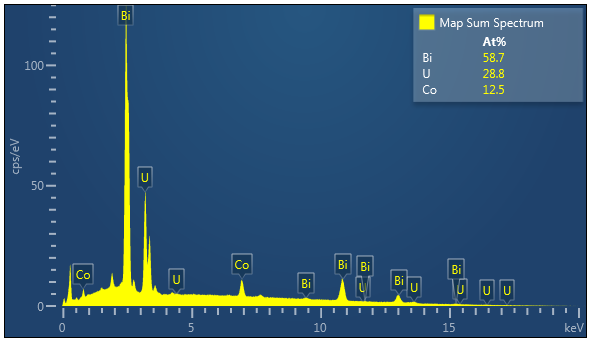


Figure S50. EDS spectrum of sample HL32I crystal 2 site 3


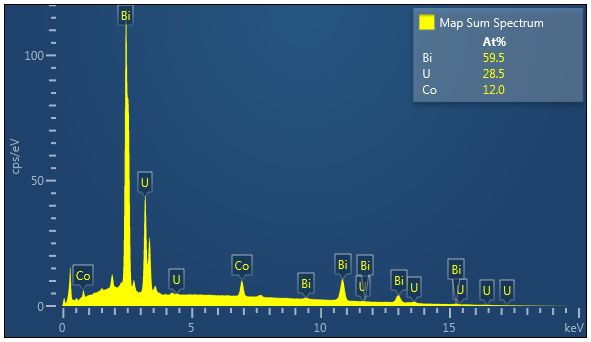


Figure S51. EDS spectrum of sample HL32I crystal 3 site 1


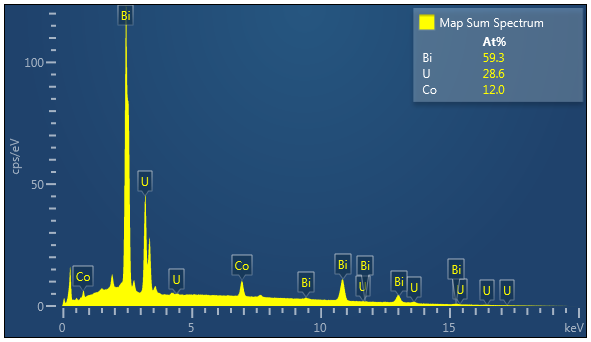


Figure S52. EDS spectrum of sample HL32I crystal 3 site 2


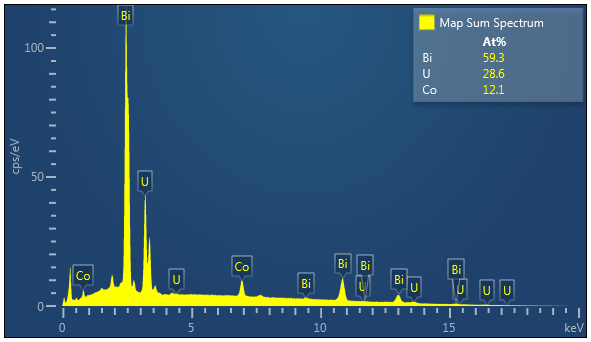


Figure S53. EDS spectrum of sample HL32I crystal 3 site 3

Table S28. Summary of SEM EDS data of reaction HL119O. The reaction was a flux reaction with the ratio of 1:3.5:20 U:Co:Bi.

|  | | | **Atomic %'s:** | | | **Molar Ratios:** | | | **Average:** | | |
| --- | --- | --- | --- | --- | --- | --- | --- | --- | --- | --- | --- |
| **Sample ID:** | **Crystal #:** | **Site #:** | **U** | **Co** | **Bi** | **U** | **Co** | **Bi** | **U** | **Co** | **Bi** |
| HL 119O | 2 | 1 | 29.4 | 12.4 | 58.2 | 1.01 | 0.43 | 2 | 1.01 | 0.42 | 2 |
|  |  | 2 | 29.7 | 11.9 | 58.4 | 1.02 | 0.41 | 2 |  |  |  |
|  |  | 3 | 29.5 | 12.1 | 58.4 | 1.01 | 0.41 | 2 |  |  |  |
|  | 3 | 1 | 29.8 | 12.8 | 57.5 | 1.04 | 0.45 | 2 | 1.03 | 0.44 | 2 |
|  |  | 2 | 29.8 | 12.4 | 57.8 | 1.03 | 0.43 | 2 |  |  |  |
|  |  | 3 | 29.6 | 12.7 | 57.8 | 1.02 | 0.44 | 2 |  |  |  |


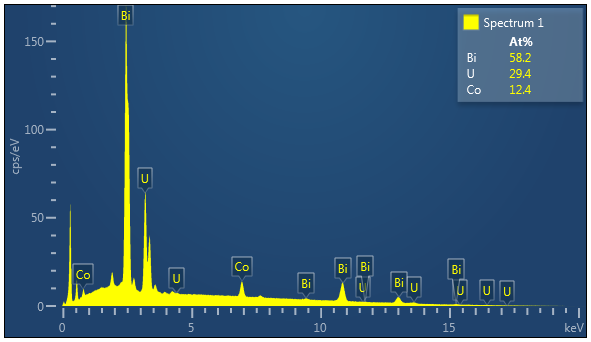


Figure S54. EDS spectrum of sample HL119O crystal 2 site 1


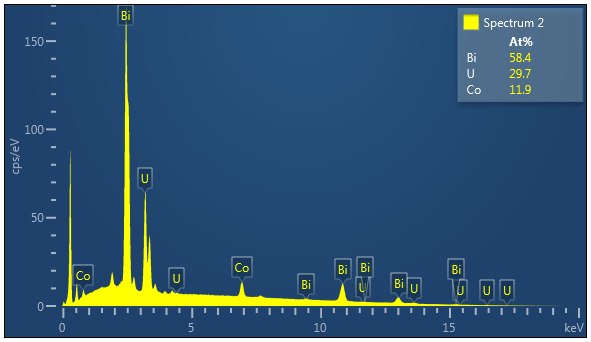


Figure S55. EDS spectrum of sample HL119O crystal 2 site 2


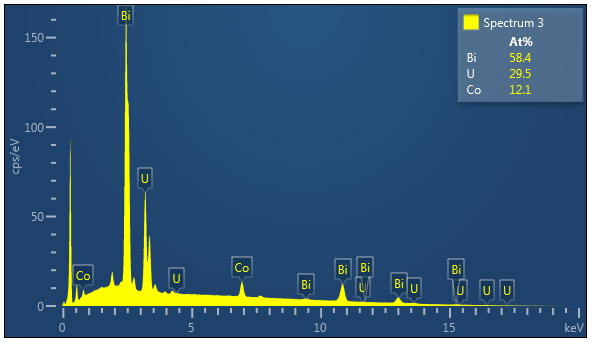


Figure S56. EDS spectrum of sample HL119O crystal 2 site 3


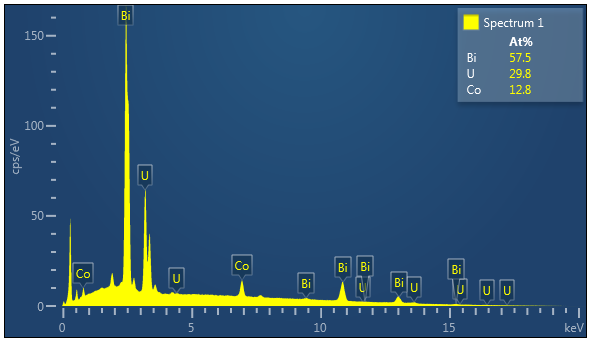


Figure S57. EDS spectrum of sample HL119O crystal 3 site 1


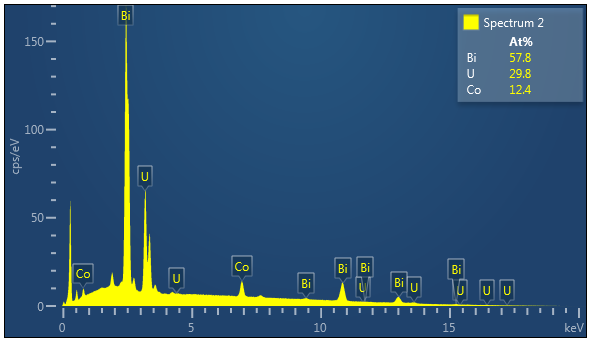


Figure S58. EDS spectrum of sample HL119O crystal 3 site 2


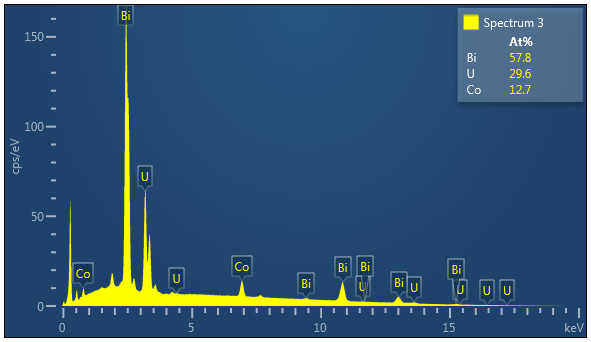


Figure S59. EDS spectrum of sample HL119O crystal 3 site 3

| 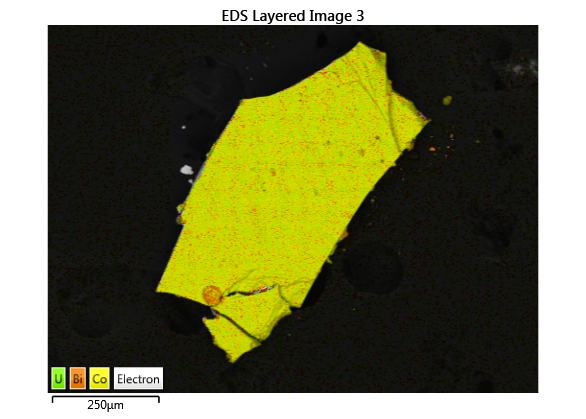 | 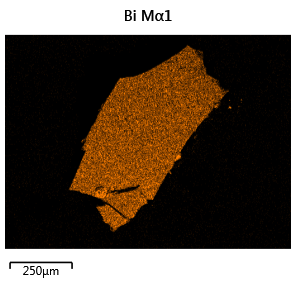 |
| --- | --- |
| 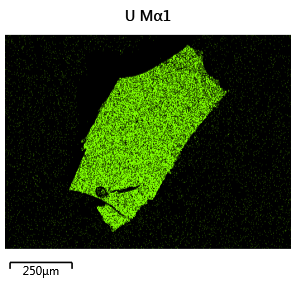 | 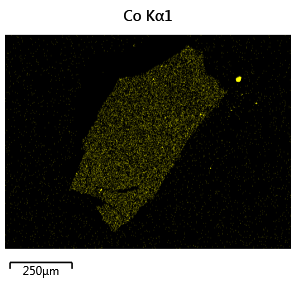 |
| Figure S60. EDS compositional mapping of a UCo*_x_*Bi_2_ single crystal obtained from a 1:1:13 U:Co:Bi flux reaction. | |

Table S29. Optimized unit cell and atomic coordinates for FM ordered UCo_1/8_Bi_2_

VASP_CONTCAR_UCo1-8Bi2_FM

1.00000000000000

8.9804913917855291 0.0122614412822862 -0.0000000000000000

0.0122614412822862 8.9804913917855114 0.0000000000000001

0.0000000000000000 -0.0000000000000001 9.0002247031440987

U Bi Co

8 16 1

Direct

0.1242559193838987 0.6340534339844522 0.2867809610457482

0.3659465660155767 0.8757440806161154 0.7132190389542600

0.8840534339844522 0.8742559193838846 0.7132190389542600

0.6255734960641195 0.6157493773338911 0.2832326534901759

0.6244265039358805 0.1342506226661032 0.2832326534901759

0.8657493773338911 0.3755734960641262 0.7167673465098379

0.3842506226661096 0.3744265039358738 0.7167673465098379

0.1257440806160947 0.1159465660155561 0.2867809610457482

0.3748696820056269 0.6251303179943667 0.0000000000000000

0.1254917900510568 0.6138870661512672 0.6495256572340340

0.3861129338487262 0.8745082099489487 0.3504743427659613

0.1250000000000000 0.8750000000000000 0.0000000000000000

0.6250000000000000 0.8750000000000000 0.9972951347539281

0.8638870661512672 0.8754917900510513 0.3504743427659613

0.6246959466752825 0.6307439090650373 0.6432402999367859

0.8751295608365373 0.6251295608365373 0.0000000000000000

0.8751303179943667 0.1248696820056261 0.0000000000000000

0.6253040533247175 0.1192560909349696 0.6432402999367859

0.8807439090650373 0.3746959466752753 0.3567597000632205

0.6250000000000000 0.3750000000000000 0.0000000000000000

0.1250000000000000 0.3750000000000000 0.0027048652460930

0.3692560909349696 0.3753040533247247 0.3567597000632205

0.1245082099489434 0.1361129338487195 0.6495256572340340

0.3748704391634619 0.1248704391634695 0.0000000000000000

0.1250000000000000 0.8750000000000000 0.5000000000000000

Table S30. Optimized unit cell and atomic coordinates for FM ordered UCo_2/8_Bi_2_

VASP_CONTCAR_UCo2-8Bi2_FM

1.00000000000000

8.9959772287238664 0.0139594314470869 0.0000000000000001

0.0139594314470868 8.9959772287238664 -0.0000000000000000

0.0000000000000000 0.0000000000000000 9.0180807432740746

U Bi Co

8 16 2

Direct

0.1227253928434772 0.6413375906905484 0.2864565749065242

0.3586624093094695 0.8772746071565043 0.7135434250934631

0.8913375906905484 0.8727253928434957 0.7135434250934631

0.6272746071565043 0.6086624093094516 0.2864565749065242

0.6227253928434957 0.1413375906905399 0.2864565749065242

0.8586624093094516 0.3772746071565349 0.7135434250934631

0.3913375906905305 0.3727253928434651 0.7135434250934631

0.1272746071565299 0.1086624093094708 0.2864565749065242

0.3750000000000000 0.6250000000000000 0.0000000000000000

0.1265395175273528 0.6101863722555021 0.6478333061142618

0.3898136277444886 0.8734604824726532 0.3521666938857442

0.1250000000000000 0.8750000000000000 0.0000000000000000

0.6250000000000000 0.8750000000000000 0.0000000000000000

0.8601863722555021 0.8765395175273468 0.3521666938857442

0.6234604824726532 0.6398136277444979 0.6478333061142618

0.8750000000000000 0.6250000000000000 0.0000000000000000

0.8750000000000000 0.1250000000000000 0.0000000000000000

0.6265395175273468 0.1101863722555132 0.6478333061142618

0.8898136277444979 0.3734604824726620 0.3521666938857442

0.6250000000000000 0.3750000000000000 0.0000000000000000

0.1250000000000000 0.3750000000000000 0.0000000000000000

0.3601863722555114 0.3765395175273380 0.3521666938857442

0.1234604824726526 0.1398136277444914 0.6478333061142618

0.3750000000000000 0.1250000000000000 0.0000000000000000

0.1250000000000000 0.8750000000000000 0.5000000000000000

0.6250000000000000 0.3750000000000000 0.5000000000000000

Table S31. Optimized unit cell and atomic coordinates for FM ordered UCo_3/8_Bi_2_

VASP_CONTCAR_UCo3-8Bi2_FM

1.00000000000000

9.0272615669839542 -0.0003225689623177 0.0000000000000000

-0.0003225689623177 9.0272615669839684 0.0000000000000000

0.0000000000000000 0.0000000000000000 8.9941305939026517

U Bi Co

8 16 3

Direct

0.1250064081161715 0.6341467609672357 0.2850186643761940

0.3690317645353395 0.8749866548740959 0.7073086155305452

0.8809682354646579 0.8750133451259041 0.7073086155305452

0.6249866548740959 0.6190317645353421 0.2926913844694792

0.6250133451259041 0.1309682354646531 0.2926913844694792

0.8658532390327643 0.3749935918838230 0.7149813356237895

0.3841467609672246 0.3750064081161770 0.7149813356237895

0.1249935918838213 0.1158532390327746 0.2850186643761940

0.3744654756371892 0.6244654756371792 -0.0000000000000000

0.1249923366000468 0.6140141956340281 0.6475862613360425

0.3782717203866658 0.8750091654338045 0.3407211184900144

0.1250000000000000 0.8750000000000000 0.9942932636486970

0.6250000000000000 0.8750000000000000 -0.0000000000000000

0.8717282796133272 0.8749908345661955 0.3407211184900144

0.6250091654338045 0.6282717203866728 0.6592788815100086

0.8755347335294126 0.6244652664705874 -0.0000000000000000

0.8755345243628208 0.1255345243628029 -0.0000000000000000

0.6249908345661955 0.1217282796133352 0.6592788815100086

0.8859858043659719 0.3750076633999497 0.3524137386639608

0.6250000000000000 0.3750000000000000 0.0057067363513110

0.1250000000000000 0.3750000000000000 -0.0000000000000000

0.3640141956340425 0.3749923366000503 0.3524137386639608

0.1250076633999453 0.1359858043659649 0.6475862613360425

0.3744652664705757 0.1255347335294126 -0.0000000000000000

0.1250000000000000 0.8750000000000000 0.5008367993219607

0.6250000000000000 0.8750000000000000 0.5000000000000000

0.6250000000000000 0.3750000000000000 0.4991632006780189

Table S32. Optimized unit cell and atomic coordinates for FM ordered UCo_4/8_Bi_2_

VASP_CONTCAR_UCo4-8Bi2_FM

1.00000000000000

9.0463772165847143 -0.0000432730659137 -0.0000000000000000

-0.0000432730659136 9.0463772165846628 0.0000000000000000

0.0000000000000000 0.0000000000000000 8.9916455854175084

U Bi Co

8 16 4

Direct

0.1250000000000000 0.6250000000000000 0.2916679913323120

0.3750000000000000 0.8750000000000000 0.7083320086676789

0.8750000000000000 0.8750000000000000 0.7083320086676789

0.6250000000000000 0.6250000000000000 0.2916679913323120

0.6250000000000000 0.1250000000000000 0.2916679913323120

0.8750000000000000 0.3750000000000000 0.7083320086676789

0.3750000000000000 0.3750000000000000 0.7083320086676789

0.1250000000000000 0.1250000000000000 0.2916679913323120

0.3750000000000000 0.6250000000000000 -0.0000000000000000

0.1250000000000000 0.6250000000000000 0.6583018508886753

0.3750000000000000 0.8750000000000000 0.3416981491113303

0.1250000000000000 0.8750000000000000 -0.0000000000000000

0.6250000000000000 0.8750000000000000 -0.0000000000000000

0.8750000000000000 0.8750000000000000 0.3416981491113303

0.6250000000000000 0.6250000000000000 0.6583018508886753

0.8750000000000000 0.6250000000000000 -0.0000000000000000

0.8750000000000000 0.1250000000000000 -0.0000000000000000

0.6250000000000000 0.1250000000000000 0.6583018508886753

0.8750000000000000 0.3750000000000000 0.3416981491113303

0.6250000000000000 0.3750000000000000 -0.0000000000000000

0.1250000000000000 0.3750000000000000 -0.0000000000000000

0.3750000000000000 0.3750000000000000 0.3416981491113303

0.1250000000000000 0.1250000000000000 0.6583018508886753

0.3750000000000000 0.1250000000000000 -0.0000000000000000

0.1250000000000000 0.8750000000000000 0.5000000000000000

0.6250000000000000 0.8750000000000000 0.5000000000000000

0.6250000000000000 0.3750000000000000 0.5000000000000000

0.1250000000000000 0.3750000000000000 0.5000000000000000

Table S33. Optimized unit cell and atomic coordinates for FM ordered UCo_5/8_Bi_2_

VASP_CONTCAR_UCo5-8Bi2_FM

1.00000000000000

9.0448034556349697 -0.0000292986549992 0.0000000000000000

-0.0000292986549993 9.0448034556349732 0.0000000000000000

0.0000000000000000 0.0000000000000000 9.0493957871142960

U Bi Co

8 16 5

Direct

0.1121178618084086 0.6249995775892998 0.2888239607500753

0.3750003736391747 0.8846878039131932 0.7061366588395590

0.8749995775892998 0.8621178618084270 0.7111760392498944

0.6378821381915730 0.6250004224107002 0.2888239607500753

0.6153121960868068 0.1249996263608253 0.2938633411604109

0.8750004224107002 0.3878821381916048 0.7111760392498944

0.3749996263608253 0.3653121960868439 0.7061366588395590

0.1346878039131758 0.1250003736391747 0.2938633411604109

0.3750000000000000 0.6250000000000000 0.9956617005016226

0.1322968314878739 0.6250002680255662 0.6526338158995781

0.3749998465224067 0.8618813721076155 0.3323368619177423

0.1245787512093970 0.8745787512094108 0.0000000000000000

0.6254212731571744 0.8745787268428256 0.0000000000000000

0.8750002680255662 0.8822968314879163 0.3473661841003863

0.6177031685120837 0.6249997319744338 0.6526338158995781

0.8750000000000000 0.6250000000000000 0.0000000000000000

0.8750000000000000 0.1250000000000000 0.0043382994983935

0.6381186278923845 0.1250001534776004 0.6676631380822604

0.8749997319744338 0.3677031685121395 0.3473661841003863

0.6254212487905892 0.3754212487906030 0.0000000000000000

0.1245787268427976 0.3754212731571953 0.0000000000000000

0.3750001534775933 0.3881186278923616 0.3323368619177423

0.1118813721076299 0.1249998465223996 0.6676631380822604

0.3750000000000000 0.1250000000000000 0.0000000000000000

0.1334093198307738 0.8834093198307721 0.5000000000000000

0.6165909996248010 0.8834090003751990 0.5000000000000000

0.6165906801692279 0.3665906801692322 0.5000000000000000

0.1334090003752007 0.3665909996248053 0.5000000000000000

0.3750000000000000 0.1250000000000000 0.5000000000000000

Table S34. Optimized unit cell and atomic coordinates for FM ordered UCo_6/8_Bi_2_

VASP_CONTCAR_UCo6-8Bi2_FM

1.00000000000000

9.0473054627045464 0.0000217140763453 -0.0000000000000000

0.0000217140763452 9.0473054627045499 -0.0000000000000000

-0.0000000000000000 -0.0000000000000000 9.1065347735342677

U Bi Co

8 16 6

Direct

0.1074617156591094 0.6249994793833764 0.2911446699544009

0.3750005206166236 0.8925382843408629 0.7088553300456207

0.8749994793833764 0.8574617156591371 0.7088553300456207

0.6425382843408629 0.6250005206166236 0.2911446699544009

0.6074617156591371 0.1249994793833693 0.2911446699544009

0.8750005206166236 0.3925382843408980 0.7088553300456207

0.3749994793833764 0.3574617156591020 0.7088553300456207

0.1425382843408914 0.1250005206166307 0.2911446699544009

0.3750000000000000 0.6250000000000000 -0.0000000000000000

0.1419358642007109 0.6249999549115586 0.6617559049346203

0.3750000450884272 0.8580641357992608 0.3382440950653735

0.1250000000000000 0.8750000000000000 -0.0000000000000000

0.6250000000000000 0.8750000000000000 -0.0000000000000000

0.8749999549115586 0.8919358642007392 0.3382440950653735

0.6080641357992608 0.6250000450884414 0.6617559049346203

0.8750000000000000 0.6250000000000000 -0.0000000000000000

0.8750000000000000 0.1250000000000000 -0.0000000000000000

0.6419358642007392 0.1249999549115657 0.6617559049346203

0.8750000450884414 0.3580641357992765 0.3382440950653735

0.6250000000000000 0.3750000000000000 -0.0000000000000000

0.1250000000000000 0.3750000000000000 -0.0000000000000000

0.3749999549115728 0.3919358642007235 0.3382440950653735

0.1080641357992893 0.1250000450884414 0.6617559049346203

0.3750000000000000 0.1250000000000000 -0.0000000000000000

0.1250000000000000 0.8750000000000000 0.5000000000000000

0.6250000000000000 0.8750000000000000 0.5000000000000000

0.8750000000000000 0.6250000000000000 0.5000000000000000

0.6250000000000000 0.3750000000000000 0.5000000000000000

0.1250000000000000 0.3750000000000000 0.5000000000000000

0.3750000000000000 0.1250000000000000 0.5000000000000000

Table S35. Optimized unit cell and atomic coordinates for FM ordered UCo_7/8_Bi_2_

VASP_CONTCAR_UCo7-8Bi2_FM

1.00000000000000

9.0638505477746598 0.0000295219038045 0.0000000000000001

0.0000295219038057 9.0638505477746616 -0.0000000000000001

0.0000000000000001 0.0000000000000001 9.0262147034660849

U Bi Co

8 16 7

Direct

0.1360449709705487 0.6250017885532841 0.3043097754243559

0.3749993158357725 0.8639465440664521 0.7098714143016820

0.8750017885532841 0.8860449709705061 0.6956902245757436

0.6139550290294939 0.6249982114467159 0.3043097754243559

0.6360534559335479 0.1250006841642062 0.2901285856983962

0.8749982114467159 0.3639550290294514 0.6956902245757436

0.3750006841642275 0.3860534559335337 0.7098714143016820

0.1139465440664521 0.1249993158358009 0.2901285856983962

0.3750000000000000 0.6250000000000000 0.0091581911387497

0.1180835003272711 0.6250006157806354 0.6784960985348807

0.3750003937872464 0.8884709540728947 0.3414422082963975

0.1252587874152657 0.8752587874152515 -0.0000000000000000

0.6247413169562430 0.8752586830437570 -0.0000000000000000

0.8750006157806354 0.8680835003272924 0.3215039014650625

0.6319164996727076 0.6249993842193646 0.6784960985348807

0.8750000000000000 0.6250000000000000 -0.0000000000000000

0.8750000000000000 0.1250000000000000 0.9908418088612291

0.6115290459271053 0.1249996062127536 0.6585577917035598

0.8749993842193646 0.3819164996727147 0.3215039014650625

0.6247412125847485 0.3747412125847556 -0.0000000000000000

0.1252586830437570 0.3747413169562714 -0.0000000000000000

0.3749996062127536 0.3615290459270982 0.3414422082963975

0.1384709540728947 0.1250003937872393 0.6585577917035598

0.3750000000000000 0.1250000000000000 -0.0000000000000000

0.3750000000000000 0.6250000000000000 0.4951357401664541

0.1136939459843192 0.8636939459843049 0.5000000000000000

0.6363082584848712 0.8636917415151288 0.5000000000000000

0.8750000000000000 0.6250000000000000 0.5000000000000000

0.8750000000000000 0.1250000000000000 0.5048642598335104

0.6363060540156951 0.3863060540156808 0.5000000000000000

0.1136917415151289 0.3863082584848498 0.5000000000000000

Table S36. Optimized unit cell and atomic coordinates for FM ordered UCo_8/8_Bi_2_

VASP_CONTCAR_UCo8-8Bi2_FM

1.00000000000000

9.0846584762744076 0.0000034068507964 0.0000000000000001

0.0000034068507973 9.0846584762744094 -0.0000000000000001

0.0000000000000001 0.0000000000000000 8.9102086677137837

U Bi Co

8 16 8

Direct

0.1250000000000000 0.6250000000000000 0.3077183765205934

0.3750000000000000 0.8750000000000000 0.6922816234794898

0.8750000000000000 0.8750000000000000 0.6922816234794898

0.6250000000000000 0.6250000000000000 0.3077183765205934

0.6250000000000000 0.1250000000000000 0.3077183765205934

0.8750000000000000 0.3750000000000000 0.6922816234794898

0.3750000000000000 0.3750000000000000 0.6922816234794898

0.1250000000000000 0.1250000000000000 0.3077183765205934

0.3750000000000000 0.6250000000000000 -0.0000000000000000

0.1250000000000000 0.6250000000000000 0.6770510171231123

0.3750000000000000 0.8750000000000000 0.3229489828768574

0.1250000000000000 0.8750000000000000 -0.0000000000000000

0.6250000000000000 0.8750000000000000 -0.0000000000000000

0.8750000000000000 0.8750000000000000 0.3229489828768574

0.6250000000000000 0.6250000000000000 0.6770510171231123

0.8750000000000000 0.6250000000000000 -0.0000000000000000

0.8750000000000000 0.1250000000000000 -0.0000000000000000

0.6250000000000000 0.1250000000000000 0.6770510171231123

0.8750000000000000 0.3750000000000000 0.3229489828768574

0.6250000000000000 0.3750000000000000 -0.0000000000000000

0.1250000000000000 0.3750000000000000 -0.0000000000000000

0.3750000000000000 0.3750000000000000 0.3229489828768574

0.1250000000000000 0.1250000000000000 0.6770510171231123

0.3750000000000000 0.1250000000000000 -0.0000000000000000

0.3750000000000000 0.6250000000000000 0.5000000000000000

0.1250000000000000 0.8750000000000000 0.5000000000000000

0.6250000000000000 0.8750000000000000 0.5000000000000000

0.8750000000000000 0.6250000000000000 0.5000000000000000

0.8750000000000000 0.1250000000000000 0.5000000000000000

0.6250000000000000 0.3750000000000000 0.5000000000000000

0.1250000000000000 0.3750000000000000 0.5000000000000000

0.3750000000000000 0.1250000000000000 0.5000000000000000

Table S37. Optimized unit cell and atomic coordinates for FM ordered UNi_1/8_Bi_2_

VASP_CONTCAR_UNi1-8Bi2_FM

1.00000000000000

8.9835032581778655 0.0147349484161425 0.0000000000000000

0.0147349484161425 8.9835032581778478 0.0000000000000001

-0.0000000000000000 -0.0000000000000001 9.0220900427950408

U Bi Ni

8 16 1

Direct

0.1241305651968791 0.6302070032314308 0.2836093863434946

0.3697929967686011 0.8758694348031353 0.7163906136565120

0.8802070032314308 0.8741305651968647 0.7163906136565120

0.6255487141115700 0.6168387054184802 0.2834730891235204

0.6244512858884300 0.1331612945815117 0.2834730891235204

0.8668387054184802 0.3755487141115759 0.7165269108764900

0.3831612945815184 0.3744512858884241 0.7165269108764900

0.1258694348031136 0.1197929967685794 0.2836093863434946

0.3749814934541439 0.6250185065458501 -0.0000000000000000

0.1253654395662388 0.6155222284315442 0.6495222598165360

0.3844777715684489 0.8746345604337689 0.3504777401834591

0.1250000000000000 0.8750000000000000 -0.0000000000000000

0.6250000000000000 0.8750000000000000 0.9996021699045972

0.8655222284315442 0.8753654395662311 0.3504777401834591

0.6246122116427787 0.6298487785925727 0.6432013743449596

0.8749129402048279 0.6249129402048279 -0.0000000000000000

0.8750185065458501 0.1249814934541438 -0.0000000000000000

0.6253877883572213 0.1201512214074370 0.6432013743449596

0.8798487785925727 0.3746122116427747 0.3567986256550497

0.6250000000000000 0.3750000000000000 -0.0000000000000000

0.1250000000000000 0.3750000000000000 0.0003978300954255

0.3701512214074367 0.3753877883572253 0.3567986256550497

0.1246345604337611 0.1344777715684422 0.6495222598165360

0.3750870597951706 0.1250870597951781 -0.0000000000000000

0.1250000000000000 0.8750000000000000 0.5000000000000000

Table S38. Optimized unit cell and atomic coordinates for FM ordered UNi_2/8_Bi_2_

VASP_CONTCAR_UNi2-8Bi2_FM

1.00000000000000

8.9967462435160535 -0.0010466883014568 0.0000000000000000

-0.0010466883014569 8.9967462435160375 -0.0000000000000000

-0.0000000000000000 0.0000000000000000 9.0693767235340026

U Bi Ni

8 16 2

Direct

0.1249769775387649 0.6365207719416086 0.2827547773985427

0.3634792280583941 0.8750230224612189 0.7172452226014465

0.8865207719416086 0.8749769775387811 0.7172452226014465

0.6250230224612189 0.6134792280583914 0.2827547773985427

0.6249769775387811 0.1365207719416099 0.2827547773985427

0.8634792280583914 0.3750230224612382 0.7172452226014465

0.3865207719416059 0.3749769775387618 0.7172452226014465

0.1250230224612378 0.1134792280583959 0.2827547773985427

0.3750000000000000 0.6250000000000000 0.0000000000000000

0.1249788171056911 0.6129113205043235 0.6478044998351140

0.3870886794956885 0.8750211828943076 0.3521955001648866

0.1250000000000000 0.8750000000000000 0.0000000000000000

0.6250000000000000 0.8750000000000000 0.0000000000000000

0.8629113205043235 0.8749788171056924 0.3521955001648866

0.6250211828943076 0.6370886794956765 0.6478044998351140

0.8750000000000000 0.6250000000000000 0.0000000000000000

0.8750000000000000 0.1250000000000000 0.0000000000000000

0.6249788171056924 0.1129113205043136 0.6478044998351140

0.8870886794956765 0.3750211828943084 0.3521955001648866

0.6250000000000000 0.3750000000000000 0.0000000000000000

0.1250000000000000 0.3750000000000000 0.0000000000000000

0.3629113205043115 0.3749788171056916 0.3521955001648866

0.1250211828943073 0.1370886794956925 0.6478044998351140

0.3750000000000000 0.1250000000000000 0.0000000000000000

0.1250000000000000 0.8750000000000000 0.5000000000000000

0.6250000000000000 0.3750000000000000 0.5000000000000000

Table S39. Optimized unit cell and atomic coordinates for FM ordered UNi_3/8_Bi_2_

VASP_CONTCAR_UNi3-8Bi2_FM

1.00000000000000

9.0282389855866523 -0.0101842345126839 0.0000000000000000

-0.0101842345126839 9.0282389855866629 0.0000000000000000

0.0000000000000000 0.0000000000000000 9.0728964488068016

U Bi Ni

8 16 3

Direct

0.1259761180466417 0.6296269837513880 0.2824079543670366

0.3695122825663658 0.8736999491521209 0.7153536157163359

0.8804877174336349 0.8763000508478791 0.7153536157163359

0.6236999491521209 0.6195122825663651 0.2846463842836861

0.6263000508478791 0.1304877174336275 0.2846463842836861

0.8703730162486120 0.3740238819533523 0.7175920456329508

0.3796269837513885 0.3759761180466477 0.7175920456329508

0.1240238819533512 0.1203730162486120 0.2824079543670366

0.3745423101627107 0.6245423101626966 0.0000000000000000

0.1241830733395532 0.6158295292240844 0.6474936867208886

0.3770670115632788 0.8749435362612616 0.3427892701085633

0.1250000000000000 0.8750000000000000 -0.0023597223645331

0.6250000000000000 0.8750000000000000 0.0000000000000000

0.8729329884367131 0.8750564637387384 0.3427892701085633

0.6249435362612616 0.6270670115632869 0.6572107298914506

0.8754388986694784 0.6245611013305216 0.0000000000000000

0.8754576898373034 0.1254576898372826 0.0000000000000000

0.6250564637387384 0.1229329884367205 0.6572107298914506

0.8841704707759156 0.3758169266604392 0.3525063132791195

0.6250000000000000 0.3750000000000000 1.0023597223645391

0.1250000000000000 0.3750000000000000 0.0000000000000000

0.3658295292240915 0.3741830733395608 0.3525063132791195

0.1258169266604398 0.1341704707759149 0.6474936867208886

0.3745611013305064 0.1254388986694872 0.0000000000000000

0.1250000000000000 0.8750000000000000 0.5024988362840752

0.6250000000000000 0.8750000000000000 0.5000000000000000

0.6250000000000000 0.3750000000000000 0.4975011637159035

Table S40. Optimized unit cell and atomic coordinates for FM ordered UNi_4/8_Bi_2_

VASP_CONTCAR_UNi4-8Bi2_FM

1.00000000000000

9.0470433589760848 0.0000198037834585 -0.0000000000000000

0.0000198037834585 9.0470433589760528 0.0000000000000000

0.0000000000000000 -0.0000000000000000 9.1061114612028025

U Bi Ni

8 16 4

Direct

0.1250000000000000 0.6250000000000000 0.2838080327127045

0.3750000000000000 0.8750000000000000 0.7161919672872896

0.8750000000000000 0.8750000000000000 0.7161919672872896

0.6250000000000000 0.6250000000000000 0.2838080327127046

0.6250000000000000 0.1250000000000000 0.2838080327127045

0.8750000000000000 0.3750000000000000 0.7161919672872896

0.3750000000000000 0.3750000000000000 0.7161919672872896

0.1250000000000000 0.1250000000000000 0.2838080327127045

0.3750000000000000 0.6250000000000000 0.0000000000000000

0.1250000000000000 0.6250000000000000 0.6559172173334600

0.3750000000000000 0.8750000000000000 0.3440827826665394

0.1250000000000000 0.8750000000000000 0.0000000000000000

0.6250000000000000 0.8750000000000000 0.0000000000000000

0.8750000000000000 0.8750000000000000 0.3440827826665394

0.6250000000000000 0.6250000000000000 0.6559172173334600

0.8750000000000000 0.6250000000000000 0.0000000000000000

0.8750000000000000 0.1250000000000000 0.0000000000000000

0.6250000000000000 0.1250000000000000 0.6559172173334600

0.8750000000000000 0.3750000000000000 0.3440827826665394

0.6250000000000000 0.3750000000000000 0.0000000000000000

0.1250000000000000 0.3750000000000000 0.0000000000000000

0.3750000000000000 0.3750000000000000 0.3440827826665394

0.1250000000000000 0.1250000000000000 0.6559172173334600

0.3750000000000000 0.1250000000000000 0.0000000000000000

0.1250000000000000 0.8750000000000000 0.5000000000000000

0.6250000000000000 0.8750000000000000 0.5000000000000000

0.6250000000000000 0.3750000000000000 0.5000000000000000

0.1250000000000000 0.3750000000000000 0.5000000000000000

Table S41. Optimized unit cell and atomic coordinates for FM ordered UNi_5/8_Bi_2_

VASP_CONTCAR_UNi5-8Bi2_FM

1.00000000000000

9.0663394676241218 0.0012557853780020 0.0000000000000000

0.0012557853780019 9.0663394676241218 0.0000000000000000

0.0000000000000000 0.0000000000000000 9.1101683789398660

U Bi Ni

8 16 5

Direct

0.1152921476679604 0.6252093614075133 0.2841313080782626

0.3748061879084687 0.8821905665532884 0.7148496368917764

0.8752093614075133 0.8652921476679750 0.7158686919217077

0.6347078523320250 0.6247906385924867 0.2841313080782626

0.6178094334467116 0.1251938120915320 0.2851503631082002

0.8747906385924867 0.3847078523320467 0.7158686919217077

0.3751938120915313 0.3678094334467503 0.7148496368917764

0.1321905665532748 0.1248061879084686 0.2851503631082002

0.3750000000000000 0.6250000000000000 0.0000516322305970

0.1299442824975050 0.6248872254190176 0.6562813476012053

0.3750997514804780 0.8665728390079600 0.3391916275509493

0.1248146652660964 0.8748146652661100 0.0000000000000000

0.6251798889275383 0.8748201110724617 0.0000000000000000

0.8748872254190176 0.8799442824975480 0.3437186523987580

0.6200557175024520 0.6251127745809824 0.6562813476012053

0.8750000000000000 0.6250000000000000 0.0000000000000000

0.8750000000000000 0.1250000000000000 0.9999483677694104

0.6334271609920400 0.1249002485195276 0.6608083724490342

0.8751127745809824 0.3700557175025085 0.3437186523987580

0.6251853347338900 0.3751853347339051 0.0000000000000000

0.1248201110724331 0.3751798889275667 0.0000000000000000

0.3749002485195220 0.3834271609920135 0.3391916275509493

0.1165728390079799 0.1250997514804722 0.6608083724490342

0.3750000000000000 0.1250000000000000 0.0000000000000000

0.1248858905063915 0.8748858905063766 0.5000000000000000

0.6250455116637480 0.8749544883362520 0.5000000000000000

0.6251141094936234 0.3751141094936235 0.5000000000000000

0.1249544883362680 0.3750455116637392 0.5000000000000000

0.3750000000000000 0.1250000000000000 0.5000000000000000

Table S42. Optimized unit cell and atomic coordinates for FM ordered Ni_6/8_Bi_2_

VASP_CONTCAR_UNi6-8Bi2_FM

1.00000000000000

9.0851193017923784 -0.0000284142374150 0.0000000000000000

-0.0000284142374151 9.0851193017923819 -0.0000000000000000

0.0000000000000000 -0.0000000000000000 9.1198566104415715

U Bi Ni

8 16 6

Direct

0.1108999997609905 0.6250656920784083 0.2855960347821356

0.3749343079215829 0.8891000002389855 0.7144039652178746

0.8750656920784083 0.8608999997610145 0.7144039652178746

0.6391000002389855 0.6249343079215917 0.2855960347821356

0.6108999997610145 0.1250656920784096 0.2855960347821356

0.8749343079215917 0.3891000002390144 0.7144039652178746

0.3750656920784171 0.3608999997609856 0.7144039652178746

0.1391000002390091 0.1249343079215918 0.2855960347821356

0.3750000000000000 0.6250000000000000 -0.0000000000000000

0.1368991305858325 0.6249571812640647 0.6605086824931184

0.3750428187359273 0.8631008694141407 0.3394913175068695

0.1250000000000000 0.8750000000000000 -0.0000000000000000

0.6250000000000000 0.8750000000000000 -0.0000000000000000

0.8749571812640647 0.8868991305858593 0.3394913175068695

0.6131008694141407 0.6250428187359353 0.6605086824931184

0.8750000000000000 0.6250000000000000 -0.0000000000000000

0.8750000000000000 0.1250000000000000 -0.0000000000000000

0.6368991305858593 0.1249571812640654 0.6605086824931184

0.8750428187359353 0.3631008694141528 0.3394913175068695

0.6250000000000000 0.3750000000000000 -0.0000000000000000

0.1250000000000000 0.3750000000000000 -0.0000000000000000

0.3749571812640727 0.3868991305858472 0.3394913175068695

0.1131008694141668 0.1250428187359418 0.6605086824931184

0.3750000000000000 0.1250000000000000 -0.0000000000000000

0.1250000000000000 0.8750000000000000 0.5000000000000000

0.6250000000000000 0.8750000000000000 0.5000000000000000

0.8750000000000000 0.6250000000000000 0.5000000000000000

0.6250000000000000 0.3750000000000000 0.5000000000000000

0.1250000000000000 0.3750000000000000 0.5000000000000000

0.3750000000000000 0.1250000000000000 0.5000000000000000

Table S43. Optimized unit cell and atomic coordinates for FM ordered UNi_7/8_Bi_2_

VASP_CONTCAR_UNi7-8Bi2_FM

1.00000000000000

9.1061079008983228 0.0000184947184326 0.0000000000000001

0.0000184947184335 9.1061079008983139 -0.0000000000000001

0.0000000000000001 0.0000000000000001 9.1156793209172147

U Bi Ni

8 16 7

Direct

0.1331890931736096 0.6249919162450408 0.2881830025830289

0.3750100326408327 0.8673842508139276 0.7141115850774264

0.8749919162450408 0.8831890931736117 0.7118169974170555

0.6168109068263883 0.6250080837549592 0.2881830025830289

0.6326157491860724 0.1249899673591661 0.2858884149226588

0.8750080837549592 0.3668109068263902 0.7118169974170555

0.3749899673591673 0.3826157491860713 0.7141115850774264

0.1173842508139279 0.1250100326408339 0.2858884149226588

0.3750000000000000 0.6250000000000000 0.0013638082981789

0.1211314448191417 0.6250053221576669 0.6675238465649850

0.3749897840814496 0.8833631622489682 0.3390171769599346

0.1253273791679134 0.8753273791679114 0.0000000000000000

0.6246724337648101 0.8753275662351899 0.0000000000000000

0.8750053221576669 0.8711314448191426 0.3324761534349754

0.6288685551808574 0.6249946778423331 0.6675238465649850

0.8750000000000000 0.6250000000000000 0.0000000000000000

0.8750000000000000 0.1250000000000000 -0.0013638082981789

0.6166368377510318 0.1250102159185521 0.6609828230400240

0.8749946778423331 0.3788685551808589 0.3324761534349754

0.6246726208320886 0.3746726208320862 0.0000000000000000

0.1253275662351893 0.3746724337648106 0.0000000000000000

0.3750102159185504 0.3666368377510298 0.3390171769599346

0.1333631622489686 0.1249897840814481 0.6609828230400240

0.3750000000000000 0.1250000000000000 0.0000000000000000

0.3750000000000000 0.6250000000000000 0.4987475659094926

0.1251147861490045 0.8751147861489998 0.5000000000000000

0.6248748923864271 0.8751251076135729 0.5000000000000000

0.8750000000000000 0.6250000000000000 0.5000000000000000

0.8750000000000000 0.1250000000000000 0.5012524340905056

0.6248852138510002 0.3748852138509963 0.5000000000000000

0.1251251076135739 0.3748748923864249 0.5000000000000000

Table S44. Optimized unit cell and atomic coordinates for FM ordered UNi_8/8_Bi_2_

VASP_CONTCAR_UNi8-8Bi2_FM

1.00000000000000

9.1311455089035238 -0.0015534575341337 0.0000000000000001

-0.0015534575341327 9.1311455089035256 -0.0000000000000001

0.0000000000000001 0.0000000000000001 9.0953587816798471

U Bi Ni

8 16 8

Direct

0.1250000000000000 0.6250000000000000 0.2900426402842304

0.3750000000000000 0.8750000000000000 0.7099573597158534

0.8750000000000000 0.8750000000000000 0.7099573597158534

0.6250000000000000 0.6250000000000000 0.2900426402842304

0.6250000000000000 0.1250000000000000 0.2900426402842304

0.8750000000000000 0.3750000000000000 0.7099573597158534

0.3750000000000000 0.3750000000000000 0.7099573597158534

0.1250000000000000 0.1250000000000000 0.2900426402842304

0.3750000000000000 0.6250000000000000 0.0000000000000000

0.1250000000000000 0.6250000000000000 0.6685853845873495

0.3750000000000000 0.8750000000000000 0.3314146154126114

0.1250000000000000 0.8750000000000000 0.0000000000000000

0.6250000000000000 0.8750000000000000 0.0000000000000000

0.8750000000000000 0.8750000000000000 0.3314146154126114

0.6250000000000000 0.6250000000000000 0.6685853845873495

0.8750000000000000 0.6250000000000000 0.0000000000000000

0.8750000000000000 0.1250000000000000 0.0000000000000000

0.6250000000000000 0.1250000000000000 0.6685853845873495

0.8750000000000000 0.3750000000000000 0.3314146154126114

0.6250000000000000 0.3750000000000000 0.0000000000000000

0.1250000000000000 0.3750000000000000 0.0000000000000000

0.3750000000000000 0.3750000000000000 0.3314146154126114

0.1250000000000000 0.1250000000000000 0.6685853845873495

0.3750000000000000 0.1250000000000000 0.0000000000000000

0.3750000000000000 0.6250000000000000 0.5000000000000000

0.1250000000000000 0.8750000000000000 0.5000000000000000

0.6250000000000000 0.8750000000000000 0.5000000000000000

0.8750000000000000 0.6250000000000000 0.5000000000000000

0.8750000000000000 0.1250000000000000 0.5000000000000000

0.6250000000000000 0.3750000000000000 0.5000000000000000

0.1250000000000000 0.3750000000000000 0.5000000000000000

0.3750000000000000 0.1250000000000000 0.5000000000000000

Table S45. Optimized unit cell and atomic coordinates for AFM ordered UNi_1/8_Bi_2_

VASP_CONTCAR_UNi1-8Bi2_AFM

1.00000000000000

9.0036958021796316 -0.0042466664526828 -0.0000237399170830

-0.0042466664526828 9.0036958021796298 0.0000237399170831

-0.0000236543144461 0.0000236543144460 9.0095541551352909

U Bi Ni

8 16 1

Direct

0.1253884639520833 0.6297157186673112 0.2819830322066931

0.3702871827085970 0.8746094997292405 0.7180165371226578

0.8797157186673112 0.8753884639520828 0.7180169677933192

0.6248871716739778 0.6173282128743929 0.2831679104703365

0.6251118210349758 0.1326748847530483 0.2831676075388190

0.8673282128743929 0.3748871716739740 0.7168320895296697

0.3826748847530554 0.3751118210349758 0.7168323924611910

0.1246094997292404 0.1202871827085763 0.2819834628773551

0.3750701008489993 0.6249314415142380 0.0000012852512411

0.1250818223427628 0.6164739794661109 0.6480466167817157

0.3835218550505616 0.8749167667196327 0.3519516605035830

0.1249996538042823 0.8749996538042846 -0.0000000000000000

0.6249998458150015 0.8749980825224921 0.0011931785160142

0.8664739794661109 0.8750818223427612 0.3519533832182847

0.6252760064103334 0.6288853794247884 0.6433220359699543

0.8749272854100363 0.6249272854100363 -0.0000000000000000

0.8749314415142380 0.1250701008489999 -0.0000012852512411

0.6247346007538193 0.1211111577315048 0.6433234392091893

0.8788853794247884 0.3752760064103282 0.3566779640300570

0.6250009721706619 0.3750009721706579 -0.0000000000000000

0.1249980825224917 0.3749998458150013 0.9988068214839928

0.3711111577315073 0.3747346007538263 0.3566765607908203

0.1249167667196348 0.1335218550505539 0.6480483394964143

0.3750722186187544 0.1250722186187618 -0.0000000000000000

0.1249958760024267 0.8749958760024220 0.5000000000000000

Table S46. Optimized unit cell and atomic coordinates for AFM ordered UNi_2/8_Bi_2_

VASP_CONTCAR_UNi2-8Bi2_AFM

1.00000000000000

9.0135359745805470 -0.0004490281317013 0.0000000000000000

-0.0004490281317014 9.0135359745805488 0.0000000000000000

0.0000000000000000 0.0000000000000000 9.0653797487709156

U Bi Ni

8 16 2

Direct

0.1249854605277150 0.6355024276248230 0.2814637398933696

0.3644969895799811 0.8750141998193516 0.7185355448030473

0.8855024276248230 0.8749854605277220 0.7185362601066317

0.6250145394722780 0.6144975723751770 0.2814637398933696

0.6249858001806484 0.1355030104200254 0.2814644551969534

0.8644975723751770 0.3750145394722851 0.7185362601066317

0.3855030104200189 0.3749858001806416 0.7185355448030473

0.1250141998193591 0.1144969895799816 0.2814644551969534

0.3750000000000000 0.6250000000000000 0.0000008585358185

0.1250665743188494 0.6137441006198000 0.6459405969971480

0.3862551221272302 0.8749297413886747 0.3540585920121595

0.1249995213612417 0.8749995213612413 0.0000000000000000

0.6250002494991388 0.8749997505008612 0.0000000000000000

0.8637441006198000 0.8750665743188512 0.3540594030028523

0.6249334256811488 0.6362558993802000 0.6459405969971480

0.8750000000000000 0.6250000000000000 0.0000000000000000

0.8750000000000000 0.1250000000000000 -0.0000008585358185

0.6250702586113253 0.1137448778727768 0.6459414079878428

0.8862558993802000 0.3749334256811512 0.3540594030028523

0.6250004786387587 0.3750004786387576 0.0000000000000000

0.1249997505008595 0.3750002494991404 0.0000000000000000

0.3637448778727698 0.3750702586113235 0.3540585920121595

0.1249297413886763 0.1362551221272302 0.6459414079878428

0.3750000000000000 0.1250000000000000 0.0000000000000000

0.1249992925553092 0.8749992925553098 0.5000000000000000

0.6250007074446902 0.3750007074446925 0.5000000000000000

Table S47. Optimized unit cell and atomic coordinates for AFM ordered UNi_3/8_Bi_2_

VASP_CONTCAR_UNi3-8Bi2_AFM

1.00000000000000

9.0396139113200977 0.0028281567040096 -0.0000402012777857

0.0028281567040096 9.0396139113201084 0.0000402012777857

-0.0000404401869127 0.0000404401869127 9.0819034384872239

U Bi Ni

8 16 3

Direct

0.1250133023401140 0.6290062585134654 0.2807630134883427

0.3694533684142582 0.8747177866315061 0.7161628834355964

0.8805441485716189 0.8752827305035711 0.7161624302825781

0.6247177866315061 0.6194533684142516 0.2838371165644207

0.6252827305035711 0.1305441485716112 0.2838375697174401

0.8709952611732218 0.3749878881955133 0.7192364809996378

0.3790062585134663 0.3750133023401239 0.7192369865116408

0.1249878881955122 0.1209952611732217 0.2807635190003460

0.3744694879943620 0.6244694879943518 -0.0000000000000000

0.1249892267934467 0.6155106517894949 0.6455584859871856

0.3761869618338488 0.8750579188601560 0.3455031550632292

0.1250007105839911 0.8749997893688014 -0.0012494332139985

0.6249994099213866 0.8749994099213866 -0.0000000000000000

0.8738137753474293 0.8749390182165762 0.3455015729159325

0.6250579188601560 0.6261869618338509 0.6544968449367853

0.8754493720948608 0.6245503856008716 0.0000012032915356

0.8755311231087232 0.1255311231087063 -0.0000000000000000

0.6249390182165762 0.1238137753474358 0.6544984270840736

0.8844890387557207 0.3750134089063377 0.3544406094832392

0.6249997893688014 0.3750007105839925 1.0012494332140123

0.1250006254259182 0.3750006254259181 -0.0000000000000000

0.3655106517895070 0.3749892267934505 0.3544415140128237

0.1250134089063379 0.1344890387557229 0.6455593905167633

0.3745503856008628 0.1254493720948714 -0.0000012032915356

0.1250001928885389 0.8749993839666329 0.5045507708298972

0.6249987741996202 0.8749987741996202 0.5000000000000000

0.6249993839666329 0.3750001928885375 0.4954492291700872

Table S48. Optimized unit cell and atomic coordinates for AFM ordered UNi_4/8_Bi_2_

VASP_CONTCAR_UNi4-8Bi2_AFM

1.00000000000000

9.0614800222629448 0.0061558762771906 -0.0000000514673048

0.0061558762771907 9.0614800222629128 0.0000000514673048

-0.0000001152406082 0.0000001152406082 9.1106345399968038

U Bi Ni

8 16 4

Direct

0.1250000199226237 0.6249999734389794 0.2834181347651438

0.3749999734389746 0.8750000199226221 0.7165818652348548

0.8749999734389794 0.8750000199226221 0.7165818652348548

0.6250000199226221 0.6249999734389794 0.2834181347651438

0.6250000199226221 0.1249999734389758 0.2834181347651438

0.8749999734389794 0.3750000199226232 0.7165818652348548

0.3749999734389746 0.3750000199226232 0.7165818652348548

0.1250000199226237 0.1249999734389758 0.2834181347651438

0.3749999964927511 0.6249999964927514 -0.0000000000000000

0.1249999690437947 0.6250000201271269 0.6540388416529070

0.3750000201271270 0.8749999690438002 0.3459611583470958

0.1249999950165488 0.8749999950165467 -0.0000000000000000

0.6249999950165467 0.8749999950165467 -0.0000000000000000

0.8750000201271269 0.8749999690438002 0.3459611583470958

0.6249999690438002 0.6250000201271269 0.6540388416529070

0.8749999964927514 0.6249999964927514 -0.0000000000000000

0.8749999964927514 0.1249999964927516 -0.0000000000000000

0.6249999690438002 0.1250000201271273 0.6540388416529070

0.8750000201271269 0.3749999690437928 0.3459611583470958

0.6249999950165467 0.3749999950165480 -0.0000000000000000

0.1249999950165488 0.3749999950165480 -0.0000000000000000

0.3750000201271270 0.3749999690437928 0.3459611583470958

0.1249999690437947 0.1250000201271273 0.6540388416529070

0.3749999964927511 0.1249999964927516 -0.0000000000000000

0.1250000259581768 0.8750000259581827 0.5000000000000000

0.6250000259581827 0.8750000259581827 0.5000000000000000

0.6250000259581827 0.3750000259581763 0.5000000000000000

0.1250000259581768 0.3750000259581763 0.5000000000000000

Table S49. Optimized unit cell and atomic coordinates for AFM ordered UNi_5/8_Bi_2_

VASP_CONTCAR_UNi5-8Bi2_AFM

1.00000000000000

9.0803715409201082 0.0078517801694887 -0.0000254137999800

0.0078517801694886 9.0803715409201082 0.0000254137999800

-0.0000255956610985 0.0000255956610985 9.1117272224503765

U Bi Ni

8 16 5

Direct

0.1173849612148257 0.6248035904964864 0.2842645818806536

0.3750739630533586 0.8807589090752476 0.7153259250526078

0.8748035904964864 0.8673849612148399 0.7157354181193173

0.6326165546673456 0.6251957504722297 0.2842659287297241

0.6192411815339627 0.1249255305330402 0.2846726946354670

0.8751957504722297 0.3826165546673668 0.7157340712702446

0.3749255305330402 0.3692411815340075 0.7153273053645111

0.1307589090752319 0.1250739630533586 0.2846740749473714

0.3749967758025057 0.6249998955607307 0.0001813421719526

0.1290303976563202 0.6250937400093507 0.6542395318049292

0.3749961101713070 0.8668013262806931 0.3415697715520870

0.1247296970140026 0.8747296970140181 -0.0000000000000000

0.6251221899608260 0.8748810722830693 -0.0000040823780392

0.8750937400093507 0.8790303976563600 0.3457604681950345

0.6209697608581791 0.6249183985761200 0.6542451758138144

0.8750037686110823 0.6250037686110823 -0.0000000000000000

0.8749998955607307 0.1249967758025063 0.9998186578280527

0.6331991829100020 0.1249930814629417 0.6584293453292130

0.8749183985761200 0.3709697608582372 0.3457548241861514

0.6252666280863010 0.3752666280863154 -0.0000000000000000

0.1248810722830393 0.3751221899608531 0.0000040823780392

0.3749930814629358 0.3831991829099752 0.3415706546707697

0.1168013262807164 0.1249961101712995 0.6584302284478996

0.3749998300573354 0.1249998300573359 -0.0000000000000000

0.1249216957410443 0.8749216957410302 0.5000000000000000

0.6249379711870910 0.8750646344037700 0.5000039617341016

0.6250729345695559 0.3750729345695532 0.5000000000000000

0.1250646344037856 0.3749379711870854 0.4999960382658972

0.3750004677512293 0.1250004677512289 0.5000000000000000

Table S50. Optimized unit cell and atomic coordinates for AFM ordered UNi_6/8_Bi_2_

VASP_CONTCAR_UNi6-8Bi2_AFM

1.00000000000000

9.0968407963508362 0.0111853147685582 -0.0000068828457800

0.0111853501371890 9.0967424620944612 0.0000216342762163

-0.0000073066549392 0.0000222055594636 9.1256415182848265

U Bi Ni

8 16 6

Direct

0.1126009559205500 0.6248540164933926 0.2854723357108491

0.3751467061027768 0.8874000075387031 0.7145276945493045

0.8748537911831917 0.8625976386142237 0.7145260108134852

0.6373994603512724 0.6251469693085796 0.2854730349723933

0.6125979799917916 0.1248540312240481 0.2854754243022098

0.8751463990293044 0.3874011135948659 0.7145271158517396

0.3748533545479283 0.3625988527735161 0.7145265118796441

0.1374007072678411 0.1251466380970235 0.2854740114141794

0.3750000302945404 0.6250008232567353 0.0000015917360880

0.1365111156637319 0.6250165528703893 0.6580773562167945

0.3749831562959218 0.8634869341570500 0.3419217629276083

0.1250010675772610 0.8749997208140931 0.0000022006033484

0.6249990727053242 0.8749996032469505 0.0000018528460917

0.8750164529322197 0.8865089211164322 0.3419204741382714

0.6134897159815428 0.6249839756353838 0.6580774209893936

0.8750002171218794 0.6249996121779755 0.0000036951518182

0.8750002144282683 0.1250008691802702 0.0000016764943164

0.6365088564353799 0.1250157927670930 0.6580794802060793

0.8749839304910297 0.3634909793593213 0.3419211385688473

0.6250001967297346 0.3750011814041583 0.0000018308469618

0.1249996887689533 0.3750007610817251 0.0000022440395052

0.3750157356799837 0.3865125510963062 0.3419209628409185

0.1134907508494529 0.1249832530854593 0.6580781032258440

0.3749996296108086 0.1249995213549935 0.0000009411903619

0.1250022173725135 0.8749998116773635 0.4999971046842754

0.6249980735936524 0.8750001821110988 0.4999975793328925

0.8750012330658585 0.6249986185235239 0.4999952758238415

0.6250004726077194 0.3750015577283601 0.4999971069073686

0.1249996753014296 0.3750017718600064 0.4999969454219166

0.3749991420981488 0.1249977378509679 0.5000011163136819

Table S51. Optimized unit cell and atomic coordinates for AFM ordered UNi_7/8_Bi_2_

VASP_CONTCAR_UNi7-8Bi2_AFM

1.00000000000000

9.1201504023973872 0.0159808410606116 0.0000163219029670

0.0159808410606116 9.1201504023973889 -0.0000163219029670

0.0000169387926051 -0.0000169387926051 9.1222816994540885

U Bi Ni

8 16 7

Direct

0.1317553919030061 0.6253464564166241 0.2879654410951686

0.3751718655436000 0.8689584780525414 0.7140951173489163

0.8753464564166241 0.8817553919029891 0.7120345589049148

0.6182446806960258 0.6246537265475189 0.2879645656048002

0.6310410666312432 0.1248282725364162 0.2859035948043527

0.8746537265475189 0.3682446806960317 0.7120354343952869

0.3748282725364149 0.3810410666312435 0.7140964051957321

0.1189584780525255 0.1251718655436017 0.2859048826511800

0.3750003026885968 0.6249998953741180 1.0012404043599450

0.1220563091872159 0.6251449949090404 0.6647602091091395

0.3750815648770116 0.8835763265186798 0.3418674712356097

0.1254624929361959 0.8754624929362003 0.0000000000000000

0.6247295669629473 0.8752704787214352 -0.0000004765545758

0.8751449949090404 0.8720563091872212 0.3352397908908304

0.6279443735914869 0.6248553725670823 0.6647588760878148

0.8749999037279833 0.6249999037279833 0.0000000000000000

0.8749998953741180 0.1250003026885955 -0.0012404043599529

0.6164222843281910 0.1249177745171510 0.6581324155920388

0.8748553725670823 0.3779443735914790 0.3352411239121543

0.6245375188465738 0.3745375188465780 0.0000000000000000

0.1252704787214371 0.3747295669629506 1.0000004765545569

0.3749177745171535 0.3664222843281867 0.3418675844079285

0.1335763265186781 0.1250815648770125 0.6581325287643578

0.3749998570740193 0.1249998570740180 0.0000000000000000

0.3750003435241583 0.6249984881173783 0.4974903803647434

0.1254888245020346 0.8754888245020335 0.5000000000000000

0.6251961143651813 0.8748054216367380 0.4999992002895587

0.8750006182442664 0.6250006182442664 0.5000000000000000

0.8749984881173783 0.1250003435241652 0.5025096196352505

0.6245112344555473 0.3745112344555747 0.5000000000000000

0.1248054216367518 0.3751961143651845 0.5000007997104403

Table S52. Optimized unit cell and atomic coordinates for AFM ordered UNi_8/8_Bi_2_

VASP_CONTCAR_UNi8-8Bi2_AFM

1.00000000000000

9.1437133012679297 0.0209853636641798 0.0000000140774272

0.0209853636641808 9.1437133012679315 -0.0000000140774272

0.0000001749739844 -0.0000001749739842 9.1209175696753704

U Bi Ni

8 16 8

Direct

0.1249999837695793 0.6250000162304192 0.2890297717493809

0.3750000162304211 0.8749999837695808 0.7109702282507062

0.8750000162304192 0.8749999837695808 0.7109702282507062

0.6249999837695808 0.6250000162304192 0.2890297717493809

0.6249999837695808 0.1250000162304205 0.2890297717493809

0.8750000162304192 0.3749999837695789 0.7109702282507062

0.3750000162304211 0.3749999837695789 0.7109702282507062

0.1249999837695793 0.1250000162304205 0.2890297717493809

0.3750000004136494 0.6250000004136513 -0.0000000000000000

0.1250000204857702 0.6249999795142273 0.6647121284110037

0.3749999795142299 0.8750000204857727 0.3352878715889559

0.1249999995863504 0.8749999995863487 -0.0000000000000000

0.6249999995863487 0.8749999995863487 -0.0000000000000000

0.8749999795142273 0.8750000204857727 0.3352878715889559

0.6250000204857727 0.6249999795142273 0.6647121284110037

0.8750000004136513 0.6250000004136513 -0.0000000000000000

0.8750000004136513 0.1250000004136497 -0.0000000000000000

0.6250000204857727 0.1249999795142299 0.6647121284110037

0.8749999795142273 0.3750000204857701 0.3352878715889559

0.6249999995863487 0.3749999995863506 -0.0000000000000000

0.1249999995863504 0.3749999995863506 -0.0000000000000000

0.3749999795142299 0.3750000204857701 0.3352878715889559

0.1250000204857702 0.1249999795142299 0.6647121284110037

0.3750000004136494 0.1250000004136497 -0.0000000000000000

0.3750000308687278 0.6250000308687290 0.5000000000000000

0.1249999691312718 0.8749999691312710 0.5000000000000000

0.6249999691312710 0.8749999691312710 0.5000000000000000

0.8750000308687290 0.6250000308687290 0.5000000000000000

0.8750000308687290 0.1250000308687285 0.5000000000000000

0.6249999691312710 0.3749999691312722 0.5000000000000000

0.1249999691312718 0.3749999691312722 0.5000000000000000

0.3750000308687278 0.1250000308687285 0.5000000000000000

Table S53. Crystallographic data for U_3_Ni_3_Bi_4_ single crystal

| Empirical formula | U_3_Ni_3_Bi_4_ |
| --- | --- |
| Formula weight | 1726.14 |
| Temperature/K | 300 |
| Crystal system | cubic |
| Space group | I-43d |
| a/Å | 9.5719(2) |
| b/Å | 9.5719(2) |
| c/Å | 9.5719(2) |
| α/° | 90 |
| β/° | 90 |
| γ/° | 90 |
| Volume/Å^3^ | 876.99(5) |
| Z | 4 |
| ρ_calcg_/cm^3^ | 13.073 |
| μ/mm^‑1^ | 141.306 |
| F(000) | 2768 |
| Crystal size/mm^3^ | 0.4 × 0.1× 0.1 |
| Radiation | MoKα (λ = 0.71073) |
| 2Θ range for data collection/° | 10.436 to 66.136 |
| Index ranges | -12 ≤ h ≤ 13 |
|  | -14 ≤ k ≤ 14 |
|  | -14 ≤ l ≤ 14 |
| Reflections collected | 3704 |
| Independent reflections | 286 |
|  | R_int_ = 0.0672 |
|  | R_sigma_ = 0.0303 |
| Data/restraints/ parameters | 286/0/9 |
| Goodness-of-fit on F^2^ | 1.141 |
| Final R indexes [I>=2σ (I)] | R_1_ = 0.0166 |
|  | wR_2_ = 0.0333 |
| Final R indexes [all data] | R_1_ = 0.0186 |
|  | wR_2_ = 0.0340 |
| Largest diff. peak/hole / e Å^-3^ | 1.52/-1.37 |
| CCDC | 2517044 |

Table S54. Fractional Atomic Coordinates (×10^4^) and Equivalent Isotropic Displacement Parameters (Å^2^×10^3^) for U_3_Ni_3_Bi_4_. U_eq_ is defined as1/3 of the trace of the orthogonalized U_IJ_ tensor.

| Atom | *x* | *y* | *z* | U(eq) |
| --- | --- | --- | --- | --- |
| Bi1 | 4172.4(4) | 5827.6(4) | 10827.6(4) | 11.10(17) |
| U1 | 3750 | 5000 | 7500 | 11.44(18) |
| Ni1 | 5000 | 7500 | 8750 | 14.9(5) |

Table S55. Anisotropic Displacement Parameters (Å^2^×10^3^) for U_3_Ni_3_Bi_4._The Anisotropic displacement factor exponent takes the form: -2π^2^[h^2^a*^2^U_11_+2hka*b*U_12_+…].

| Atom | U_11_ | U_22_ | U_33_ | U_23_ | U_13_ | U_12_ |
| --- | --- | --- | --- | --- | --- | --- |
| Bi1 | 11.10(17) | 11.10(17) | 11.10(17) | 0.33(10) | -0.33(10) | -0.33(10) |
| U1 | 12.2(3) | 11.0(2) | 11.0(2) | 0 | 0 | 0 |
| Ni1 | 15.6(7) | 15.6(7) | 13.5(11) | 0 | 0 | 0 |

Table S56. Bond Lengths for U_3_Ni_3_Bi_4_

| **Atom** | **Atom** | **Length/Å** |  | **Atom** | **Atom** | **Length/Å** |
| --- | --- | --- | --- | --- | --- | --- |
| Bi1 | Bi1^1^ | 3.5722(3) |  | Bi1 | U1^8^ | 3.3189(4) |
| Bi1 | Bi1^2^ | 3.5722(3) |  | Bi1 | Ni1 | 2.67299(17) |
| Bi1 | Bi1^3^ | 3.5722(3) |  | Bi1 | Ni1^9^ | 2.67299(16) |
| Bi1 | U1^4^ | 3.3070(4) |  | Bi1 | Ni1^10^ | 2.67299(16) |
| Bi1 | U1^5^ | 3.3189(4) |  | U1 | Ni1 | 2.93078(6) |
| Bi1 | U1 | 3.3070(4) |  | U1 | Ni1^11^ | 2.93078(6) |
| Bi1 | U1^6^ | 3.3189(4) |  | U1 | Ni1^9^ | 2.93078(6) |
| Bi1 | U1^7^ | 3.3070(4) |  | U1 | Ni1^12^ | 2.93078(6) |
| ^1^1/2-X,+Y,2-Z; ^2^+X,1-Y,5/2-Z; ^3^1-X,3/2-Y,+Z; ^4^1-Y,-1/2+Z,3/2-X; ^5^-1/2+Z,1/2+X,1/2+Y; ^6^1/2-X,1-Y,1/2+Z; ^7^+Z,1-X,3/2-Y; ^8^+Y,+Z,1+X; ^9^-1/2+Y,-1/2+Z,1/2+X; ^10^3/2-Z,1-X,1/2+Y; ^11^-1/2+Y,3/2-Z,1-X; ^12^1-X,-1/2+Y,3/2-Z | | | | | | |

| 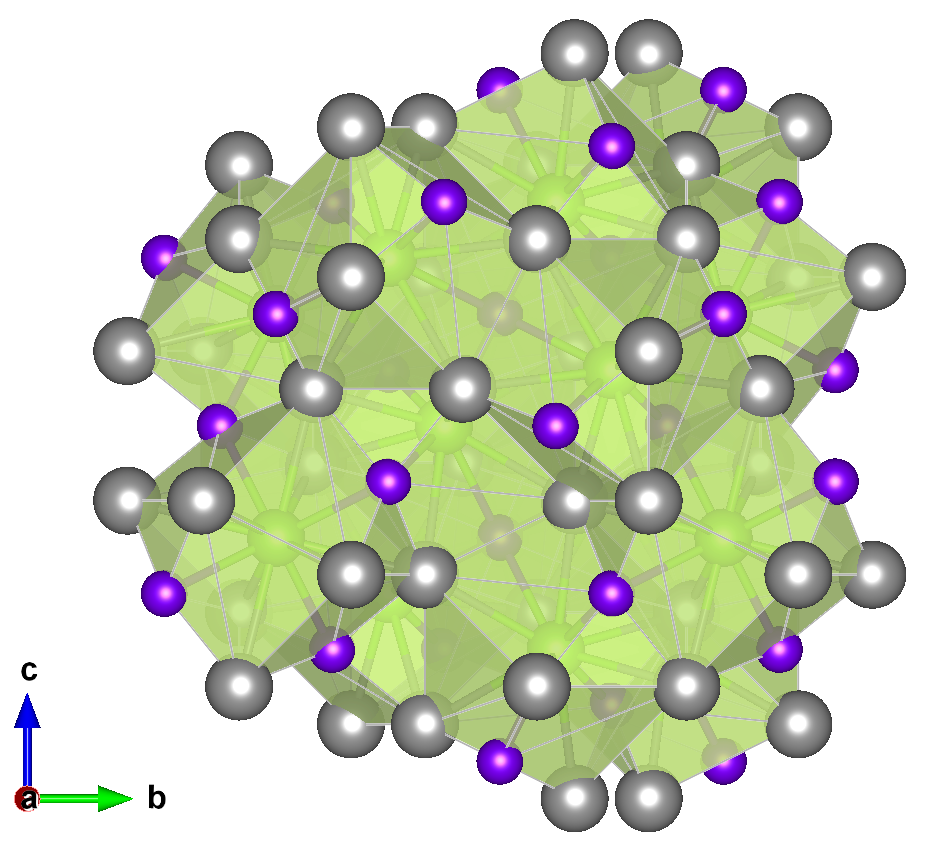 | 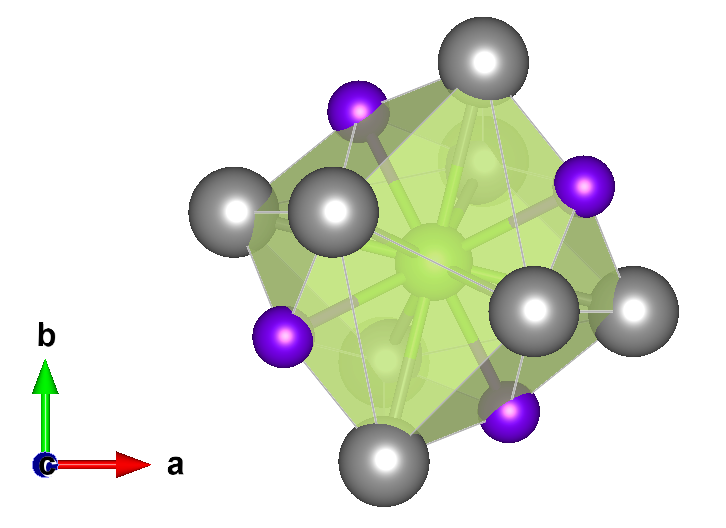 |
| --- | --- |
| (a) | (b) |
| 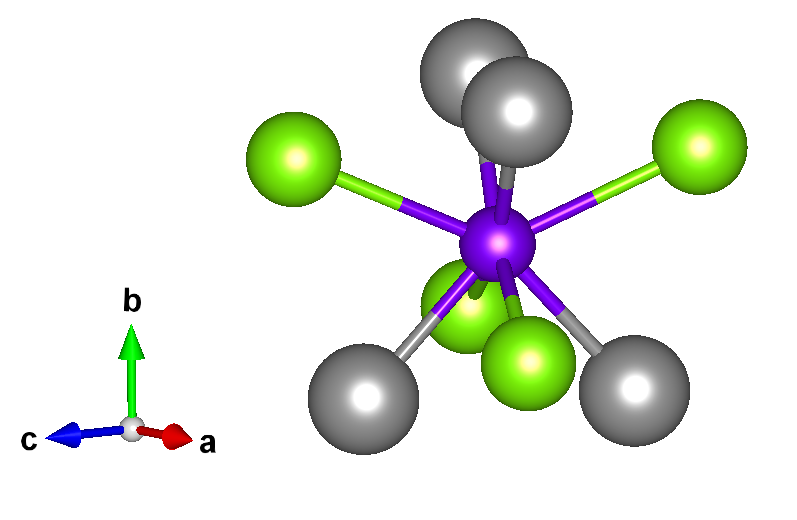 | 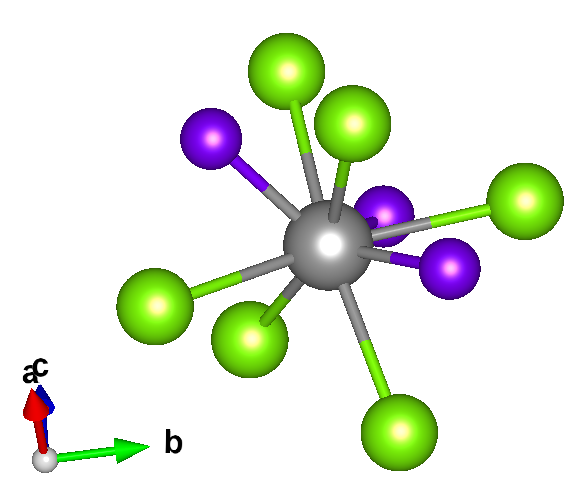 |
| (c) | (d) |

Figure S61. U_3_Ni_3_Bi_4_ a) crystal structure, b) U polyhedra, c) Ni coordination environment, and d) wherein U atoms are green, Ni purple, and Sb grey/silver.

| 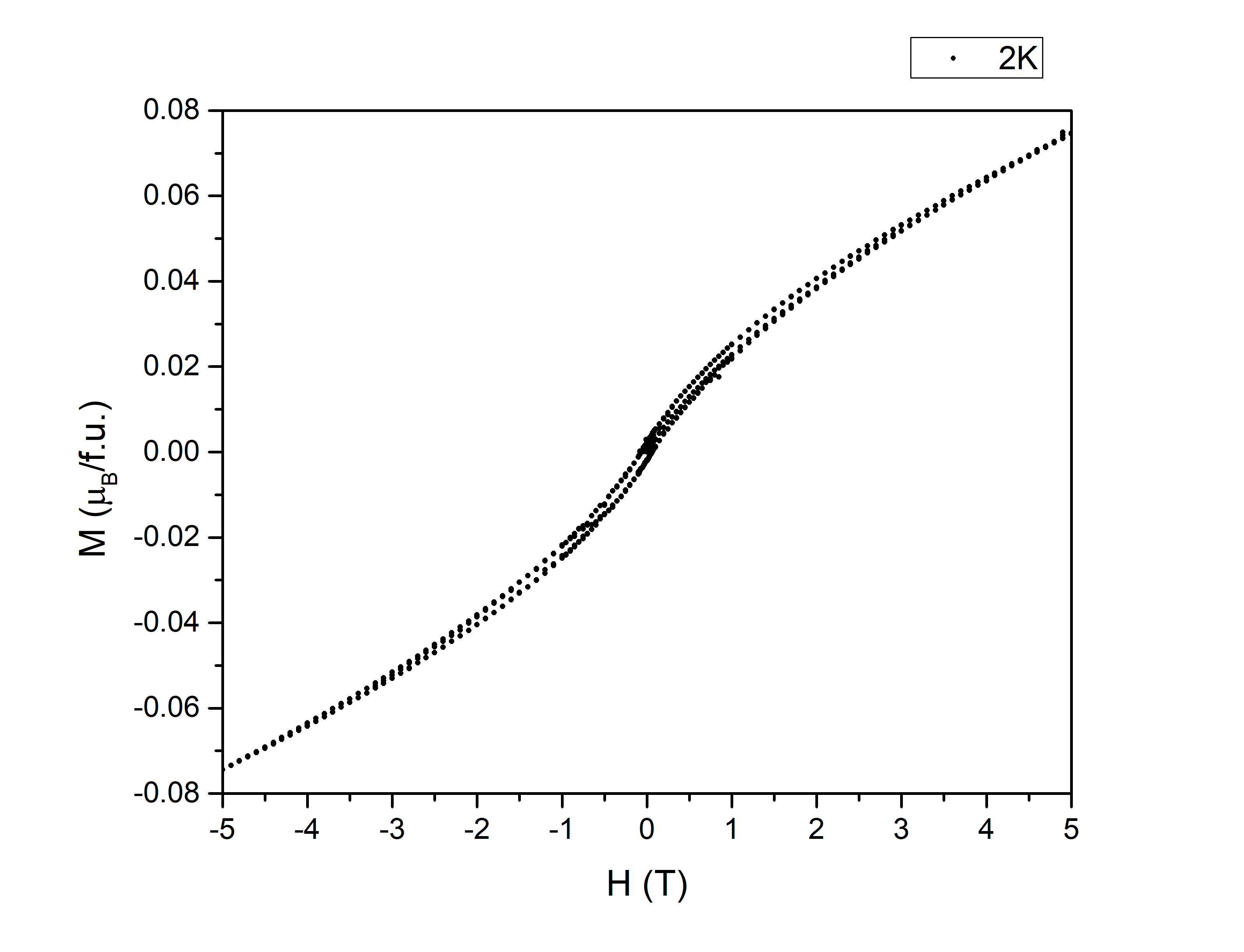 |
| --- |

Figure S62. Magnetization as a function of applied magnetic field at 2K of a UCo_0.4_Bi_2_ powder sample

|  |
| --- |
| 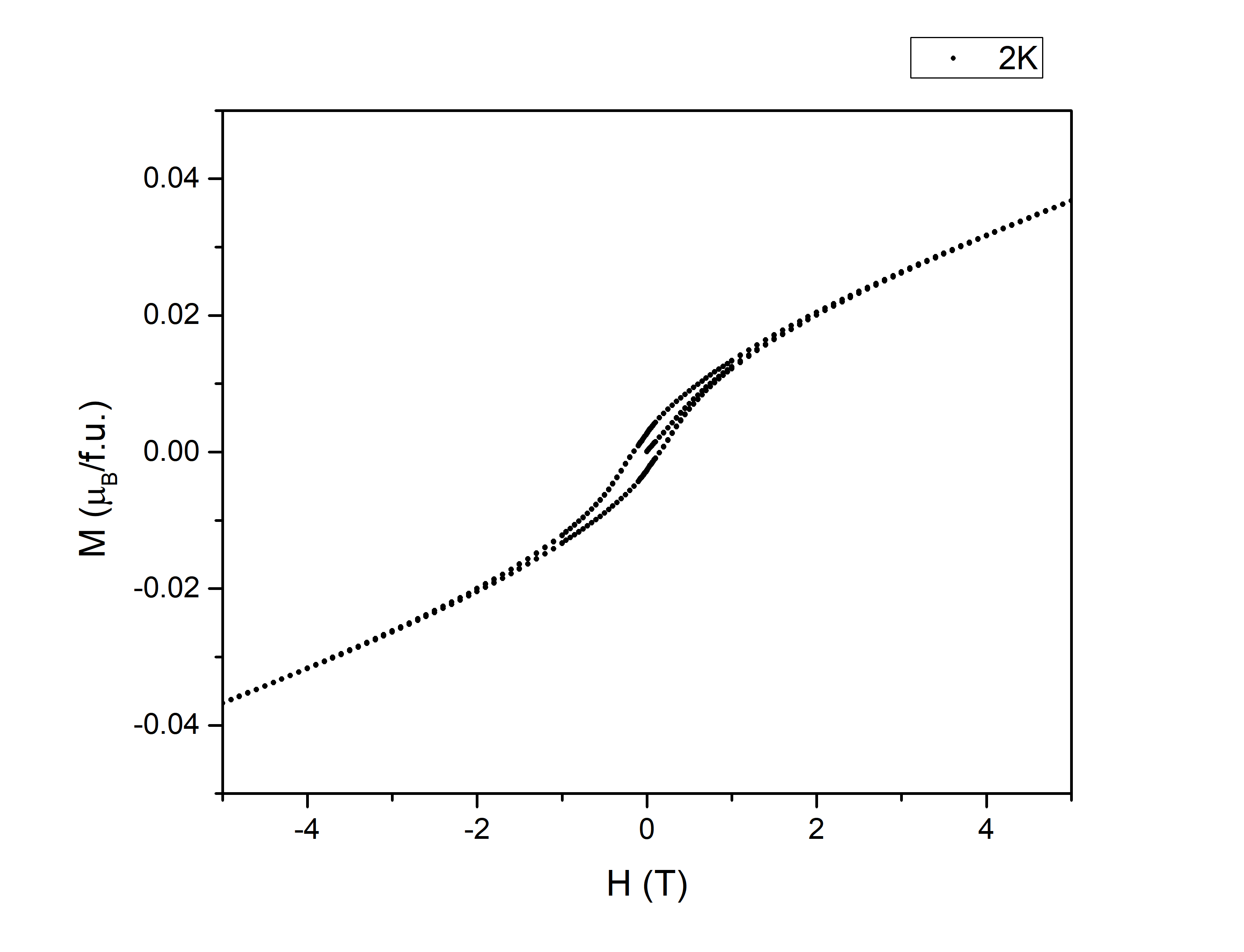 |

Figure S63. Magnetization as a function of applied magnetic field at 2K of a UNi_0.1_Bi_2_ powder sample.

| 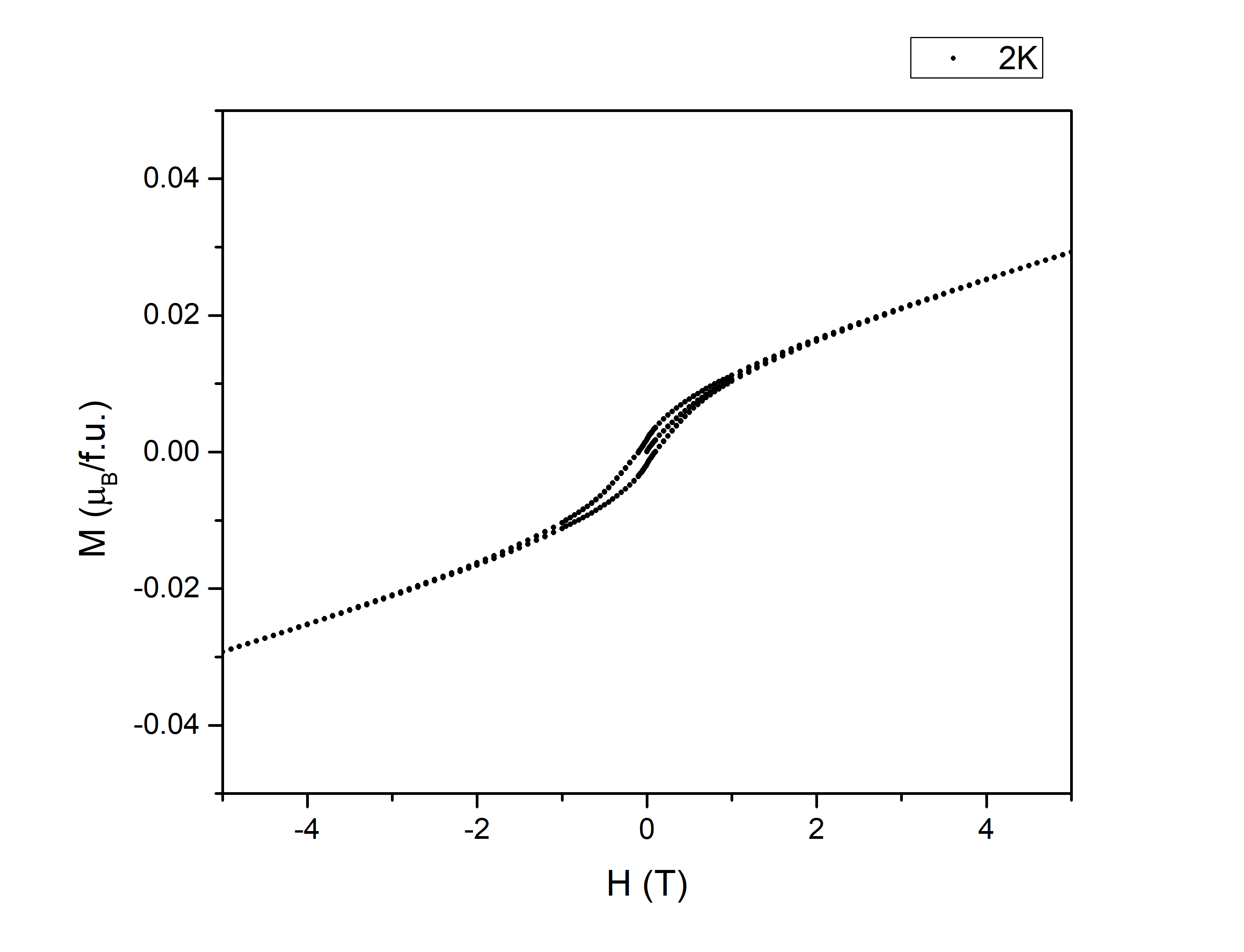 | 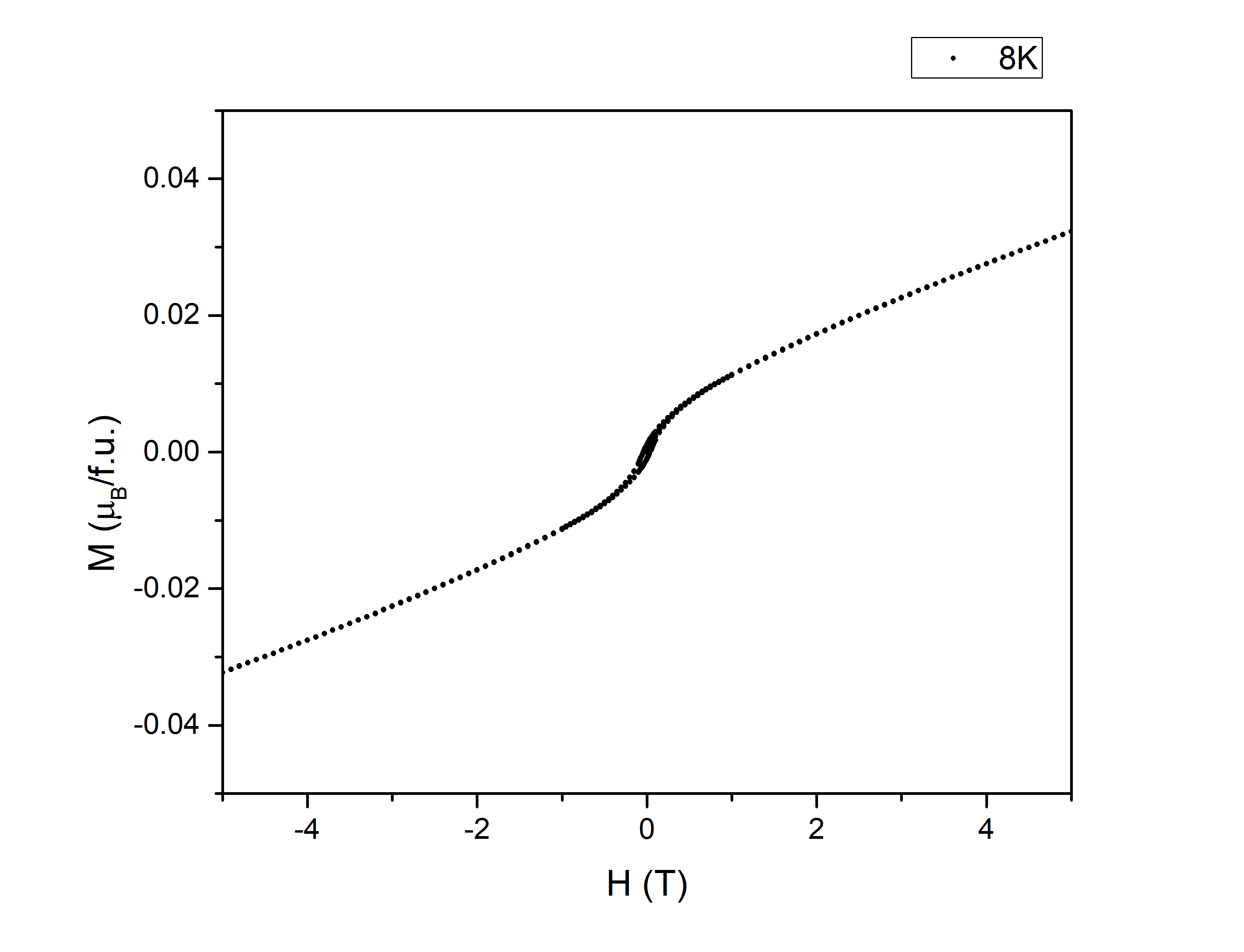 |
| --- | --- |
| (a) | (b) |
| 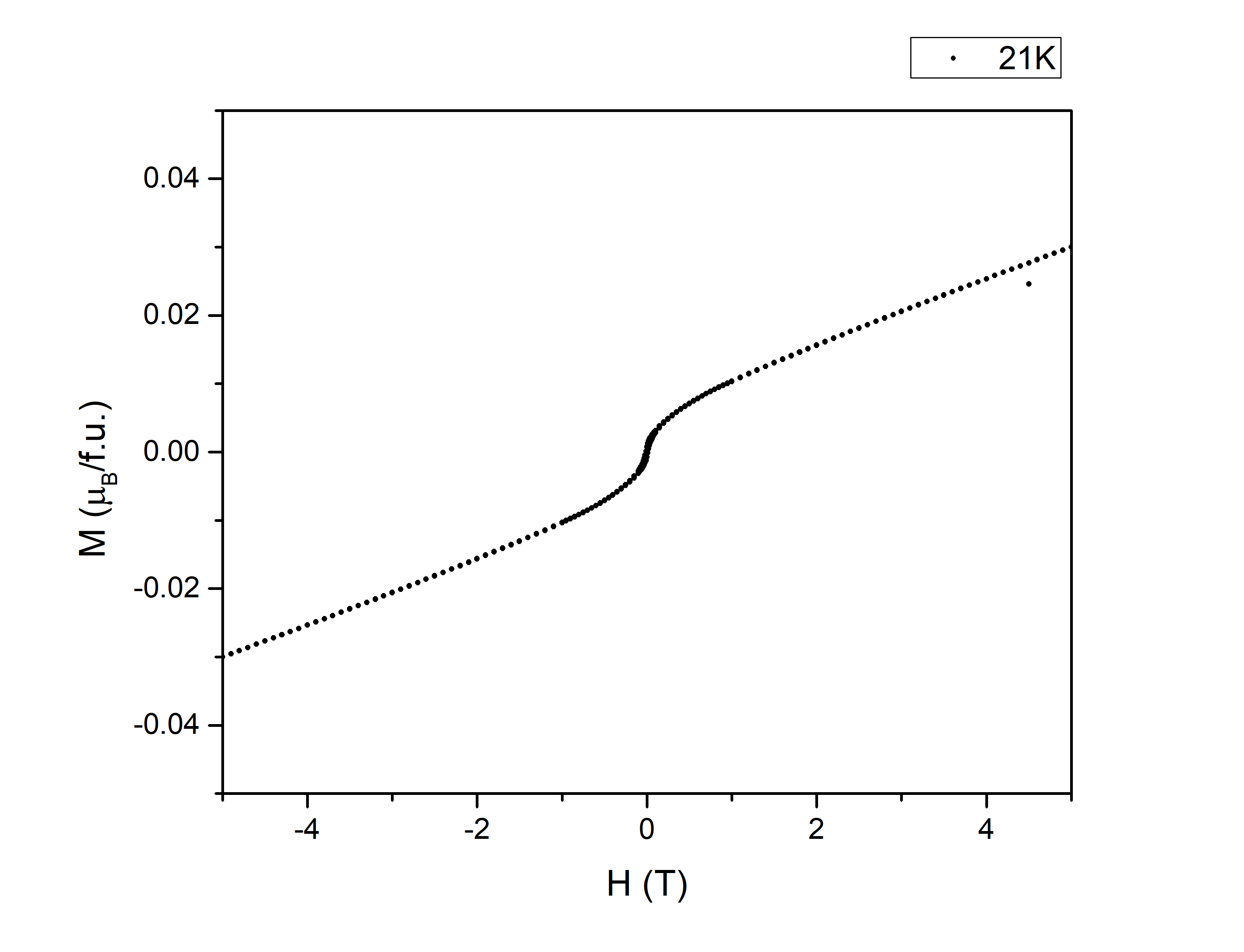 | 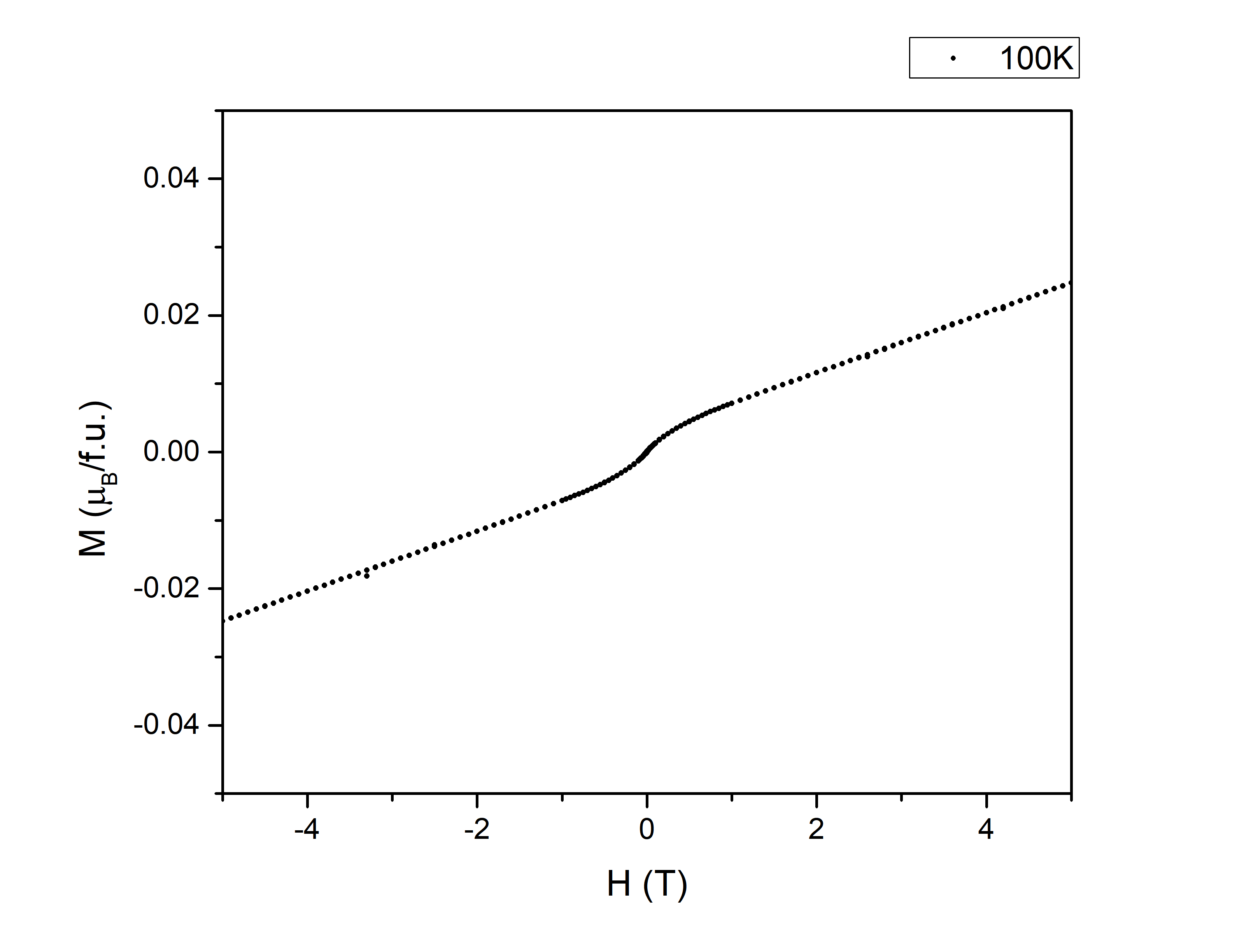 |
| (c) | (d) |

Figure S64. Magnetization as a function of applied magnetic field at a) 2K, b) 8K, c) 21K, and d) 100K of a UNi_0.2_Bi_2_ powder sample.

**References**

(1) Morrison, G.; Zur Loye, H.-C. Simple Correction for the Sample Shape and Radial Offset Effects on SQUID Magnetometers: Magnetic Measurements on Ln2O3 (Ln=Gd, Dy, Er) Standards. *Journal of Solid State Chemistry* **2015**, *221*, 334–337. https://doi.org/10.1016/j.jssc.2014.10.026.

(2) Kresse, G.; Furthmüller, J. Efficiency of Ab-Initio Total Energy Calculations for Metals and Semiconductors Using a Plane-Wave Basis Set. *Computational Materials Science* **1996**, *6* (1), 15–50. https://doi.org/10.1016/0927-0256(96)00008-0.

(3) Kresse, G.; Furthmüller, J. Efficient Iterative Schemes for *Ab Initio* Total-Energy Calculations Using a Plane-Wave Basis Set. *Physical Review B* **1996**, *54* (16), 11169–11186. https://doi.org/10.1103/PhysRevB.54.11169.

(4) Perdew, J. P.; Burke, K.; Ernzerhof, M. Generalized Gradient Approximation Made Simple. *Physical Review Letters* **1996**, *77* (18), 3865–3868. https://doi.org/10.1103/PhysRevLett.77.3865.

(5) Blöchl, P. E. Projector Augmented-Wave Method. *Physical Review B* **1994**, *50* (24), 17953–17979. https://doi.org/10.1103/PhysRevB.50.17953.

(6) Kresse, G.; Joubert, D. From Ultrasoft Pseudopotentials to the Projector Augmented-Wave Method. *Physical Review B* **1999**, *59* (3), 1758–1775. https://doi.org/10.1103/PhysRevB.59.1758.

(7) Wang, V.; Xu, N.; Liu, J.-C.; Tang, G.; Geng, W.-T. VASPKIT: A User-Friendly Interface Facilitating High-Throughput Computing and Analysis Using VASP Code. *Computer Physics Communications* **2021**, *267*, 108033. https://doi.org/10.1016/j.cpc.2021.108033.

(8) SAINT, 2012.

(9) Krause, L.; Herbst-Irmer, R.; Sheldrick, G. M.; Stalke, D. Comparison of Silver and Molybdenum Microfocus X-Ray Sources for Single-Crystal Structure Determination. *Journal of Applied Crystallography* **2015**, *48* (1), 3–10. https://doi.org/10.1107/S1600576714022985.

(10) Dolomanov, O. V.; Bourhis, L. J.; Gildea, R. J.; Howard, J. A. K.; Puschmann, H. OLEX2: A Complete Structure Solution, Refinement and Analysis Program. *Journal of Applied Crystallography* **2009**, *42* (2), 339–341.

(11) Sheldrick, G. M. Crystal Structure Refinement with *SHELXL*. *Acta Crystallographica Section C Structural Chemistry* **2015**, *71* (1), 3–8. https://doi.org/10.1107/S2053229614024218.
